# Cloning and functional complementation of ten *Schistosoma mansoni* phosphodiesterases expressed in the mammalian host stages

**DOI:** 10.1371/journal.pntd.0008447

**Published:** 2020-07-30

**Authors:** Jane C. Munday, Stefan Kunz, Titilola D. Kalejaiye, Marco Siderius, Susanne Schroeder, Daniel Paape, Ali H. Alghamdi, Zainab Abbasi, Sheng Xiang Huang, Anne-Marie Donachie, Samia William, Abdel Nasser Sabra, Geert Jan Sterk, Sanaa S. Botros, David G. Brown, Charles S. Hoffman, Rob Leurs, Harry P. de Koning

**Affiliations:** 1 Institute of Infection, Immunity and inflammation, College of Medical, Veterinary and Life Sciences, University of Glasgow, United Kingdom; 2 Division of Medicinal Chemistry, Amsterdam Institute for Molecules, Medicines and Systems, Vrije Universiteit Amsterdam, The Netherlands; 3 School of Biosciences, University of Kent, United Kingdom; 4 Biology Department, Boston College, Chestnut Hill, Massachusetts, United States of America; 5 Department of Pharmacology, Theodor Bilharz Research Institute, Warrak El-Hadar, Imbaba, Egypt; University of Cambridge, UNITED KINGDOM

## Abstract

Only a single drug against schistosomiasis is currently available and new drug development is urgently required but very few drug targets have been validated and characterised. However, regulatory systems including cyclic nucleotide metabolism are emerging as primary candidates for drug discovery. Here, we report the cloning of ten cyclic nucleotide phosphodiesterase (PDE) genes of *S*. *mansoni*, out of a total of 11 identified in its genome. We classify these PDEs by homology to human PDEs. Male worms displayed higher expression levels for all PDEs, in mature and juvenile worms, and schistosomula. Several functional complementation approaches were used to characterise these genes. We constructed a *Trypanosoma brucei* cell line in which expression of a cAMP-degrading PDE complements the deletion of TbrPDEB1/B2. Inhibitor screens of these cells expressing only either SmPDE4A, TbrPDEB1 or TbrPDEB2, identified highly potent inhibitors of the *S*. *mansoni* enzyme that elevated the cellular cAMP concentration. We further expressed most of the cloned SmPDEs in two *pde1*Δ/*pde2*Δ strains of *Saccharomyces cerevisiae* and some also in a specialised strain of *Schizosacharomyces pombe*. Five PDEs, SmPDE1, SmPDE4A, SmPDE8, SmPDE9A and SmPDE11 successfully complemented the *S*. *cerevisiae* strains, and SmPDE7var also complemented to a lesser degree, in liquid culture. SmPDE4A, SmPDE8 and SmPDE11 were further assessed in *S*. *pombe* for hydrolysis of cAMP and cGMP; SmPDE11 displayed considerable preferrence for cGMP over cAMP. These results and tools enable the pursuit of a rigorous drug discovery program based on inhibitors of *S*. *mansoni* PDEs.

## Author summary

Schistosomiasis is a serious and often fatal disease that is caused by infection with parasitic worms of the *Schistosoma* species. It affects several hundreds of millions of people world-wide, mostly in the tropics, through contact of skin with infected water, usually lakes and rivers. Only one drug, praziquantel, exists for its treatment, so resistance to it would leave the disease untreatable and new drugs are urgently needed. Here, we report on a strategy to develop new anti-schistosomal agents by inhibiting the regulatory systems of the worm. Cyclic nucleotides cAMP and cGMP are key regulators of cellular activity, and their activity is determined by enzymes called phosphodiesterases or PDEs, that can degrade them. We identified and cloned the genes coding for ten of these PDEs from *Schistosoma mansoni*, a species causing a very high disease burden. By expressing these genes in specialized cell lines of the yeast *Saccharomyces cerevisiae* and the protozoan parasite *Trypanosoma brucei*, under conditions where growth becomes conditional on the expression of the introduced *Schistosoma* PDE, we show that most of these genes indeed code for functional PDEs. For several PDEs we also determined, using a third expression system, the yeast *Schizosaccharomyces pombe*, whether they regulate cAMP or cGMP. We also identify a number of inhibitors for one of these PDEs and show that all are expressed more in male than in female worms. This work is essential to map and characterise the regulatory pathways of schistosomes and will facilitate the development of inhibitors of the key enzymes.

## Introduction

Schistosomiasis is caused by parasitic trematodes of the genus *Schistosoma*. Theodor Bilharz first reported on the causes of urinary schistosomiasis in 1851 and the life cycle was described in the early years of the 20^th^ century [1]. One hundred anf fifty years later, it is still a significant disease burden. The transmission cycle includes contact with infected water and is relatively easy to interrupt through proper sanitation, and control of the intermediary host, freshwater snails of the family Planorbidae [2, 3]. Yet, according to the most recent figures of the WHO, at least 220 million people are infected with these blood flukes and as 20 million people suffer severe consequences as a result of advanced schistosomiasis, including an estimated 200,000 annual deaths as of 2000 [4], although this should by now have reduced substantially.

Currently the only available drug against schistosomiasis is praziquantel (PZQ) [5], first introduced in the late 1970s [6]. It is considered to be an effective treatment with single-dose activity against all human-infective schistosomes [7]. However, the treatment does have some limitations, including inactivity against juvenile worms, i.e. recent infections, and, in many patients, incomplete clearance of the infection [5, 8, 9]. In addition, there are multiple reports that some level of PZQ resistance may already exist in the field [10, 11], due to repeated mass administration programs [12]. It would be potentially catastrophic to lose the only treatment for this fatal disease, which is already killing hundreds of thousands of people [13, 14]. However, there is currently hardly even the beginning of a drug-discovery pipeline for schistosomiasis.

It is thus clear that the development of additional treatment options is urgent. One promising avenue for antischistosomal drug discovery is to target the trematode’s regulatory systems, such as receptors, ion channels, and signal transduction mechanisms. These are sufficiently different from human systems to have a potentially different inhibitor profile, yet are sufficiently conserved to be recognizable in the genome and to be susceptible to repurposing of existing compound classes. One such example is PZQ itself, which is believed to target *Schistosoma* calcium channels (*Doenhoff et al*., *2008*), and a number of studies have started the characterization of key *Schistosoma* G-protein coupled receptors [15–20]. Of particular interest are serotonin receptors that are widely distributed in the schistosomal nervous system and induce elevated levels of cellular cAMP when stimulated [17], and for which a distinct pharmacology has started to emerge [19, 20].

The cyclic nucleotides cAMP and cGMP are key regulators in schistosomes. As early as 1973, strong activities of adenylate cyclase, cAMP and cGMP phosphodiesterases (PDEs) and cyclic nucleotide-stimulated protein kinases were identified and some inhibitors of these enzymes were identified—indeed, serotonin was shown to stimulate the adenylate cyclase activity in *S*. *mansoni* [21]. However, until recently little progress was made in understanding the various roles of cAMP in schistosome physiology, or in characterizing any of the cAMP pathway components. Taft and coworkers showed that, as in many protozoa [22, 23], a key life cycle progression in *S*. *mansoni*, from miracidium to primary sporocyst, was coupled to regulation via cAMP [24]. A cAMP-activated kinase, PKA, was identified, cloned and found to be essential for schistosome survival, using both inhibitors and RNAi knockdown [25]. PKA was found to have an important role in regulating motor activity, with its stimulation resulting in hypermotility of the adult worms [26]. Similarly, Hirst *et al*. also found that PKA modulated motility in cercariae and schistosomula [27].

The essentiality of the cAMP signaling pathways, including PKA, prompted us to extend our work on cAMP phosphodiesterases of protozoan parasites [28–32] to *S*. *mansoni*, anticipating that, as in human-infective protozoa, inhibition of PDEs leads to a fatal increase and imbalance in cellular cAMP levels. Indeed, Caffrey and co-workers recently reported the cloning of several homologues of human PDE4 isoforms, and identified benzoxaborole inhibitors of these enzymes that induced hypermotility in the worms [33]. Moreover, the screening of a small library of potential PDE inhibitors identified numerous hits with activity on worm viability and/or ovipositing *in vitro* as well as reduced worm and egg burdens in a mouse model of *S*. *mansoni* infection [34].

Here, we analyze the *S*. *mansoni* genome for all PDE sequences, classify and name them according to phylogenetic analysis. We report the cloning and sequence verification of 10 of the 11 SmPDEs thus identified and present evidence from functional complementation in different yeast and protozoan systems that most of these are indeed cyclic nucleotide phosphodiesterases; their relative expression in male and female mature and juvenile worms as well as schistosomula was also determined. By complementation of a *Trypanosoma brucei* cell line lacking the essential PDEB1/B2 locus, a cellular system for inhibitor screening of SmPDEs was established, and potent new inhibitors of SmPDE4A were identified.

## Results

### SmPDE amplification and naming

Our searches of the published *S*. *mansoni* genome suggested there was a complement of eleven PDE-encoding genes in this species. Four of these have been described previously as being homologous to human PDE4 [33]. Of the eleven sequences, we have successfully amplified ten of the full coding sequences from adult worm cDNA of Egyptian strain CD (see WormBase ParaSite 14 IDs and GenBank accession numbers in Table 1).

**Table 1:** Systematic naming of SmPDEs.

Gene ID	PDE4NPD Name	Allele or splice variant	Length (bp, aa)	GenBank accession number
Smp_134500	Sm1	sv1	1815; 605	MH457507
		sv2	1818; 606	MH457508
Smp_135500	Sm2 (genome)		877	none
Smp_134140	Sm4A		2097; 699	MH457509
Smp_141980	Sm4B	a1	3066; 1022	MH457510
		a2	3060; 1020	MH457511
Smp_334600	Sm4C		2109; 703	MN442427
Smp_153640	Sm7var	a1	2058; 686	MH457512
		a2	2058; 686	MH457513
Smp_044060	Sm8		1668; 555	MH457514
Smp_197150	Sm9A		1554; 517	MH457515
Smp_146120	Sm9B	a1	2751; 917	MH457516
		a2	2745; 915	MT655752
Smp_342020	Sm9C		1923; 641	MH457517
Smp_179590	Sm11	1	3243; 1081	MH457518
		2	3243; 1081	MH457519

a, allele; sv, splice variant.

**Fig 1:**
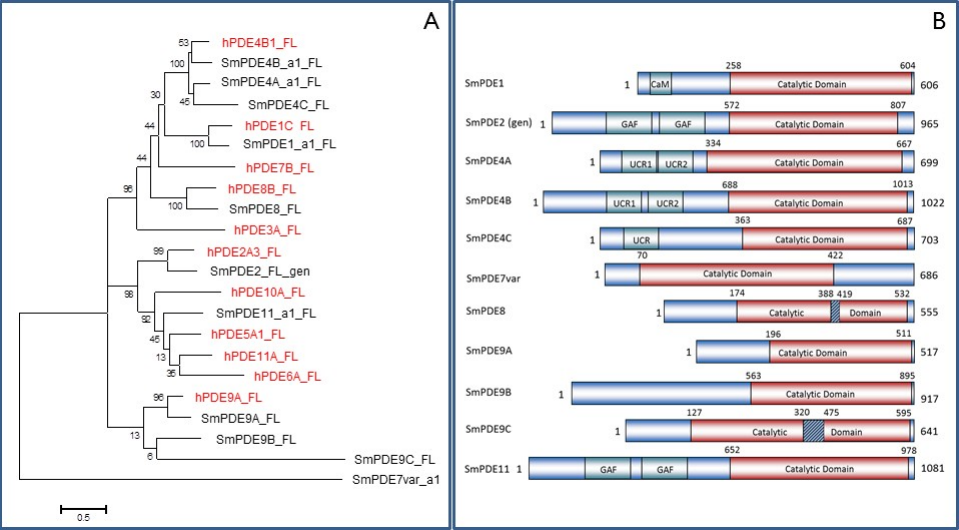
Overview of PDE family genes of *Schistosoma mansoni*. **A**. Phylogenetic tree comparing full length sequences of all SmPDEs to representative members of the human PDE families. Human sequences were taken from Uniprot database, the *S*. *mansoni* genes were from in-house sequencing, unless “gen” is included in the name as this gene is as listed in the *S*. *mansoni* genome (taken from WormBase ParaSite 14). Black = *S*. *mansoni*; red = human. The tree was constructed using a ClustalW alignment [94] and the Maximum Likelihood Tree function of MEGA6, with 500 bootstrap replications, the Nearest-Neighbor-Interchange (NNI) Heuristic Method, and otherwise the default parameters. The percentage of trees in which the associated taxa clustered together is shown next to the branches. The scale bar represents the number of amino acid substitutions per position. **B**. Overview of domain structure of each of the *S*. *mansoni* PDE genes, using the genetic information from the successfully amplified cDNAs where available, or the genomic sequence from WormBase ParaSite 14 (for SmPDE2) where amplification from cDNA had not been successful. Significant inserts in the catalytic domain (compared to mammalian consensus sequence) are indicated by hatched areas. Start and end of the catalytic domain is indicated by amino acid numbering. FL, full length.

A phylogenetic tree was constructed using MEGA6 to analyze the relationship of the SmPDEs to representative members of the human PDE families, in order to derive a systematic naming convention (Fig 1A, Table 1). Our in-house sequence information was used for the ten available genes, with the genomic sequence used for the remaining one (SmPDE2). Three PDEs clustered with hPDE4B1 and were previously identified by the group of Conor Caffrey, using the available genomic sequence from a Puerto Rican *S*. *mansoni* isolate available in GeneDB [33], so we have maintained the naming convention of SmPDE4A-C for these genes. Our sequences for the PDEs differed from those adopted by the Caffrey group. Of note is that our sequence for SmPDE4A was 73 amino acids longer than the sequence previously reported [33], although there were only two SNPs over the length of the reported sequence; the difference being a single 219 bp insert after position 649. Our sequence of SmPDE4B was of equal length with the reported one, and 99% identical at the nucleotide level, whereas their sequence of SmPDE4C (Smp_129270) contained a different first exon and lacked what is exon 4 in the new WormBase ParaSite 14 (WBPS14) sequence (Smp_334600.1e4) and our experimentally obtained sequence, which, as a consequence is much longer than Smp_129270 (703 vs 454 aa, respectively).

The gene previously identified as SmPDE4D [33] clustered separately with hPDE8B and we therefore propose this gene to be renamed as SmPDE8. We found an unannotated exon between the annotated exons 1 and 2, and that the actual start codon was 50 aa upstream from the originally proposed start codon, giving rise to a protein of 555 aa compared to 482 aa proposed previously.

We also found three genes that cluster with hPDE9A, and have named these SmPDE9A-C. One SmPDE clustered with hPDE1C, and has thus been named SmPDE1; one, clustering with hPDE2A3, was designated SmPDE2 but amplification from cDNA of our *S*. *mansoni* strain CD was unsuccessful. The remaining two genes did not clearly cluster with any of the human PDE sequences, and we therefore identified previously named homologues to maintain a logical naming convention. Using protein-protein BLAST searches we assigned the names of SmPDE11 and SmPDE7var, with the family-number assigned based on the top hits of *S*. *haematobium* PDEs—“dual 3',5'-cyclic-AMP and-GMP phosphodiesterase 11” (MS3_05265) for SmPDE11 and “high affinity cAMP-specific 3',5'-cyclic phosphodiesterase 7A” (MS3_06540) for SmPDE7var. SmPDE7var was so named as it appears to be more closely related to PDE7s than to anything else, but it is clearly unusual based on the phylogenetic analysis. The “variant” name is analogous to that used for calcium channel β subunits in *S*. *mansoni* [35].

Despite extensive work, it was not possible to amplify the coding regions of the single remaining SmPDE, SmPDE2, from mammalian lifecycle stage cDNA (schistosomula, juvenile male/female worms or adult male/female worms). Using genomic DNA, it was possible to amplify a fragment of the 5’ UTR extending into the first exon of SmPDE2 (which was identical to published genome sequence), and from the 3’ UTR to the last exon, suggesting the gene could be present and expressed in different lifecycle stages for which we did not have cDNA available. Additionally, it may be that the sequence is quite different in strain CD compared to the genomic Puerto Rican NMRI strain, as we were not able to amplify any of the other predicted exons in the middle of the gene from gDNA. Efforts to amplify this gene from cDNA obtained from *S*. *mansoni* CD cercariae are ongoing.

### Analysis of the SmPDE sequences

For most SmPDEs that were successfully sequenced we found differences to the genomic sequences given in WBPS14; most of these changes were outside the predicted catalytic domain. All changes relative to the genome sequences are summarized in Table 2, illustrated with alignments to the genome sequences in S1 Fig, summarized schematically in Fig 1B, and discussed below. Following the initial identification of *S*. *mansoni* PDEs both sequence and predicted structure alignments were used to validate intron and exon boundaries to define the PDE catalytic domains.

**Table 2:** Coding sequences differences between predicted genome sequence from WormBase ParaSite 14 and sequence obtained from cDNA from Cairo strain CD of *S*. *mansoni*.

Gene	Size	Differences found in CDS from genome
**Sm1**	605/606 aa	One allele found, but with a small splice variant–one has a glutamine inserted at start of exon 8, inside the catalytic domain. Both wobble splice variants exist in WBPS14, as Smp_134500.1 and Smp_134500.1.
**Sm2**	833 or 965 aa (genome)	Amplification unsuccessful from cDNA from the developmental stages available. WBPS14 ID is Smp_135500.1 and Smp_135500.2.
**Sm4A**	699 aa	1 allele found, sequence identical to WBPS14 except for splice variations: a small exon 1 in our sequence is located >26kb upstream of the predicted sequence. All other exons are found in at least 1 of the reported transcripts Smp_134140.1 –Smp_134140.5.
**Sm4B**	1022 / 1020 aa	Two alleles found. Allele SmPDE4B-1 is identical to Smp_141980.1. SmPDE4B-2 is different only by replacing 585-Ile-Asn-Ser in exon 4 with 585-Cys, resulting in the shortening of the sequence by two aa.
**Sm4C**	703 aa	1 allele found. The deduced amino acid sequence differs from the single variant in WBPS14 (Smp_334600) at three positions: two SNPs in the N-terminal regulatory domain and a short glutamic acid stretch adjoining the catalytic domain varies in length by 1 residue.
**Sm7var**	686 aa	Two alleles found. We found, compared to the reference genome (Smp_153640.2), one heterozygous and two homozygous single nucleotide substitutions, all non-synonymous and outside the catalytic domain. Unusually, the catalytic domain is predicted to be towards the N-terminal half of this PDE, alone amongst the SmPDEs.
**Sm8**	555 aa	One allele found, 270 amino acids shorter than the WBPS14 entry (Smp_044060). Amino acid residues 48–55 differ from the database entry due to an extended exon 7, which fully replaces the predicted exon Smp_044060.1.e8. This difference is outside the catalytic domain.
**Sm9A**	517 aa	One allele found, different by two synonymous substitutions from Smp_197150.1.
**Sm9B**	917 / 915 aa	Two alleles found, one identical to Smp_146120.1, in the other three residues of a stretch of Asp residues have been replaced by one Thr residue.
**Sm9C**	641 aa	We found one allele. Significant differences from its WBPS14 entry (Smp_342020.1 and 2): extra N-term Met residue; lacking an Ile-rich 23 aa stretch found in both reference alleles (position 201) that is predicted to disrupt helix 6 of the catalytic domain; unusual 159 aa insert in the catalytic domain, predicted to be located in the loop between helices 12 and 13; exon 8 is extended compared to the exons Smp_342020.1.e8 and 2.e8 of the database variants leading to a shorter and different C-terminus as exon Smp_342020.1.e9 is not used.
**Sm11**	1081 a.a.	Two allelic isoforms found, differing by 4 amino acids (non-synonymous SNPs) outside the catalytic domain; allele SmPDE11-a1 is identical with the database variant Smp_179590.1 at CDS and aa level.

One allele was found for SmPDE1 (Smp_134500), but with a minor splice variation causing a difference of a single glutamine residue at the start of predicted exon 7, inside the catalytic domain (NAGNAG wobble splicing [36]). Both of the splice variants (SmPDE1-sv1 and SmPDE1-sv2) are found in WBPS14, as Smp_134500.1 and Smp_134500.2. From the sequencing traces we concluded that the longer variant (SmPDE1-sv2) is more highly expressed, in agreement with the preferrence in simalar human splice sites [36]. Both TDR targets (tdrtargets.org) and GeneDB (genedb.org) list this gene product as a calcium:calmodulin (CaM)-dependent cyclic nucleotide phosphodiesterase and a CaM binding domain was found near the N-terminal end (Fig 1B).

For SmPDE4A only one allelic sequence was found. The sequence coding for the PDE is identical to that of the reference genome entry Smp_134140. However, splicing of the identified transcript differs from all the variants annotated in the WormBase/ParaSite database. The first exon is located more than 26 kb upstream of the predicted start of the gene locus, giving it a unique N-terminal sequence (S1 Fig). All other exons are found in at least one of the five database variants, e.g. exons 2–10 are identical with those of variant Smp_134140.5 and the rest with variant Smp_134140.1. Four of the predicted variants (Smp_134140.2 to 5) lack crucial parts of the catalytic domain. As discussed by Long *et al*. [33] this gene (and SmPDE4B) contains two Upstream Conserved Domains (UCRs) (Fig 1B), which are usually involved in dimerisation.

There were two alleles for SmPDE4B, SmPDE4B-a1 and SmPDE4B-a2, each containing the UCR1 and UCR2 domains (Fig 1B), with minimal changes compared to each other, or to the genome reference Smp_141980. Isoform SmPDE4B-a1 is identical to Smp_141980.1; SmPDE4B-a2 contains one Cys residue rather than the sequence Ile-Asn-Ser in exon 4 (S1 Fig), which is not in the catalytic domain.

We found one allele for SmPDE4C. The deduced amino acid sequence differs from the single variant in the WBPS14 database, Smp_334600.1, at three positions: two residues are substituted in the N-terminal regulatory domain and a short glutamic acid stretch adjoining the catalytic domain varies in length by 1 residue (S1 Fig). We found one potential UCR domain, between amino acids 81 and 185 (Fig 1B).

There were again two alleles of SmPDE7var found. SmPDE7var-a1 is identical to Smp_153640.2 except for two non-synonymous SNPs near the C-terminal end; SmPDE7var-a1 contains one additional non-synonymous SNP, at position 478 (S1 Fig). None of these substitutions are located in the catalytic domain, which, unusually, is predicted to be towards the N-terminal half of this PDE–unique amongst the SmPDEs (Fig 1B); no GAF, CaM or UCR domains were identified.

For SmPDE8 only one allele was found. The N-terminus was found to be 270 amino acids shorter than of the single predicted variant in the WBPS14 database, Smp_044060.1. Amino acid residues 48–55 differ from the database entry due to an extended exon 7, which fully replaces the predicted exon Smp_044060.1.e8. This difference is outside the catalytic domain. However, as was previously described [33], SmPDE8 (both the reference and our experimental sequence) has an unusual insertion in its catalytic domain, which, when compared to hPDE8 rather than hPDE4, results in a 31 aa insertion, with a conserved cysteine residue in the middle of the insertion (S1 Fig; Fig 1B).

One allele of SmPDE9A was found, differing from the single database entry (Smp_197150.1) in two synonymous nucleotide substitutions located in exons 4 and 14. The deduced amino acid sequences are therefore identical. Another sequence was amplified from the cDNA, which included an additional exon between exons 5 and 6; however, this insertion introduces a frameshift and a premature stop codon after 194 aa; well before the start of the catalytic domain around 255 aa. For SmPDE9B we found two alleles, one identical to Smp_146120.1, in the other three residues of a stretch of Asp residues have been replaced by one Thr residue.

A single allele was also found for SmPDE9C, which was significantly different from the genome sequence (Smp_342020.1 and 2). Our sequence contained an extra N-terminal methionine residue and lacked an Ile-rich 23 aa stretch found in both reference alleles (position 201) that is predicted to disrupt helix 6 of the catalytic domain and could make the reference protein non-functional. Our sequence and both reference alleles also contained an unusual 156 aa insert in the catalytic domain, compared to hPDE9A. The insert is predicted to be located in the loop between helices 12 and 13 and is itself predicted to contain 7 alpha helices. In our sequence of SmPDE9C exon 8 was extended compared to the exons Smp_342020.1.e8 and 2.e8 of WBPS14, leading to a shorter and different C-terminus as exon Smp_342020.1.e9 is not used, yielding a length of 641 aa for SmPDE9C compared to 756 and 758 for Smp_342020.1 and 2. In order to assess whether this splice variant was expressed in other lifecycle stages, or a splice variant restricted to the adult worms, we completed PCR analysis using cDNA from schistosomula, and juvenile and adult separated sex worms. A single band corresponding to the size of the 3’ half of the extended sequence containing the catalytic domain insertion was found in all stages (S2 Fig). We thus conclude that in both schistosomula, and in juvenile and adult worms of both sexes, the same PDE is expressed.

There were two alleles of 1081 aa found for SmPDE11, which differed by 4 amino acids from each other (none in the catalytic domain), with one sequence, SmPDE11-a1 having the same amino acid sequence as the WBPS14 reference, Smp_179590.1. A matrix of amino acid sequence identity of the *S*. *mansoni* PDE sequences and examplar human and kinetoplastid PDEs is given in S3 Fig; FASTA sequence of all the SmPDEs here reported is given in S1 File (protein coding exons for all SmPDE genes) and S2 File (amino acid sequence).

### Analysis of SmPDE expression levels in *S. mansoni* stages of mammalian hosts

**Fig 2:**
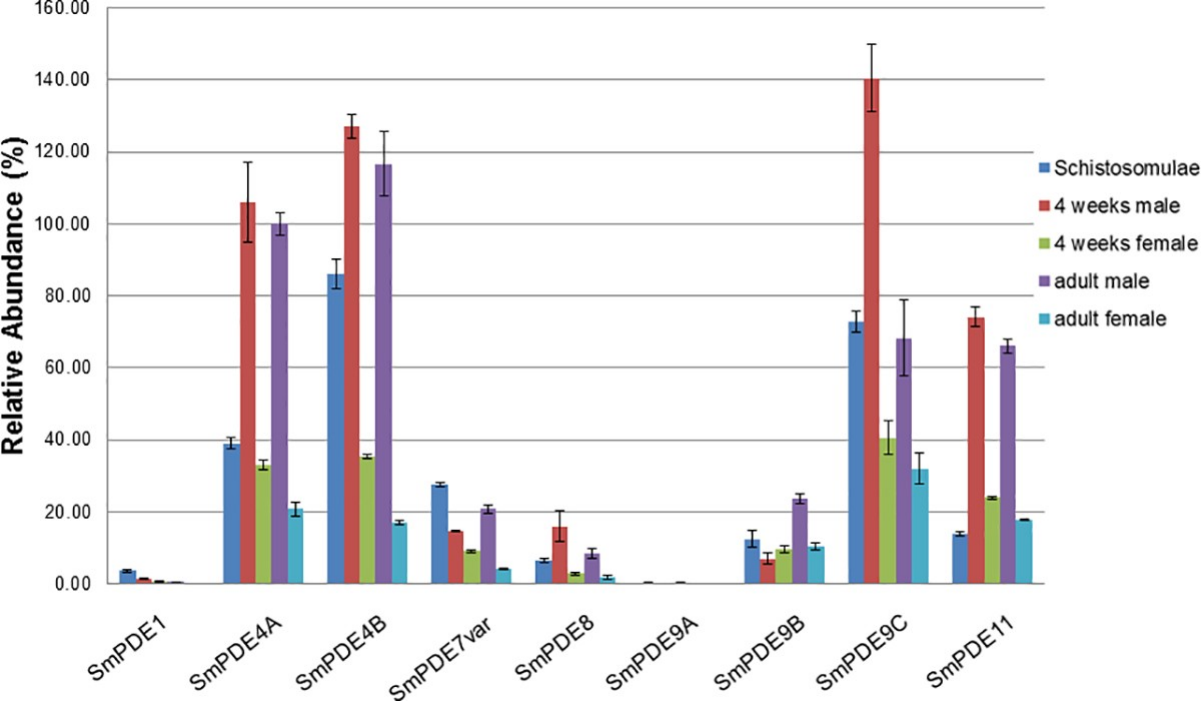
Expression levels of SmPDEs from the *S*. *mansoni* developmental stages that exist in mammalian hosts. Expression of the cloned and sequence-confirmed SmPDEs in cDNA from schistosomula, male and female juvenile worms, and male and female adult worms was determined by qRT-PCR and normalised to the level of expression of the housekeeping gene COX1; the expression level of Sm4A in male adult worms was arbitrarily set as 100% and the entire dataset (all life-cycle stages) expressed as a percentage thereof. cDNA was produced multiple times from Cairo strain *S*. *mansoni* RNA samples; each bar represents the mean and SEM of 4–12 qPCR analyses.

The level of expression of nine amplified and sequenced PDEs was investigated in separate populations of *S*. *mansoni* developmental stages: schistosomula, separated male and female juvenile (4 weeks old) and adult worms. Strikingly, there was a difference in expression level between male and female worms, with male worms having higher expression levels for all PDEs, in both mature and juvenile worms, with the single exception that expression of PDE9B was not significantly different in 4-week old male and female worms (Fig 2). SmPDE4C is not included in this study as it was not yet sequenced when the relative expression of the other nine was determined.

The levels of expression of the PDEs were variable. SmPDE4A, SmPDE4B, SmPDE9C and SmPDE11 displayed the highest levels of expression, which was particularly high in male juvenile and adult worms (relative abundance of between 60–140%, with the level of expression in SmPDE4A adult males set at an abundance of 100). Relative abundance in female worms was 17–40%. The level of expression of all four was substantially lower in the schistosomula stage, with relative abundance of ~40, 85, 75 and 15% respectively. Interestingly, SmPDE9C, which we found to have an unusual catalytic domain insertion, appears to be one of the most highly expressed *S*. *mansoni* PDEs, particularly in juvenile male worms. SmPDE1 and SmPDE9A are hardly expressed in the stages that exist in the mammalian hosts (relative abundance of 0.22–3.7% for SmPDE1 and 0.06–0.35% for SmPDE9A). SmPDE7var, SmPDE8 and SmPDE9B are intermediately expressed, again being more highly expressed in male worms compared to female, but in the range of 2–28%. These results are in close agreement with entries in the online database schisto.xyz [37], which is the searchable interface for *S*. *mansoni* RNAseq results from all life cycle stages and from the male and female gonads [38]. This database did not have entries for SmPDE1 and SmPDE9A but did have entries for the two genes that we did not obtain expression data for, SmPDE2 and SmPDE4C. Remarkably, only these two PDEs were expressed more highly in female adults than male adults. This database also shows that PDEs are poorly expressed in ovaries but many are robustly expressed in testes: PDEs 2, 4A, 4C, 7var, 9C and 11.

### Complementation system in *T. brucei*

**Fig 3:**
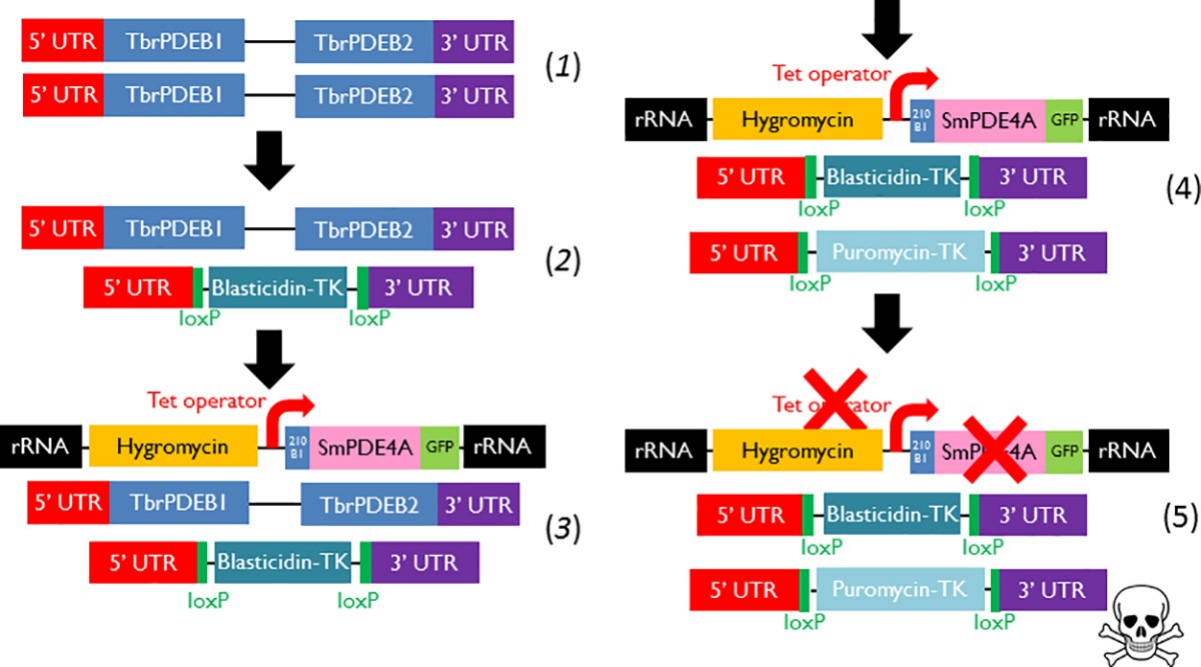
Complementation strategy for *S*. *mansoni* PDEs in *T*. *brucei*. The TbrPDEB1 and TbrPDEB2 genes are in tandem array (*1*), allowing replacement of one pair of alleles with a single antibiotic resistance gene by homologous recombination (*2*). Into this single-KO strain, an exogenous gene is expressed under tetracycline (tet) control from the rRNA locus, with an N-terminal tag consisting of the first 210 bp of TbrB1 (210 B1) to attempt to target the gene to the flagellum (Luginbuehl *et al*. 2010), and a C-terminal GFP tag for expression analysis (3). The second pair of TbrB1/2 alleles can then be deleted if the exogenous gene can complement in the presence of tetracycline induction (*4*). Finally, if tetracycline is removed from the media, the cells should die. The deletion cassettes were based on the work of Kim *et al*. [40]. TK indicates HSV thymidine kinase.

We developed a complementation system in the kinetoplastid parasite *Trypanosoma brucei* in order to investigate the functional capabilities of the SmPDEs (Fig 3). The system is based on the essentiality of a pair of tandemly arranged PDEs in *T*. *brucei*: TbrPDEB1 and TbrPDEB2 [39], and the simultaneous tetracycline-dependent expression of a single heterologous PDE to compensate for the deletion of the essential genes.

To produce this system, first a single knockout cassette was introduced into *T*. *b*. *brucei* (2T1 strain), by homologous recombination, introducing blasticidin resistance, and Herpes Simplex Virus thymidine kinase (HSV-TK) that is necessary to later excise the antibiotic resistance cassette using the loxP motifs [40] (Fig 3, contruct 2) (plasmid pHDK76). Next, a heterologous (*S*. *mansoni*) PDE gene was introduced, with its expression under the control of tetracycline, into the tagged rRNA locus of the *T*. *b*. *brucei* 2T1 cells [41], with the introduced cassette also conferring hygromycin resistance (Fig 3, construct 3). The SmPDEs with which complementation was attempted were SmPDE1, SmPDE4A, SmPDE4B, SmPDE8, SmPDE9A and SmPDE11. In addition, both TbrPDEB1 and TbrPDEB2 were individually introduced into this system as positive complementation controls (plasmid details given in S1 Table).

Following PCR confirmation of the successful introduction of the tet-controlled PDE gene, we attempted to knock out the second pair of TbrPDEB1-B2 alleles using a puromycin-HSV-TK cassette (pHDK82; Fig 3, construct 4). Following the generation of clonal populations from these transfections, by limiting dilution, the clones were also grown in media without tetracycline, which should identify double null cell lines successfully complemented with the heterologous PDE, as the cell survival should depend on the tet-dependent expression of the heterologous PDE (Fig 3, step 5). DNA was extracted for additional confirmation of loss of TbrPDEB1-B2 by PCR.

We were able to successfully generate the control lines, expressing either TbrPDEB1-6Myc or TbrPDEB2-6Myc, and used the cre-lox system [40] to remove the blasticidin and puromycin antibiotic resistance cassettes (dKO+TbrPDEB1 and dKO+TbrPDEB2, respectively). With the lines expressing SmPDE genes, only SmPDE4A was found to complement, with a single clone that did not have either TbrPDEB1 or B2 and had the correct integration of the blasticidin and puromycin resistance cassettes (dKO+SmPDE4A clone 1–3, S4 Fig). All other SmPDEs attempted were unable to complement in this system, with rearrangements of the TbrPDEB1-B2 locus being observed in order to integrate the antibiotic resistance cassettes.

We hypothesized that some of the PDEs may be cGMP specific and so not capable of complementing in cAMP-dependent *T*. *brucei*. Alternatively their level of activity may be intrinsically lower than the native TbrPDEB1/B2 proteins; or perhaps the C-terminal GFP tag may have interfered with their functionality. Moreover, the cellular localization of some of the SmPDEs may have been wrong although all were N-terminally tagged with the first 70 aa of TbrPDEB1, which should have resulted in the flagellar localization [42].

**Fig 4:**
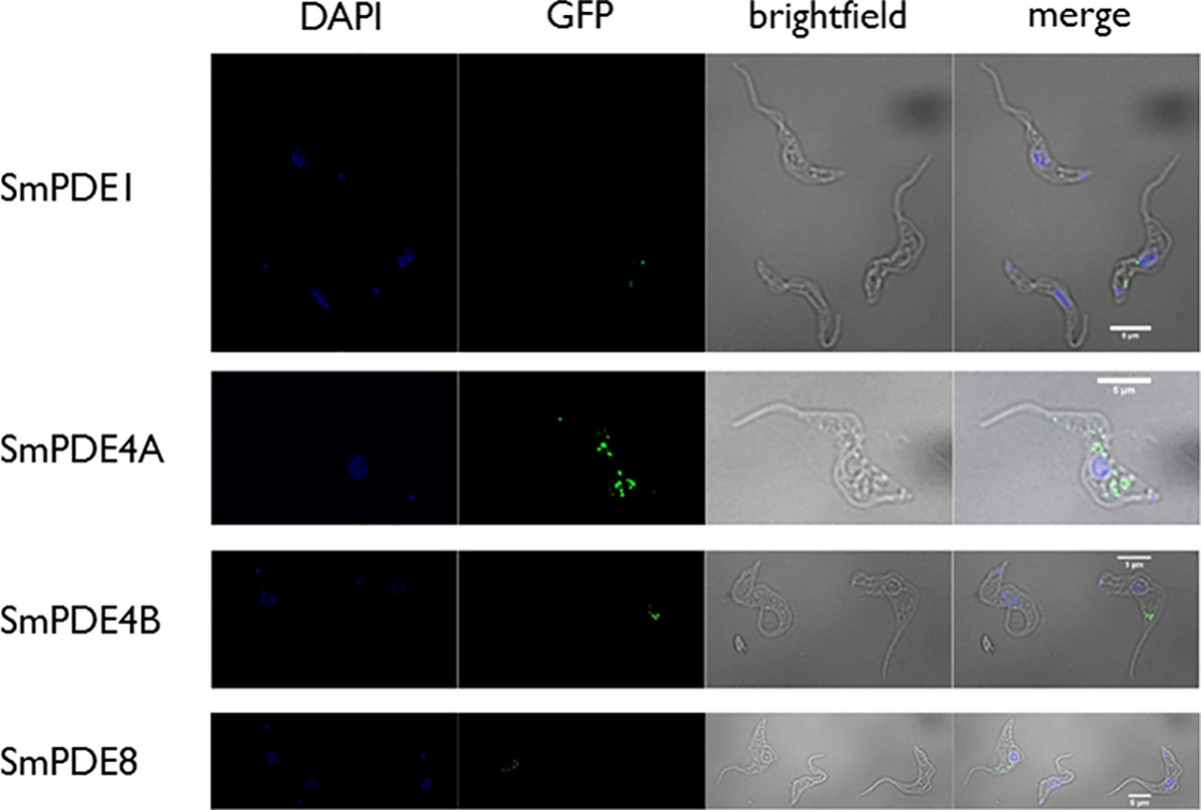
Immuno-localization of SmPDEs after expression in *T*. *brucei*. *T*. *brucei* 2T1 cells from which a single TbrPDEB1/B2 was deleted, and expressing either SmPDE1, SmPDE4A, SmPDE4B or SmPDE8, C-terminally fused to GFP and N-terminally tagged with the first 70 aa of TbrPDEB1, were fixed, and stained with DAPI (blue fluorescence) to reveal the nucleus and kinetoplast. The location of the GFP tag, and thus the position of the SmPDE was revealed using antibodies against GFP (green fluorescence).

However, while this N-terminal sequence may be able to localize some proteins to the flagellum, we found it was not in itself sufficient to do this for the *S*. *mansoni* PDEs, as verified by fluorescence microscopy. For this purpose, the single KO cells, containing one TbrPDEB1/B2 allele plus the SmPDE construct, were stained with DAPI to highlight the nucleus and kinetoplast, and the fluorescence of the GFP-tagged SmPDEs was also observed. It was found that the GFP fluorescence in the SmPDE4A-expressing cells was much more intense, and much more widely distributed throughout the cell body than that of the other SmPDEs (Fig 4). However, even SmPDE4A, which clearly complemented the TbrPDEB1/B2 dKO, was not observed in the flagellum, although it was present in the posterior end near the flagellar pocket; it is possible that the bulky GFP fusion is preventing flagellar localization.

### Pharmacological evaluation of SmPDE4A-expressing *T. brucei*

**Fig 5:**
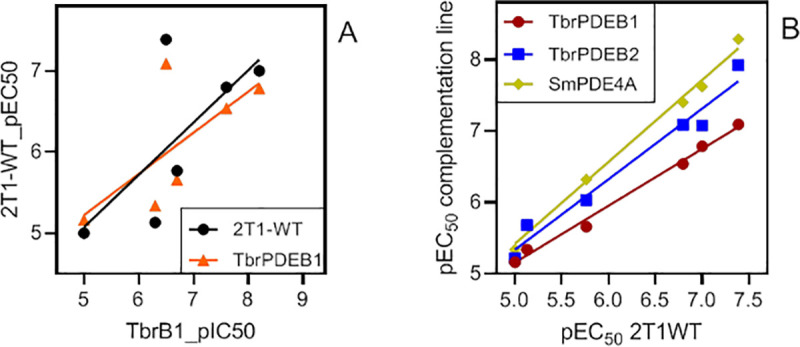
Analysis of the pharmacology of complementation cell lines. **A**. Correlation between the pIC_50_ of inhibition of the purified enzyme TbrPDEB1 and pEC_50_s of the same compounds against either the parental (wild-type) cell line 2T1 (r^2^ = 0.50) or the double knockout strain complemented by TbrPDEB1 (r^2^ = 0.48). Both lines were found not to be significantly non-linear by F-test (Prism 8.4, P>0.999). **B**. Correlation between the pEC_50_ values against 2T1 cells and against the TbrPDEB1/B2 double knockout strain complemented by TbrPDEB1 (r^2^ = 0.993, slope 0.797, P<0.0001), TbrPDEB2 (r^2^ = 0.967, slope 0.990, P<0.001) or SmPDE4A (r^2^ = 0.993, slope 1.148, P<0.0001).

In order to validate the complementation assay for its potential to be used for screening, we selected a small set of known TbrPDEB1 inhibitors, related to the tetrahydrophthalazinone inhibitor NPD-001, and tested them on the wild type as well as on the double KO complemented with TbrPDEB1 or TbrPDEB2. Within this small set of compounds with a >200 fold activity range on the wild type strain (41 nM—9.95 μM), we observed a correlation between enzyme inhibiting potency and activity against the parasite, whether expressing both TbrPDEs or just TbrPDEB1 (Fig 5A), which is in agreement with what we reported previously [30, 43]. This set of compounds shows consistently higher activity against the double knockout strain expressing SmPDE4A (dKO+SmPDE4A) than against the strain expressing TbrPDEB1 (dKO+B1) or TbrPDEB2 (dKO+B2). Most interestingly, the dKO+SmPDE4A cells were highly sensitized to compound NPD-356, with an EC_50_ of 5.2 ± 1.2 nM as opposed to 81 ± 32 (P<0.01, Student’s unpaired t-test) and 12 ± 3 nM (P<0.05) for the same cell lines expressing TbrPDEB1 and TbrPDEB2, respectively. Similarly, NPD-001, NPD-226 and NPD-547 displayed both sub-micromolar activity against dKO+SmPDE4A (EC_50_ of 24 ± 3, 40 ± 4 and 480 ± 30 nM, respectively) as well as substantial selectivity over the *T*. *brucei* PDEs, particularly TbrPDEB1 (P<0.01; Table 3). These results confirm that this complementation assay can be used for screening compound libraries. The EC_50_s for each complementation line (TbrPDEB1, TbrPDEB2, SmPDE4A) all exhibited excellent correlation with the EC_50_s for the wild-type control (Fig 5B), and the same inhibitor rankings, showing that, as expected, all three enzymes display very similar pharmacology. Indeed, both TbrPDEB1 and SmPDE4A have a high similarity with human PDE4 [33, 44, 45] (S3 Fig) and as such each of these inhibitors also inhibits hPDE4B1 (pEC_50_ range 8.4–10.3); it is likely that the successsful complementation by SmPDE4A in *T*. *b*. *brucei* is at least in part due to its similarity to TbrPDEB1. The PDE3-selective compound Trequinsin, which has a low activity against TbrPDEB1 [46], was used as a negative control (EC_50_ > 25 μM for 2T1-WT and all complementation strains), whereas the EC_50_ values of all strains for the positive control, pentamidine, were not significantly different from each other (Table 3).

**Table 3:** Effects of potential PDE inhibitors on parental *T*. *brucei* 2T1 strain and on trypanosomes dependent only on TbrPDEB1, TbrPDEB2 or SmPDE4A.

	2T1-WT EC_50_ (μM)	dKO+TbrPDEB1 EC_50_ (μM)	dKO+TbrPDEB2 EC_50_ (μM)	dKO+SmPDE4A		
				EC_50_ (μM)	RF(B1)	RF(B2)
NPD-001	0.10 ± 0.015	0.165 ± 0.023	0.084 ± 0.018	0.024 ± 0.003	0.14***	0.27**
NPD-029	9.95 ± 0.95	6.9 ± 0.6	6.1 ± 0.7	4.6 ± 0.4	0.67*	0.77*
NPD-226	0.16 ± 0.01	0.29 ± 0.11	0.082 ± 0.009	0.040 ± 0.004	0.13**	0.48*
NPD-356	0.041 ± 0.004	0.081 ± 0.032	0.012 ± 0.003	0.0052 ± 0.0012	0.064**	0.44*
NPD-547	1.72 ± 0.16	2.2 ± 0.6	0.93 ± 0.17	0.48 ± 0.03	0.22**	0.52**
NPD-1014	7.4 ± 1.7	4.6 ± 0.9	4.7 ± 0.3	2.1 ± 0.1	0.45**	0.45**
Trequisin	33.2 ± 0.9	25.8 ± 2.6	25.7 ± 2.4	34.5 ± 2.0	1.34	1.35
Pentamidine	0.0012 ± 0.0002	0.0016 ± 0.0005	0.0013 ± 0.0003	0.0016 ± 0.0003	1.00	1.24

EC_50_ values (μM) calculated by alamar blue assay for selected NPD compounds on *T*. *brucei* 2T1 cells and the same cells expressing inducible copies of SmPDE4A, TbrPDEB1 or TbrPDEB2 instead of the normal TbrB1/2 tandem array (n = 3–9). The ratio of the EC_50_ value for the SmPDE4A-expressing cells and the EC_50_ value for cells expressing either TbrPDEB1 or TbrPDEB2 is known as the relative resistance factor (RF(B1) and RF(B2), respectively); if >1 the compound displays higher activity against the individual *T*. *brucei* PDE, if RF<1 the compound is more active against SmPDE4A. ND, not determined; NA, not applicable. Statistical difference between the EC_50_ values of cells expressing SmPDE4A or either TbrPDEB1 or TbrPDEB2 were determined using Student’s unpaired, two-tailed t-test.

*, P<0.05

**, P<0.01

***, P<0.001.

**Fig 6:**
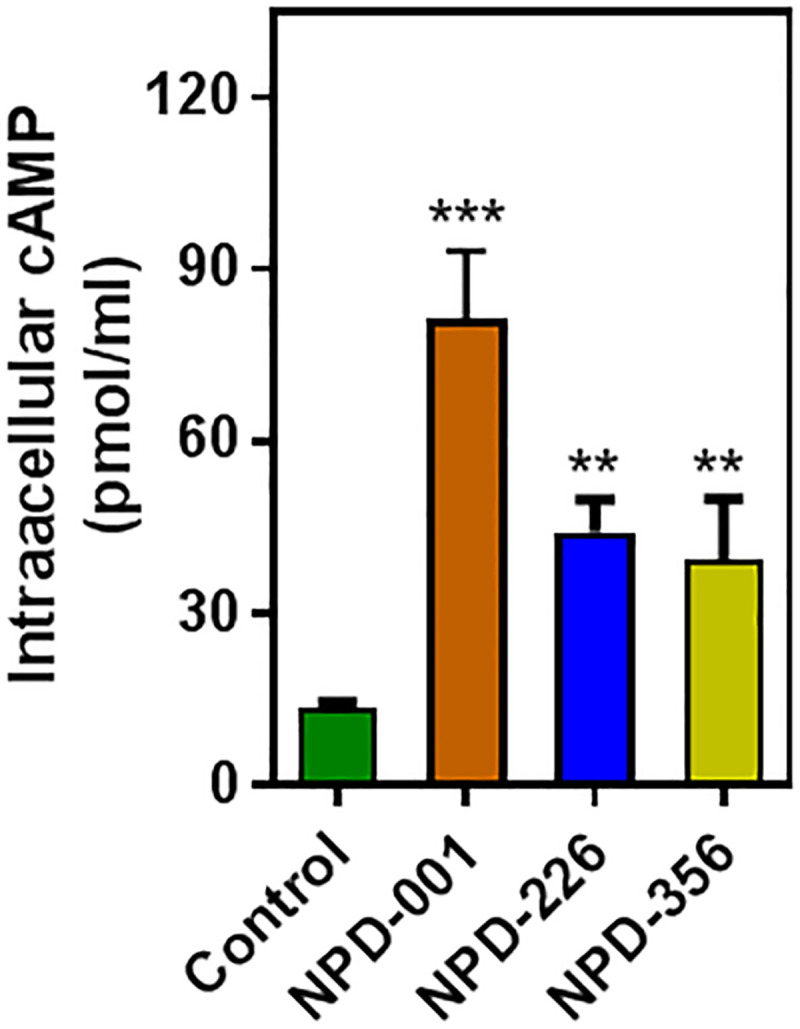
Analysis of intracellular cAMP after incubation of *T*. *brucei* bloodstream forms expressing SmPDE4A (TbrPDEB1^-/-^, TbrPDEB2^-/-^) for 2 h with or without PDE inhibitor (each at a concentration of 2× their EC_50_ value). Each bar represents the average of three independent experiments (each analysed in duplicate) and SEM. Statistical significance was determined relative to the untreated control cells using Student’s unpaired t-test; *, P<0.05; **, P<0.01; ***, P<0.001.

The three most potent inhibitors (NPD-001, NPD-226 and NPD-356) were hypothesized to exert their trypanocidal effects through inhibition of the respective PDEs expressed by each cell line, given the differential effect on cell lines differing only by the PDE expressed. This was tested by incubating dKO+SmPDE4A cells with a modest concentration of 2×EC_50_ of each compound (or no compound, as control) for 2 h. As shown in Fig 6, there was indeed a significant increase in the intracellular cAMP concentrations upon treatment with NPD-226, NPD-356 and NPD-001, when compared to untreated control cells. These results demonstrate the functional replacement of TbrPDEB1 and B2 with the SmPDE4A enzyme and show that such cell lines are potential tools for the screening of compound libraries to identify selective PDE inhibitors in a cellular system.

All the compounds were also tested for antischistosomal effects in vitro at a range of 5–100 μM, displaying varying activities (S2 Table) that poorly correlate with their IC_50_ values against SmPDE4A. For instance, NPD-001 was a highly active inhibitor of the enzyme expressed in *T*. *brucei*, with IC_50_ 0.10 ± 0.015 μM, but had no discernable impact on worm viability, movement, coupling or ovipositing up to 50 μM; similarly NPD-227 had no effect on worm viability but induced spastic contractions at 100 μM. In contrast, NPD-356 and NPD-1014 displayed killing of male worms at concentrations of 25 and 50 μM, respectively, and impacted ovipositing at 5 μM. Trequinsin had no effect on schistosome viability but did impact worm coupling and ovipositing, especially at the higher concentrations.

### SmPDE functionality in yeast

**Fig 7:**
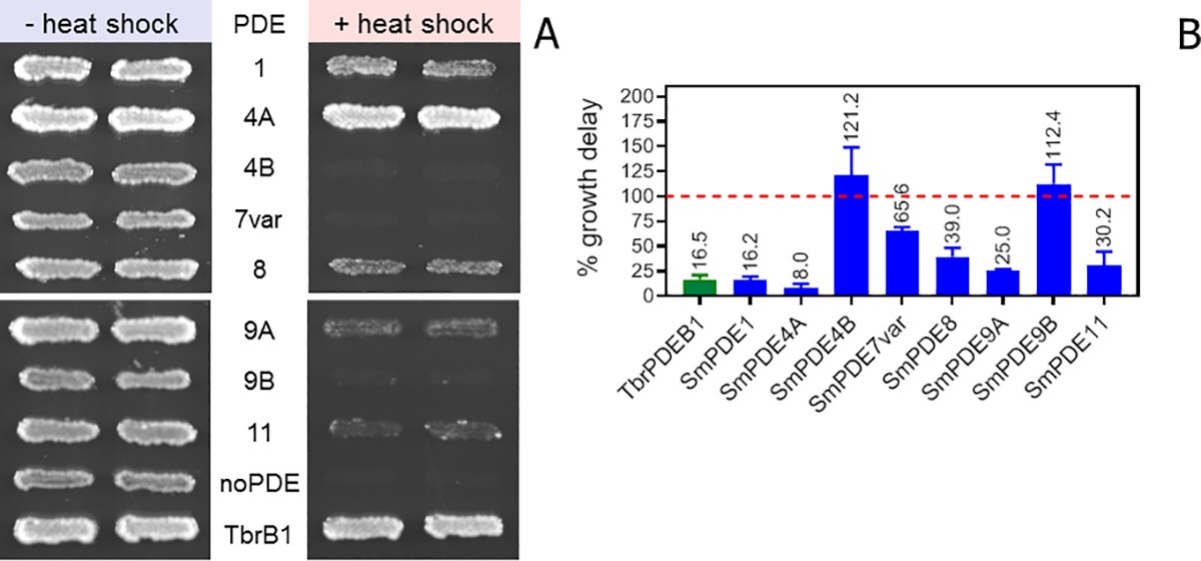
Complementation of temperature sensitivity by SmPDEs. A) Temperature sensitivity of the *pde1/*2 deletion strain PP5 is complemented to various extents by SmPDE1, SmPDE4A, SmPDE8, SmPDE9A and SmPDE11, respectively. PP5 transformed with TbrPDEB1 was taken along as positive control. Duplicate patches of recombinant yeast strains were subjected to heat shock (55°C for 15 min) or control conditions (30 ^o^C) and were then grown at 30°C for 1 day. (B) Heat shock complementation in liquid culture. Growth of *pde1/2* deletions strain (PM943) expressing SmPDEs was analyzed after exposure to an increased temperature (4 min, 52 ^o^C) or in control condition. Growth (OD600) was examined over 72 h. The resulting growth curves were analyzed for onset of the exponential phase. Comparison of the growth curves of cultures exposed to heat shock and control conditions indicate that SmPDE4B are unable to complement the phenotype (a delay in onset of exponential growth compared to the control, set at 100% (red dotted line). In this assay, TbrPDEB1 (positive control (in green)), SmPDE1, SmPDE4A, SmPDE8, SmPDE9A and SmPDE11 and SmPDE7var display a faster onset of the exponential growth after a high temperature challenge compared to the control condition.

As functional complementation in *T*. *brucei* was not immediately successful with the majority of SmPDEs attempted, we also utilized the previously published yeast complementation system [47] in order to assess functionality of the SmPDE genes as phosphodiesterases. Complementation of the temperature sensitivity of the *Saccharomyces cerevisiae* strains PP5 and PM943 (both double deletion mutants for *PDE1* and *PDE2*) was examined. *S*. *cerevisiae* lacking both of its endogenous PDEs are viable but display a marked temperature sensitivity [48, 49] which has successfully been utilized to validate heterologously expressed PDEs, complementing the phenotype (e.g. [50–53]). After cloning the SmPDE genes into an *S*. *cerevisiae* expression vector, we tested complementation of the heat shock-sensitive phenotype of PP5 as described earlier [54]. As is clear from the data in Fig 7A, besides TbrPDEB1 (positive control), SmPDE1, SmPDE4A, SmPDE8, SmPDE9A and SmPDE11 were able to complement the temperature sensitivity of the *pde1/2* deletion strain, indicative of cAMP degrading PDE activity. Complementation by SmPDE4A was anticipated as the purified protein was shown to possess cAMP degrading activity in an earlier study [45] and we show in this study also complementation in *T*. *b*. *brucei*. For the other complementing SmPDE genes, this is the first report highlighting them as potential cAMP degrading enzymes. Expression of SmPDE4B, and SmPDE7var did not complement the temperature sensitive phenotype in this experimental setup (Fig 7A). Comparable results were obtained expressing the SmPDE genes in the PM943 strain, albeit that the complementation by SmPDE1 was less pronounced in this system (*pde1/2* deletion in a different genetic background, S5 Fig).

Next, we analyzed the SmPDE expressing *S*. *cerevisiae* strains (PM943 background) in liquid cultures allowing us to determine complementation of the temperature sensitivity under less stringent conditions (Fig 7B). Decreasing both time of exposure and temperature, to 4 min and 52 ^o^C, respectively, still resulted in a marked temperature sensitivity in the control strain, which was complemented by the same SmPDE constructs that rescued the temperature sensitivity in Fig 7A. Under these conditions SmPDE4B was still unable to complement the phenotype. However, expression of SmPDE7var also mediated a degree of complementation and could therefore be included in the group of cAMP degrading PDEs from *S*. *mansoni*.

In conclusion, SmPDE1, SmPDE4A, SmPDE7var, SmPDE8, SmPDE9A and SmPDE11 display cAMP degrading activity. Complementation of the temperature sensitive phenotype of *S*. *cerevisiae* double *pde1/2* deletion strain was stronger for SmPDE1 and SmPDE4A-expressing strains compared to the SmPDE8, SmPDE9A and SmPDE11-expressing strains, whereas expression of SmPDE7var only weakly complemented the phenotype. No complementation was observed when we expressed SmPDE4B. Differences in complementation between the various PDE expressing strains could be due to a lack of intrinsic catalytic activity, expression level or subcellular localization of the different PDEs in the yeast model. Moreover, some PDEs from *S*. *mansoni* would be expected to be cGMP-hydrolyzing enzymes, which is not detectable in the *S*. *cerevisiae* cell model.

### Expression and characterization of SmPDE4A, SmPDE8, and SmPDE11 in *S. pombe*

The fission yeast *S*. *pombe* has been used as a host organism to express and characterize mammalian cAMP and cGMP-specific PDEs as well as to carry out high throughput screens for inhibitors of these enzymes [55–59]. The SmPDE4A, SmPDE8, and SmPDE11 genes were cloned into an expression vector and integrated into the *S*. *pombe* genome to create strains that stably express these enzymes. SmPDE8 and SmPDE11 had displayed rather weak complementation in the *S*. *cerevisiae* system, and it was appropriate to further verify that cAMP was their main substrate; SmPDE4A as the gene giving the strongest complementation in both the *T*. *brucei* and *S*. *cerevisiae* systems, was included as positive control.

**Fig 8:**
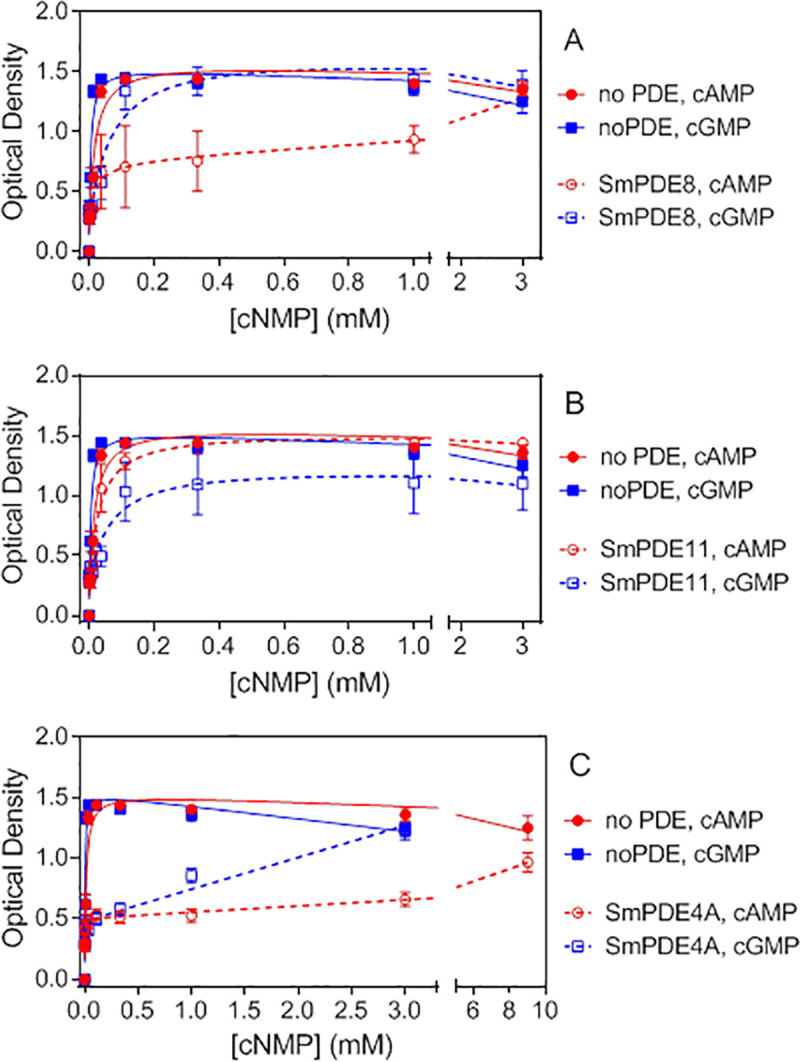
5FOA-resistant growth of *S*. *pombe* strains expressing SmPDE4A, SmPDE8, and SmPDE11 in response to exogenous cAMP or cGMP. 5FOA-resistant growth is due to PKA-mediated repression of an *fbp1-ura4* reporter in these strains. A) Growth response curves to varying doses of cAMP or cGMP in strains expressing SmPDE8 as compared to a strain devoid of PDE activity. B) As frame A, for SmPDE11. C) As frame A, for SmPDE4A. Values represent means and standard errors for three to five independent assays for each strain.

After introduction of the three *S*. *mansoni* genes into *S*. *pombe*, the PDE activity was assessed using a 5-fluoro-orotic acid (5FOA) growth assay, which reflects PKA-mediated transcriptional repression of an *fbp1-ura4* reporter gene. As seen in Fig 8A, cells that lack any PDE activity respond to low micromolar concentrations of cAMP or cGMP to activate PKA and promote growth. A strain expressing SmPDE8 responds to cGMP in a manner similar to that of a strain with no PDE, but requires significantly more cAMP to promote growth, consistent with it possessing a cAMP-hydrolyzing PDE but having almost no activity against cGMP. The SmPDE11-expressing strain shows a greater shift in the amount of cGMP relative to cAMP needed to promote growth when compared to the strain lacking PDE activity, consistent with SmPDE11 being a dual-specificity enzyme that is more active on cGMP than on cAMP (Fig 8B). Finally, the SmPDE4A strain displays the highest PDE activity, such that even 9 mM cAMP is not sufficient to fully activate PKA (Fig 8C). SmPDE4A also appears to confer considerable insensitivity to cGMP to suggest that it is a dual specificity PDE, albeit with a significant preference for cAMP over cGMP. An overview of all SmPDEs and their complementation in the different yeast strains is given as S3 Table.

## Discussion

The shortcomings of praziquantel, including ineffectiveness against immature worms, failure to achieve full clearance, and reports of diminishing efficacy, make the case that alternative treatment options or at a minimum an early drug discovery pipeline for schistosomiasis drugs is essential. Yet, ironically, the virtues of praziquantel—non-toxic, easy to administer and, above all, available and cheap enough for mass-administration programs—make drug discovery programmes for this neglected disease highly unlikely. As argued by Ramamoorthi *et al*. this logically leads towards the repurposing of existing drugs and compound libraries as an economical short-cut towards new treatments of schistosomiasis, as reflected in the fact that almost all recent clinical trials against schistosomiasis have been conducted with existing drugs against other neglected parasitic diseases [60, 61].

We have long argued [62, 63] that highly conserved proteins such as cyclic nucleotide phosphodiesterases (PDEs) potentially make excellent drug targets in neglected diseases because of the shortcuts in development time and money this approach affords, drawing as it does on the wealth of pharmacological, genetic, structural and toxicological information available on PDE inhibitors for the treatment of conditions as diverse as heart failure [64], erectile dysfunction [65] and chronic obstructive pulmonary disorder (COPD) [66]. The exploration of PDEs as potential drug targets for schistosomiasis is in keeping with similar efforts to develop inhibitors for the *Schistosoma* equivalents of targets for cancer [67, 68], hypercholesterolemia [69] or cardiovascular disease [70]. In this strategy the design of the final inhibitors will rely on a combination of high-throughput screening of targeted compound libraries and structure-guided final optimization to achieve the necessary selectivity.

We have recently published a first phenotypic screening of a PDE-inhibitor-enriched library and found that many of the compounds displayed anti-schistosomal effects such as worm survival, ovipositing and movement—with male worms being much more affected than female worms [34]. Several compounds were tested in mouse models of schistosomiasis and displayed partial clearance of worm and egg burdens. Similarly, Conor Caffrey’s group screened a library of benzoxaboroles for antischistosomal activity and found that many of the most active compounds were submicromolar inhibitors of human PDE4B2 [33]. They went on to identify by BLAST-P searches four *S*. *mansoni* PDE sequences: SmPDE4A-C (confirmed in the current manuscript) and SmPDE4D, which we here propose to rename SmPDE8 based on its phylogenetic alignment with hPDE8B, and concluded that SmPDE4A is likely to mediate the effects of the benzoxaboroles under study, in part via expression of SmPDE4A in pde4-deficient *C*. *elegans*. Further inhibitors of SmPDE4A were recently identified by *in silico* screening of PDE inhibitors against a homology model of SmPDE4A [45] and the PDE4 inhibitor roflumilast was investigated for activity against *S*. *mansoni* in vitro and in a mouse model [71].

In this paper we report the first cloning, from cDNA, of 10 of the 11 PDE ORFs that could be identified in the *S*. *mansoni* genome. These 10 sequences appear to be full-length and potentially functional phosphodiesterases with intact catalytic domains. Phylogenetic analysis assigned 8/10 closely to human clades, facilitating the *S*. *mansoni* PDE nomenclature. In the two cases where there was no unambiguous assignment to a hPDE clade, a protein BLAST identified hPDE7 and hPDE11, respectively, as the top hits, and the genes were named SmPDE7var and SmPDE11 accordingly. The *S*. *mansoni* strain from which we derived the cDNA was heterozygous for SmPDE4B, SmPDE7var, SmPDE9B and SmPDE11, and homozygous for the other 6 ORFs. More importantly, we found significant differences such as exon extensions, extra exons and insertions in several of the cloned ORFs. The sequences given in the Long *et al*. [33] paper, being the predicted database sequences of the time, appear to be incorrect or incomplete, although it is possible the differences are attributable to strain differences. Among the significant differences with the published genome (Puerto Rico strain) we observed that our experimental transcript of SmPDE8 was 270 aa shorter than the WBPS14 entry, and had an extended exon 7, replacing exon 8 of Smp_044060. Multiple significant differences were also observed between our sequence of SmPDE9B and the reference entry Smp_342020. Most of the other SmPDEs here reported displayed smaller differences with the reference entries.We found no evidence that the significant differences in SmPDE8 and 9C could be splice variants, as the sequences were separately amplified from cDNA from schistosomula, juvenile, adult, male and female worms, always resulting in products of very similar length.

In order to assess which of these sequences encode for genuine 3’,5’-cyclic nucleotide phosphodiesterases, a cellular complementation system was constructed in a *T*. *brucei* pdeB1^+/-^/pdeB2^+/-^ clonal line, where the SmPDE was expressed conditionally under control of a tetracycline-inducible promotor. Successful complementation in this system enabled the deletion of the second TbrPDEB1/B2 allele while retaining normal growth; upon withdrawal of tetracycline from the medium the cells would die. This system does not only unambiguously demonstrate cAMP catalytic activity of the heterologous PDE, it is ideal for the screening of inhibitors against the expressed PDE in a cellular system that is easily grown in any reasonable quantity and highly amenable to high-throughput screens in suspension cultures in multi-well plate formats. SmPDE4A, expressed in this system, complemented very well for the deletion of the TbrPDEB1/B2 alleles, and was subjected to an initial proof-of-concept screen with a limited number of PDE inhibitors, performed in parallel with cell lines expressing TbrPDEB1 or TbrPDEB2 only under the same tetracycline-dependent promotor. The strongest inhibitor identified from this limited series was NPD-356, which displayed an EC_50_ of just 5.2 ± 1.2 nM against the SmPDE4A-expressing cell line, significantly lower than the corresponding EC_50_ values of the clones expressing TbrPDEB1 or TbrPDEB2. Other inhibitors with significant preference for the *Schistosoma* PDE over *T*. *brucei* PDEs included NPD-001 and NPD-226; all three were shown to induce elevated intracellular cAMP levels in *T*. *brucei*. Thus, this complementation approach allows the parallel screening of multiple cell lines, each dependent on (and different by only) a single PDE, allowing rapid comparison of relative potency for each inhibitor, as well as immediate verification of on-target action in a eukaryotic cell through the measurement of intracellular cAMP. However, the complementation of some SmPDEs in this system was not immediately successful, owing to the requirement that a significant cAMP-PDE activity be present in the flagellum. Although we engineered the 70 N-terminal amino acids of TbrPDE-B1 onto the complementation constructs of all SmPDEs, which should have ensured this [42], it is possible that the tagging with GFP prevented the correct localization. Meanwhile, further complementation, with the principal aim of demonstrating catalytic activity rather than inhibitor screening, progressed via expression in the budding yeast *Saccharomyces cerevisiae* and the fission yeast *Schizosaccharomyces pombe*.

Utilizing the temperature sensitivity of *S*. *cerevisiae pde1/2* double deletion strains, we have been able to confirm cAMP hydrolyzing activity in several of the SmPDEs heterologously expressed in this cell system. Particularly robust complementation was observed when expressing SmPDE1 and SmPDE4A, which may allow the further biochemical and pharmacological characterization of these PDEs in yeast extract, as has recently been done for *Giardia lamblia* PDE1 [53]. Alternatively, expression in yeast, bacteria or insect cells, and subsequent isolation and purification may be feasible to further characterize the purified enzymes biochemically (enzyme kinetics and substrate specificity) and pharmacologically. Specific pharmacological profiles of the SmPDEs may allow determination of the PDE(s) that can be therapeutically targeted to prevent or cure schistosomiasis. Evidence of more modest cAMP PDE activity was also observed with SmPDE8, SmPDE9A and SmPDE11, whereas SmPDE4B was inactive in this system.

Both the *T*. *brucei* and *S*. *cerevisiae* complementation systems only allow complementation by cAMP phosphodiesterases but cannot function with cGMP-specifc PDEs; however the *S*. *pombe* system is able to detect both cAMP and cGMP degradation by the heterologously expressed enzyme and we used it to demonstrate PDE activity of SmPDE4A, SmPDE8 and SmPDE11. Using a 5FOA growth assay that reflects PKA activity, strains expressing these three enzymes (while lacking endogenous adenylyl cyclase and PDE activity) were shown to require more exogenous cAMP and/or cGMP to activate PKA and promote 5FOA-resistant growth than is required for a strain devoid of PDE activity. Similar to mammalian PDE8A and PDE11A when expressed in this system [56, 57], SmPDE8 selectively acts on cAMP, while SmPDE11 shows a preference for cGMP over cAMP. In addition, SmPDE4A is significantly more active than either SmPDE8 or SmPDE11, similar to what was seen for mammalian PDE4 enzymes relative to PDEs from 9 other PDE families [57, 58]. However, SmPDE4A appears to display a considerable activity against cGMP in addition to a substantially higher activity against cAMP.

In conclusion, we report here the naming and cataloguing of 11 predicted *Schistosoma mansoni* PDEs and the cloning of 10 of them. Confirmation of cyclic nucleotide degradation was obtained for SmPDE1, SmPDE4A, SmPDE8, SmPDE9A and SmPDE11, and to a lesser extent SmPDE7var. SmPDE11was mostly specific for cGMP and SmPDE4A displayed considerable dual activity. An innovative complementation system in *T*. *brucei* was shown to allow rapid and comparative inhibitor screening of multiple PDEs in a standardized cellular system. We believe this is an important contribution in developing a new drug discovery pipeline for schistosomiasis based on targeting conserved, druggable elements in its regulatory systems.

## Materials and methods

### Identification of potential SmPDE genes

The *S*. *mansoni* genome (ftp 1) and protein (ftp 2) sequence databases provided by WormBase/ParaSite (WBPS14) [72. 73] were searched for the presence of class I PDEs using the HMMER software package (version 3.1b2) [74]. The profile hidden Markov model (HMM) was generated from a curated multiple sequence alignment of 24 catalytic domain sequences comprising one member of all 11 human PDE families as well as characterized PDEs from protozoan parasites, *Drosophila melanogaster*, *Caenorhabditis elegans* and *Saccharomyces cerevisiae* as described earlier [53]. The searches against the genome database for detecting PDE sequences not present in the current/existing gene models were done using two different approaches: (1) The genome sequence was split into fragments of 1500 bases that overlap by 750 bases and all fragments were translated into amino acid sequences (all 6 reading frames); (2) all open reading frames with a length of at least 90 nucleotides were translated into amino acid sequences. The thus generated datasets were searched using the PDE profile HMM. Search parameters are given in S3 File. Hit sequences with an E-value between 0.1 and 10 were manually inspected.

ftp 1: ftp://ftp.ebi.ac.uk/pub/databases/wormbase/parasite/releases/WBPS14/species/schistosoma_mansoni/PRJEA36577/schistosoma_mansoni.PRJEA36577.WBPS14.genomic.fa.gz

ftp 2:

ftp://ftp.ebi.ac.uk/pub/databases/wormbase/parasite/releases/WBPS14/species/schistosoma_mansoni/PRJEA36577/schistosoma_mansoni.PRJEA36577.WBPS14.protein.fa.gz

### Parasite strains

Schistosomula, juvenile and adult *Schistosoma mansoni* worms recovered 3, 4 and 6 weeks post infection of mice with 70 *S*. *mansoni* cercariae were obtained from the Schistosome Biology Supply Center (SBSC) of the Theodor Bilharz Research Institute (TBRI), stabilized using RNAlater (Invitrogen, ThermoFisher) and sent for processing from TBRI to the University of Glasgow. RNA was extracted from worms and schistosomula using a Macherey Nagel Nucleospin RNA extraction kit, and from this, cDNA was produced using Superscript III reverse transcriptase (ThermoFisher), all following manufacturer’s instructions. Genomic DNA was prepared from isolated worms at TBRI and shipped to University of Glasgow.

Bloodstream-form *T*. *b*. *brucei* Lister 427 2T1 strain [75] and its derivatives were maintained in HMI-11 medium [76], at 37°C in a 5% CO_2_ atmosphere, exactly as described [77].

### Amplification of PDEs

Depending on the arrangement of the exons, the first 1–3 and last 1–2 exons of all 11 *S*. *mansoni* PDEs were amplified from genomic DNA, using primers upstream or downstream of the predicted exons and a primer in the predicted exon (see S4 Table for primers and details), and a proof reading polymerase (Phusion; NEB, Hitchen, UK). This sequence information was used to design primers to amplify the complete coding sequence for each PDE from mixed adult worm cDNA, again using Phusion polymerase. Some PDEs were amplified in two sections due to size/primer incompatibility (S4 Table). These strategies were successful for all predicted SmPDE ORFs except SmPDE2. All sequences generated were submitted to GenBank (Table 1). cDNA produced from different *S*. *mansoni* life-cycle stages (schistosomula; juvenile female/male worms; adult female/male worms) were used in attempts to amplify the complete SmPDE2 or sections thereof, alongside attempts to amplify individual exons from genomic DNA. All amplified fragments were ligated into the pGEMT-Easy subcloning vector (Promega, Southampton, UK) and Sanger sequenced by Source BioScience (Nottingham, UK). It should be noted that a recently launched online resource schisto.xyz [37] lists SmPDE2 as detectable by RNAseq, and expressed particularly in adult worms (SmPDE2).

### Phylogenetic analysis

The sequences of the SmPDEs were compared to representative members of the eleven human PDE families; the sequences of these were taken from the UniProt database [78], taking the top human hit for each PDE number (http://www.uniprot.org/; hPDE1C - Q14123; hPDE2A3—O00408; hPDE3A - Q14432; hPDE4B1—Q07343; hPDE5A1—O76074; hPDE6A - P16499; hPDE7B - Q9NP56; hPDE8B - O95263; hPDE9A - O76083; hPDE10A - Q9Y233; hPDE11A - Q9HCR9). The in-house sequences for SmPDEs were used where available while of necessity using the GeneDB sequences for SmPDE2. In order to derive a systematic naming convention a phylogenetic tree was constructed, using the Maximum Likelihood Tree function of MEGA6, with 500 bootstrap replications and the default parameters ([79]; https://www.megasoftware.net/). This was complemented with BLAST searches using the SmPDE protein sequences to compare naming of orthologues in *S*. *haematobium* found using the WormBase Parasite database ([73]; https://parasite.wormbase.org/Schistosoma_haematobium_prjna78265/Info/Index?db=core).

### qRT-PCR expression profiling

The expression profile of each of the 9 amplified *S*. *mansoni* PDEs was generated from five separate cDNA pools: schistosomula, juvenile female and male worms, adult female and male worms. Primers used are given in S4 Table. GoTaq qPCR master mix (Promega) was used to amplify the fragments, using an Applied Biosystems 7500 Real Time PCR System, following manufacturer’s instructions. The differences in expression levels were calculated relative to the *S*. *mansoni* COX-1 gene as control (primers from [80]), previously shown to be highly and constitutively expressed in various lifecycle stages [81].

### Trypanosome plasmid construction and transfection

Each amplified SmPDE was resynthesized by BaseClear (Leiden, The Netherlands) with *Trypanosoma brucei*-optimized codon usage. The codon optimization was completed using the Integrated DNA Technologies tool (https://www.idtdna.com/CodonOpt) with manual checking to remove unwanted restriction sites from the sequences.

For expression in *T*. *brucei* the genes were inserted into the tetracycline-inducible pRPa-series of vectors [41] for expression from a tagged rRNA locus. TbrPDEB1 and TbrPDEB2 were inserted into the pRPa^ix6Myc^ vector containing a C-terminal 6Myc tag, whilst the SmPDEs were inserted into the pRPa^ixGFP^ vector, giving proteins with C-terminal Green Fluorescent Protein (GFP) tags. In addition, the first 210 bp of TbrPDEB1 were amplified from *T*. *brucei* Lister 427 genomic DNA and added to the N-terminal end of all SmPDE genes to drive flagellar localization. This region has been previously shown to be necessary and sufficient for the targeting of TbrPDEB1 to its essential flagellar location [42].

For the knockout of the TbrPDEB1-B2 locus, constructs were generated using the cre-lox null vectors developed by George Cross [40], which contain an antibiotic resistance gene fused to the Herpes Simplex Virus thymidine kinase (HSV-TK) gene, flanked by loxP sites to allow removal of the resistance cassette by cre-recombinase. The HSV-TK gene allows for selection of cells in which the cre-lox process has successfully removed the antibiotic cassette, as the cells regain resistance to ganciclovir when the HSV-TK gene is removed. To integrate the antibiotic-resistance cassettes, a 321 bp region of the 5’ UTR of TbrPDEB1 and a 560 bp region of the 3’ UTR of TbrPDEB2 was introduced on either side of the antibiotic-resistance gene in order to simultaneously knock out the in-tandem TbrPDEB1 and TbrPDEB2 ORFs. Constructs containing blasticidin-HSV-TK and puromycin-HSV-TK resistance cassettes were used to sequentially knock out the two tandemly arranged alleles.

All plasmids were checked by Sanger Sequencing (Source BioScience) and linearized by restriction digest prior to transfection. 2T1 strain of Lister 427 *T*. *brucei* parasites were washed into Tb-BSF buffer for transfection of the desired cassette with program X-001 using an Amaxa Nucleofector as described [82]. Transfectants were grown and cloned out, by limiting dilution, in standard HMI‐11 medium containing the appropriate antibiotics (depending on the transfection: hygromycin at 2 μg/ml, blasticidin at 5 μg/ml, puromycin at 0.5 μg/ml, phleomycin at 0.5 μg/ml and tetracycline at 1 μg/ml). Correct integration of the expression/knockout cassettes was verified by PCR (primers in S4 Table). A complete list of *T*. *brucei* vectors used is given in S1 Table).

### Immunofluorescence

Fluorescent imaging of tagged cell lines was performed as previously described [83] with slight modifications. Briefly, the different cell lines were cultured to a concentration of 1×10^6^ cells/ml and 1 ml was taken and centrifuged at 1000 × *g* in a microfuge. The media was removed and pellet washed with phosphate-buffered saline (PBS); excess PBS was taken off, pellets resuspended in 20 μl PBS and spread on microscopic slides. The slides were air-dried, fixed in 4% formaldehyde/PBS for 15 min and rinsed in PBS three times before the slides were treated with 0.1% Triton X-100 (Sigma-Aldrich) in PBS for 5 min with gentle shaking in order to permeabilize the cells. Blocking of non-specific binding was performed in 1% bovine serum albumin (BSA)/PBS for 30 min after which the slides were incubated with primary antibody (Anti-GFP antibody) diluted 1:2000 in 1% BSA/PBS for 1 h at room temperature (RT) on a shaker (speed 12–15). After incubation with primary antibody, slides were washed thrice with PBS for 10 min and then incubated with secondary antibody (Alexa Fluor 594 goat anti-rabbit IgG (H+L); Life Technologies) diluted 1:1500 in 1% BSA/PBS at RT for 45 min. The slides were rinsed three times in PBS for another 10 min, soaked in 70% ethanol for 1 min followed by 100% ethanol for 1 min, all at RT. Finally, the slides were air-dried, rehydrated in PBS and were mounted in a drop of Vectashield Mounting medium containing 4,6-diamidino-2-phenylindole (DAPI; Vector Laboratories Inc.). Cells were visualized using oil immersion at 100× magnification under UV light using a Delta Vision Core microscope (Applied Precision) and SoftWorkX software. Cells were viewed using Brightfield, DAPI (λ = 405/435 nm) and FITC (λ = 488/520 nm) filters.

### Trypanosome drug sensitivity assays

Drug sensitivities were assessed using a modified version of the alamar blue assay described previously [84, 85]. Briefly, test drugs were serially diluted in 100 μl of complete HMI‐11 media across two rows of a white 96-well plate (Greiner, Stonehouse, UK). Bloodstream‐form trypanosome cultures were diluted in HMI‐11, and 100 μl was added to all wells to give a final cell density of 1 × 10^5^ trypanosomes/ml. Plates were incubated for 48 h at 37°C/5% CO_2_, prior to the addition of 20 μl of 5 mM resazurin sodium salt (Sigma‐Aldrich, Gillingham, UK) in PBS, pH7.4. Plates were incubated for a further 24 h in the same conditions, before fluorescence was measured using a FLUOstar Optima fluorimeter (BMG Labtech, Aylesbury, UK) with excitation and emission filters of 544 nm and 590 nm, respectively. The 50% effective concentrations (EC_50_) were calculated using the equation for a sigmoidal curve with variable slope of Prism 5.0 (GraphPad). All EC_50_ values were determined at least three times independently.

### Potential PDE inhibitors

All of the potential inhibitors tested on SmPDE4A-expressing cell lines were prepared at the Free University of Amsterdam (VUA; Amsterdam The Netherlands), University of Antwerp (UA; Antwerp, Belgium) and Centro de Investigaciones Biológicas (CIB-CSIC; Madrid, Spain) and have a purity ≥95% by HPLC. A list including structures and references is included as S5 Table.

### Quantification of intra- and extra-cellular cAMP

The intra- and extra-cellular concentration of cAMP in bloodstream form *T*. *brucei brucei* cell lines, upon incubation with various phosphodiesterase inhibitors, was measured as described previously [86], using the Cyclic AMP ELISA kit (Cayman Chemicals, Cambridge Bioscience, Cambridge, UK). Samples were taken in duplicate, and all assays were conducted independently at least three times.

### Yeast strains, plasmid construction and transformation

The SmPDE gene sequences were cloned into a yeast (*S*. *cerevisiae*) expression vector (pJB341; YCplac111-based, sc, LEU2, PGK constitutive promoter cloned into the EcoRI and BamHI sites) using the appropriate restriction sites. Cloning was done directly from the *T*. *brucei-*optimized DNA sequences in pUC57-Amp (Baseclear, Leiden, NL) or after re-PCR from the synthesized genes and cloning in an intermediate vector. SmPDE4A_FL was amplified with 5’ BamHI and 3’ SphI sites and cloned into intermediate vector pSSS05 (pOPINF). SmPDE4B_FL was amplified with 5’ BamHI and 3’ XbaI sites and cloned into intermediate vector pSSS013 (pFastBac HT B). SmPDE9A_FL was amplified with 5’ BamHI and 3’ SphI sites and cloned into intermediate vector pSSS014 (pFastBac HT B). SmPDE9B_FL was amplified with 5’ BamHI and 3’ XhoI sites and cloned into intermediate vector pSSS011 (pFastBac HT B). SmPDE1_FL was amplified with 5’ BamHI and 3’ SphI sites, cloned into intermediate vector pSSS012 (pFastBac HT B). Both SmPDE8_FL and SmPDE11 were cloned directly using the 5’ BglII -SphI 3’ sites in the synthesized gene. Transformation of the constructs into the PDE-deficient *S*. *cerevisiae* strains PP5 (*MAT*a *leu2*,*112 ura3-52 his3-532 his4 cam pde1*::*URA3 pde2*::*HIS3*) [51] and PM943 (W303-1A *pde1*Δ *pde2*Δ; *MAT*a *leu2-3*,*112 ura3-1 trp1-92 his3-11*, *15 ade2-1 can1-100*, *GAL SUC mal pde1*::*TRP1 pde2*::*URA3*) [49] was carried out as described previously [47].

### Complementation of the heat shock-sensitive *S. cerevisiae* pde1/2 double deletion strain

The heat shock-sensitive phenotype of the PDE-deficient *S*. *cerevisiae* strains PP5 and PM943 was utilized to analyze the functionality of heterologously expressed SmPDEs. These assays were carried out as described [47]. Transformed yeast strains were streaked onto SC-leu plates (selective medium lacking leucine) and grown for 2 days at 30°C. The colonies were then replica-plated onto YPD plates prewarmed to 55°C, and were incubated for another 15 min at 55°C. Plates were then cooled to room temperature and incubation was continued at 30°C for 18–36 h. Alternatively, complementation of the heat shock-sensitive phenotype of the *pde1/2* deletion strain (PM943) was measured in liquid cultures, for which a fresh colony was used to inoculate 5 ml of SC-leu medium. The culture was grown for 54 h at 30 ^o^C while shaking (190 rpm) and then diluted to OD600 1.0 in SC-leu medium (500 μl volume). Heat shock was performed in Eppendorf tubes; 150 μl culture was exposed to 52 ^o^C for 4 min (in water bath) and subsequently cooled on ice (1.5 min). Heat-shocked and control cultures were diluted 1:10 in YPD and analysis of growth (OD600) was made in 96 wells plates (clear, flat bottom; Greiner) in a plate reader (Infinite F200, Tecan) pre-heated to 30 ^o^C. Data was captured in the Magellan software (Tecan) and analyzed using Excel (MS Office 2016) and Prism 8.0 software (GraphPad). Complementation was determined by comparing the time to reach early-exponential phase in control conditions and after exposure to increased temperature.

### Construction of *Schizosaccharomyces pombe* strains expressing SmPDE4A, SmPDE8, and SmPDE11

The genotypes of *S*. *pombe* strains used in this study are presented in S6 Table. Cells were cultured at 30 ºC in YES-rich (yeast extract medium with supplements) or EMM-defined media as described previously [87]. SmPDE4A, SmPDE8, and SmPDE11 were cloned into the *S*. *pombe* expression vector pNMT1 [88] by PCR amplification of the open reading frames using custom oligonucleotides (S7 Table) designed to facilitate insertion of the genes into *Eco*RI-digested pNMT1 by gap repair transformation of strain SP578 to leucine prototrophy [89, 90]. SP578 carries a loss-of-function mutation in the *S*. *pombe cgs2* PDE gene and is a homothallic (*h*^90^) strain, such that cells within a single colony are capable of mating with each other. However, the *cgs2-2* mutation eliminates endogenous PDE activity and the elevated cAMP levels activate PKA to prevent cells from mating within the colony. Transformant colonies were screened by iodine vapor staining in order to detect colonies that acquired an active PDE as this would reduce cAMP levels to restore mating. Plasmids from these colonies were rescued into *E*. *coli* [91] and DNA sequencing was used to identify clones carrying wild type alleles of these PDE genes. These plasmids were then linearized by *Blp*I digestion, which cuts uniquely within the *ars1* region of pNMT1 and *S*. *pombe* strain CHP1265 was transformed to leucine prototrophy, followed by screening via plasmid loss to detect transformants carrying the plasmids integrated into the *ars1* chromosomal locus. Subsequent crosses confirmed that these plasmids were integrated, and they were used to construct strains lacking endogenous adenylyl cyclase and PDE activity to allow for characterization of SmPDE4A, SmPDE8, and SmPDE11 with regard to catalytic activity against cAMP and cGMP.

### S. pombe 5FOA growth assays to examine substrate specificity of SmPDE4A, SmPDE8, and SmPDE11

Strains CHP1207 (no PDE), CHP2344 (SmPDE8) and CHP2346 (SmPDE11) were grown to exponential phase (~10^7^ cells/ml) in EMM medium containing 10 mM cAMP to repress transcription of the *fbp1-ura4* reporter used to reflect PKA activity. As PDE4A possesses a high level of activity against cAMP, strain CHP2345 (PDE4A) was grown to exponential phase in EMM medium containing 5 mM cGMP and 5 μg/ml thiamine (to reduce transcription of PDE4A from the *nmt1* promoter [92]). Cells were washed with 5FOA medium [93] to remove exogenous cAMP or cGMP and plated as 50 μl cultures at either 10^5^ cells/ml (CHP1207) or 3×10^5^ cells/ml (CHP2344, CHP2345, CHP2346) in 384-well clear-bottom plates with various concentrations of either cAMP or cGMP. Triplicate wells were averaged for each data point and OD_600_ was measured after 48 h incubation at 30 ºC. Each strain was assayed 3–5 times.

## Supporting information

S1 Fig Sequence alignments of all cloned SmPDEs with genome entries. (PDF)

S2 Fig PCR of SmPDE9C in multiple *S*. *mansoni* lifecycle stages. (PDF)

S3 Fig Matrix of amino acid percentage sequence identity for all SmPDEs and examplar hPDEs and kinetoplastid PDEs. (PDF)

S4 Fig PCR analysis of the SmPDE4A complementation in *T*. *b*. *brucei*. (PDF)

S5 Fig Complementation of SmPDEs in yeast strain PM943. (PDF)

S1 Table List of plasmids used for knockouts and expression in *T*. *brucei*. (PDF)

S2 Table Antischistosomal effects of PDE inhibitors. (PDF)

S3 Table Ability of SMPDEs to commplement in the two yeast systems. (PDF)

S4 Table Primers used for the work on *T*. *brucei* strains and constructs. (PDF)

S5 Table Structures of the PDE inhibitors used. (XLSX)

S6 Table Genotypes of the *S*. *pombe* strains used. (PDF)

S7 Table Oligonucleotides used in the construction of *S*. *pombe* strains. (PDF)

S1 File FASTA exon nucleotide sequence of SmPDEs. (PDF)

S2 File FASTA amino acid sequence of SmPDEs. (PDF)

S3 File HMM searches for SmPDEs. (XLSX)
