# Supplementary material for: Cloning and functional complementation of ten Schistosoma mansoni phosphodiesterases expressed in the mammalian host stages
Source: PLoS Negl Trop Dis. 2020 Jul 30;14(7):e0008447. doi: 10.1371/journal.pntd.0008447 (PMC7430754; doi:10.1371/journal.pntd.0008447)
Supplement: S1 Fig — (PDF) [file pntd.0008447.s001.pdf]

S1 Fig. Sequence alignments of all cloned SmPDEs with the variants in the WormBase/ParaSite database (WBPS14)

Legend

Sequence labels

- SmPDE isoforms of the Egyptian CD strain described in this study
  are labeled with the PDE name and appended allele (a) or splice variant (sv) identifier (e.g. SmPDE4B-a1)
- Variants annotated in the WormBase/ParaSite database (WBPS14)
  are denoted with their database identifiers (e.g. Smp_134500.1)

Sequence differences

● / ○ : Amino acid substitutions
closed circles: homozygous substitutions between the lab strain (Egyptian CD) and the genome reference strain (Puerto Rican); open circles: heterozygous substitutions between isoforms of the Egyptian CD strain

▲ / Δ : 1-amino acid inserts/deletions (indels)
closed triangles: differences between the lab strain (Egyptian CD) and the genome reference strain (Puerto Rican), open triangles: differences between isoforms of the Egyptian CD strain

~~~~ : Insert in the catalytic domain of SmPDE9C potentially affecting the enzyme function

– – – : Deletion in or truncation of the catalytic domain putatively affecting the enzyme function

★ : Alternative splicing

D S : Amino acid residues at the C-terminal end of the SmPDE4B database variant Smp_141980.2 that diverge from conserved residues in class I PDEs

Catalytic domain

▬ : Predicted position of the catalytic domain
The catalytic domain boundaries were deduced from crystal structure data of human orthologues combined with secondary structure predictions.

Exons

An exon Another exon : The deduced amino acid sequence of every second exon is highlighted in blue

A VARIANT : exon variants
A VARIANT are highlighted in
A VARIANT brown, violet or grey

exon 1 (5.e1): Exon numbers and identifiers in brackets. The identifiers are abbreviated as exemplified for two PDE4A exons:
- **5.e1** denotes the WBPS14 database entry Smp_134140.**5.e1**
- **4A.e1** is exon SmPDE**4A.e1** as listed in “S1 File. Protein coding exons”
~~~~

SmPDE1

```
Smp_134500.1   MGSCASTGVSQAGSPAKSQLTNGIQQTSFNIQEMNQTEIRNINEEGDAMAGQEKKTDNPRSNYVLSGIDL  70
Smp_134500.2   MGSCASTGVSQAGSPAKSQLTNGIQQTSFNIQEMNQTEIRNINEEGDAMAGQEKKTDNPRSNYVLSGIDL  70
SmPDE1-sv1     MGSCASTGVSQAGSPAKSQLTNGIQQTSFNIQEMNQTEIRNINEEGDAMAGQEKKTDNPRSNYVLSGIDL  70
SmPDE1-sv2     MGSCASTGVSQAGSPAKSQLTNGIQQTSFNIQEMNQTEIRNINEEGDAMAGQEKKTDNPRSNYVLSGIDL  70
               **********************************************************************
encoded by:                            exon 1 (1.e3)

Smp_134500.1   LDYGVGLKDIPLRTSDTNMTTDEELDMECALSGVSQFFYKNLTTCTLGQYAQIPNDTDMLRSVNTPESLR  140
Smp_134500.2   LDYGVGLKDIPLRTSDTNMTTDEELDMECALSGVSQFFYKNLTTCTLGQYAQIPNDTDMLRSVNTPESLR  140
SmPDE1-sv1     LDYGVGLKDIPLRTSDTNMTTDEELDMECALSGVSQFFYKNLTTCTLGQYAQIPNDTDMLRSVNTPESLR  140
SmPDE1-sv2     LDYGVGLKDIPLRTSDTNMTTDEELDMECALSGVSQFFYKNLTTCTLGQYAQIPNDTDMLRSVNTPESLR  140
               **********************************************************************
                                              exon 2 (1.e4)

Smp_134500.1   ACYVRMRHIYRMIETDRMGKATLMKNIQYAINVMENAYIAEKRRIREEEEDLSEAATEYVPDEVRNWLAS  210
Smp_134500.2   ACYVRMRHIYRMIETDRMGKATLMKNIQYAINVMENAYIAEKRRIREEEEDLSEAATEYVPDEVRNWLAS  210
SmPDE1-sv1     ACYVRMRHIYRMIETDRMGKATLMKNIQYAINVMENAYIAEKRRIREEEEDLSEAATEYVPDEVRNWLAS  210
SmPDE1-sv2     ACYVRMRHIYRMIETDRMGKATLMKNIQYAINVMENAYIAEKRRIREEEEDLSEAATEYVPDEVRNWLAS  210
               **********************************************************************
                                exon 3 (1.e5)                    exon 4 (1.e6)

Smp_134500.1   TFTRTVQSVGIGDQKPRFRSVANAIRAGIFVERIYRRMSSCSNLIVPPNVLLFLKTGLDTWNFDVFGLNE  280
Smp_134500.2   TFTRTVQSVGIGDQKPRFRSVANAIRAGIFVERIYRRMSSCSNLIVPPNVLLFLKTGLDTWNFDVFGLNE  280
SmPDE1-sv1     TFTRTVQSVGIGDQKPRFRSVANAIRAGIFVERIYRRMSSCSNLIVPPNVLLFLKTGLDTWNFDVFGLNE  280
SmPDE1-sv2     TFTRTVQSVGIGDQKPRFRSVANAIRAGIFVERIYRRMSSCSNLIVPPNVLLFLKTGLDTWNFDVFGLNE  280
               **********************************************************************
                                                    exon 5 (1.e7)

Smp_134500.1   ASENHALKFVAFELLHKYNLINKFQINSTALESLLIQLETGYSKYSNPYHNLVHAADVMQTCHMIIFMND  350
Smp_134500.2   ASENHALKFVAFELLHKYNLINKFQINSTALESLLIQLETGYSKYSNPYHNLVHAADVMQTCHMIIFMND  350
SmPDE1-sv1     ASENHALKFVAFELLHKYNLINKFQINSTALESLLIQLETGYSKYSNPYHNLVHAADVMQTCHMIIFMND  350
SmPDE1-sv2     ASENHALKFVAFELLHKYNLINKFQINSTALESLLIQLETGYSKYSNPYHNLVHAADVMQTCHMIIFMND  350
               **********************************************************************
                     exon 6 (1.e8)                   exon 7 (1.e9)
                 Δ
Smp_134500.1   LRQNWLNDLDIFAVLFAAVIHDYEHTGTTNNFHIATRSELALIYNDRGVLENHHVSAVFRLMQEEEFSIL  420
Smp_134500.2   LR-NWLNDLDIFAVLFAAVIHDYEHTGTTNNFHIATRSELALIYNDRGVLENHHVSAVFRLMQEEEFSIL  419
SmPDE1-sv1     LR-NWLNDLDIFAVLFAAVIHDYEHTGTTNNFHIATRSELALIYNDRGVLENHHVSAVFRLMQEEEFSIL  419
SmPDE1-sv2     LRQNWLNDLDIFAVLFAAVIHDYEHTGTTNNFHIATRSELALIYNDRGVLENHHVSAVFRLMQEEEFSIL  420
               ** *******************************************************************
                       exon 8 (1.e10/2.e10)                exon 9 (1.e11)

Smp_134500.1   TGLEADQYKEFRQLVIDMVLCTDMSLHFQQIKNMKTMISMPESIDKTKALSLIVHCADISHPAKEWALHE  490
Smp_134500.2   TGLEADQYKEFRQLVIDMVLCTDMSLHFQQIKNMKTMISMPESIDKTKALSLIVHCADISHPAKEWALHE  489
SmPDE1-sv1     TGLEADQYKEFRQLVIDMVLCTDMSLHFQQIKNMKTMISMPESIDKTKALSLIVHCADISHPAKEWALHE  489
SmPDE1-sv2     TGLEADQYKEFRQLVIDMVLCTDMSLHFQQIKNMKTMISMPESIDKTKALSLIVHCADISHPAKEWALHE  490
               **********************************************************************
                                 exon 10 (1.e12)                 exon 11 (1.e13)

Smp_134500.1   QWSDILCEEFFRQGDRERELNLPISPLCDRNTVVVPQSQIGFIDFIVEPSFQVLGDMIERIVNPTQTEGV  560
Smp_134500.2   QWSDILCEEFFRQGDRERELNLPISPLCDRNTVVVPQSQIGFIDFIVEPSFQVLGDMIERIVNPTQTEGV  559
SmPDE1-sv1     QWSDILCEEFFRQGDRERELNLPISPLCDRNTVVVPQSQIGFIDFIVEPSFQVLGDMIERIVNPTQTEGV  559
SmPDE1-sv2     QWSDILCEEFFRQGDRERELNLPISPLCDRNTVVVPQSQIGFIDFIVEPSFQVLGDMIERIVNPTQTEGV  560
               **********************************************************************
                                 exon 12 (1.e14)                exon 13 (1.e15)

Smp_134500.1   LPTDTTSPKPKSSDQETVGEQVVPRPWVEHFKENKESWSKKLPPKT  606
Smp_134500.2   LPTDTTSPKPKSSDQETVGEQVVPRPWVEHFKENKESWSKKLPPKT  605
SmPDE1-sv1     LPTDTTSPKPKSSDQETVGEQVVPRPWVEHFKENKESWSKKLPPKT  605
SmPDE1-sv2     LPTDTTSPKPKSSDQETVGEQVVPRPWVEHFKENKESWSKKLPPKT  606
               *****************     .     :  :
                                      exon 14 (1.e16)
```

SmPDE4A

```
Smp_134140.4    ---------------------------------------------------------------------------  0
Smp_134140.1    --------------------------------------------------------------------------M  1
Smp_134140.2    --------------------------------------------------------------------------M  1
Smp_134140.3    --------------------------------------------------------------------------M  1
Smp_134140.5    MLKEKVRRVKLYKLTPVHQIKVISSNDTISSSQEPQLSPQIPAVVKPRRGSIFVSALAAEQHGASSLSTDNTGSM  75
SmPDE4A         --------------MELRTDKVISSNDTISSSQEPQLSPQIPAVVKPRRGSIFVSALAAEQHGASSLSTDNTGSM  61
                                ::  *******************************************************
encoded by:      exon 1 (5.e1/4A.e1)                 exon 2 (5.e2)

Smp_134140.4    ---------------------------------------------------------------------------  0
Smp_134140.1    IVRLGRSSTGGATAATSTSSSSTNGITAVLPDSSVEGVIVTPFAQVLVSMQRIRNAFIRLTAAQVSNKYNITTVF  76
Smp_134140.2    IVRLGRSSTGGATAATSTSSSSTNGITAVLPDSSVEGVIVTPFAQVLVSMQRIRNAFIRLTAAQVSNKYNITTVF  76
Smp_134140.3    IVRLGRSSTGGATAATSTSSSSTNGITAVLPDSSVEGVIVTPFAQVLVSMQRIRNAFIRLTAAQVSNKYNITTVF  76
Smp_134140.5    IVRLGRSSTGGATAATSTSSSSTNGITAVLPDSSVEGVIVTPFAQVLVSMQRIRNAFIRLTAAQVSNKYNITTVF  150
SmPDE4A         IVRLGRSSTGGATAATSTSSSSTNGITAVLPDSSVEGVIVTPFAQVLVSMQRIRNAFIRLTAAQVSNKYNITTVF  136
                ***************************************************************************
                                      exon 3 (1.e1/5.e3)

Smp_134140.4    ---------------------------------------------------------------------------  0
Smp_134140.1    DSVPTGNLTPDSSDYKIIANETLEELEWCLKQLENIQTKRPVSDMAFSKFKRLLNKELNSFGEADKSRHQISAYI  151
Smp_134140.2    DSVPTGNLTPDSSDYKIIANETLEELEWCLKQLENIQTKRPVSDMAFSKFKRLLNKELNSFGEADKSRHQISAYI  151
Smp_134140.3    DSVPTGNLTPDSSDYKIIANETLEELEWCLKQLENIQTKRPVSDMAFSKFKRLLNKELNSFGEADKSRHQISAYI  151
Smp_134140.5    DSVPTGNLTPDSSDYKIIANETLEELEWCLKQLENIQTKRPVSDMAFSKFKRLLNKELNSFGEADKSRHQISAYI  225
SmPDE4A         DSVPTGNLTPDSSDYKIIANETLEELEWCLKQLENIQTKRPVSDMAFSKFKRLLNKELNSFGEADKSRHQISAYI  211
                ***************************************************************************
                exon4 (1.e2)           exon 5 (1.e3)                  exon 6 (1.e4)

Smp_134140.4    ---------------------------------------------------------------------------  0
Smp_134140.1    CETFLETEKDVETNEEIDSMLERRRSSGQSHNSTSGQDTNTTSKRQASGTGDQNANPNTTRTPDTSSSVSSSVIK  226
Smp_134140.2    CETFLETEKDVETNEEIDSMLERRRSSGQSHNSTSGQDTNTTSKRQASGTGDQNANPNTTRTPDTSSSVSSSVIK  226
Smp_134140.3    CETFLETEKDVETNEEIDSMLERRRSSGQSHNSTSGQDTNTTSKRQASGTGDQNANPNTTRTPDTSSSVSSSVIK  226
Smp_134140.5    CETFLETEKDVETNEEIDSMLERRRSSGQSHNSTSGQDTNTTSKRQASGTGDQNANPNTTRTPDTSSSVSSSVIK  300
SmPDE4A         CETFLETEKDVETNEEIDSMLERRRSSGQSHNSTSGQDTNTTSKRQASGTGDQNANPNTTRTPDTSSSVSSSVIK  286
                ***************************************************************************
                                           exon 7 (1.e5)

Smp_134140.4    ---------------------------------------------------------------------------  0
Smp_134140.1    TRIGSTGSMSTESRKSAQLNDSSGLLTTKLPSSSKLTSQNVDDGNGPFLPIHGVETPNDNELEERFSLCLDEWGV  301
Smp_134140.2    TRIGSTGSMSTESRKSAQLNDSSGLLTTKLPSSSKLTSQNVDDGNGPFLPIHGVETPNDNELEERFSLCLDEWGV  301
Smp_134140.3    TRIGSTGSMSTESRKSAQLNDSSGLLTTKLPSSSKLTSQNVDDGNGPFLPIHGVETPNDNELEERFSLCLDEWGV  301
Smp_134140.5    TRIGSTGSMSTESRKSAQLNDSSGLLTTKLPSSSKLTSQNVDDGNGPFLPIHGVETPNDNELEERFSLCLDEWGV  375
SmPDE4A         TRIGSTGSMSTESRKSAQLNDSSGLLTTKLPSSSKLTSQNVDDGNGPFLPIHGVETPNDNELEERFSLCLDEWGV  361
                ***************************************************************************

Smp_134140.4    ---------------------------------------------------------------------------  0
Smp_134140.1    DIFEIDRLSNGHALTTVAYRIFQKRDLLKTFCIDPHVFVRYLLRVESTYHADVPYHNSMHAADVLQTAHFLLQAE  376
Smp_134140.2    DIFEIDRLSNGHALTTVAYRIFQKRDLLKTFCIDPHVFVRYLLRVESTYHADVPYHNSMHAADVLQTAHFLLQAE  376
Smp_134140.3    DIFEIDRLSNGHALTTVAYRIFQKRDLLKTFCIDPHVFVRYLLRVESTYHADVPYHNSMHAADVLQTAHFLLQAE  376
Smp_134140.5    DIFEIDRLSNGHALTTVAYRIFQKRDLLKTFCIDPHVFVRYLLRVESTYHADVPYHNSMHAADVLQTAHFLLQAE  450
SmPDE4A         DIFEIDRLSNGHALTTVAYRIFQKRDLLKTFCIDPHVFVRYLLRVESTYHADVPYHNSMHAADVLQTAHFLLQAE  436
                ***************************************************************************
                    exon 8 (1.e6)                       exon 9 (1.e7)

Smp_134140.4    ---------------------------------------------------------------------------  0
Smp_134140.1    ALDDVFSDLEILAVLFAAAIHDVDHPGVTNQFLINTGHELALQYNDASVLENHHLYMAFKILTEKDCDIFANLGG  451
Smp_134140.2    ALDDVFSDLEILAVLFAAAIHDVDHPGVTNQFLINTGHELALQYNDASVLENHHLYMAFKILTEKDCDIFANLGG  451
Smp_134140.3    ALDDVFSDLEILAVLFAAAIHDVDHPGVTNQFLINTAFTLIDVL-------------------------------  420
Smp_134140.5    ALDDVFSDLEILAVLFAAAIHDVDHPGVTNQFLINTGHELALQYNDASVLENHHLYMAFKILTEKDCDIFANLGG  525
SmPDE4A         ALDDVFSDLEILAVLFAAAIHDVDHPGVTNQFLINTGHELALQYNDASVLENHHLYMAFKILTEKDCDIFANLGG  511
                ************************************.. *    *******************************
                           exon 10 (1.e8)            exon 11 (1.e9/2.e9/3.e9)
```

```
Smp_134140.4   ------------------MSKHMSLLADLRTMVETKKVSGSGMLNLDNYADRIQILQNMIHCADLSNPAKPLRLY  57
Smp_134140.1   KKRQTLRRMVIELVLATDMSKHMSLLADLRTMVETKKVSGSGMLNLDNYADRIQILQNMIHCADLSNPAKPLRLY  526
Smp_134140.2   KKRQTLRRMVIELVRLIFNDIYIFCDICIYKRH------------------------------------------  484
Smp_134140.3   ---------------------------------------------------------------------------  420
Smp_134140.5   KKRQTLRRMVIELVRLIFNDIYIFCDICIYKRH------------------------------------------  558
SmPDE4A        KKRQTLRRMVIELVLATDMSKHMSLLADLRTMVETKKVSGSGMLNLDNYADRIQILQNMIHCADLSNPAKPLRLY  586
               **************     . ::     : .    ****************************************
                                         exon 12 (1.e10/4.e1)

Smp_134140.4   RKWTGRLIEEFFRQGDKERELSLEISPMCDRESVEVEKSQVSFIDFVCHPLWETWCDLVHPCAQLILDTLEDNRD  132
Smp_134140.1   RKWTGRLIEEFFRQGDKERELSLEISPMCDRESVEVEKSQVSFIDFVCHPLWETWCDLVHPCAQLILDTLEDNRD  601
Smp_134140.2   ---------------------------------------------------------------------------  484
Smp_134140.3   ---------------------------------------------------------------------------  420
Smp_134140.5   ---------------------------------------------------------------------------  558
SmPDE4A        RKWTGRLIEEFFRQGDKERELSLEISPMCDRESVEVEKSQVSFIDFVCHPLWETWCDLVHPCAQLILDTLEDNRD  661
               ***************************************************************************
                       exon 13 (1.e11)                            exon 14 (1.e12)

Smp_134140.4   WYECHIKESKMKVTQLARPKLATAAEDDEEISTTSGNT  170
Smp_134140.1   WYECHIKESKMKVTQLARPKLATAAEDDEEISTTSGNT  639
Smp_134140.2   --------------------------------------  484
Smp_134140.3   --------------------------------------  420
Smp_134140.5   --------------------------------------  558
SmPDE4A        WYECHIKESKMKVTQLARPKLATAAEDDEEISTTSGNT  699
               **************************************
```

SmPDE4B

```
Smp_141980.1   MIMWIICCRKQETKKNKTKDNLQLSSKYSISYNDHRINDNQDNCKLKNDLNIHTKLSHDSYANIENGHYP  70
Smp_141980.2   MIMWIICCRKQETKKNKTKDNLQLSSKYSISYNDHRINDNQDNCKLKNDLNIHTKLSHDSYANIENGHYP  70
SmPDE4B-a1     MIMWIICCRKQETKKNKTKDNLQLSSKYSISYNDHRINDNQDNCKLKNDLNIHTKLSHDSYANIENGHYP  70
SmPDE4B-a2     MIMWIICCRKQETKKNKTKDNLQLSSKYSISYNDHRINDNQDNCKLKNDLNIHTKLSHDSYANIENGHYP  70
               **********************************************************************
encoded by:                                exon 1 (1e.1)

Smp_141980.1   KQERRSLPDVVIPSTNLPIPRSSLQIDYETGRRSTIYLETTVKLAESESINLDDNYSSNIFNETFLNNIK  140
Smp_141980.2   KQERRSLPDVVIPSTNLPIPRSSLQIDYETGRRSTIYLETTVKLAESESINLDDNYSSNIFNETFLNNIK  140
SmPDE4B-a1     KQERRSLPDVVIPSTNLPIPRSSLQIDYETGRRSTIYLETTVKLAESESINLDDNYSSNIFNETFLNNIK  140
SmPDE4B-a2     KQERRSLPDVVIPSTNLPIPRSSLQIDYETGRRSTIYLETTVKLAESESINLDDNYSSNIFNETFLNNIK  140
               **********************************************************************

Smp_141980.1   LNMANRDFLLDKPRAHSLISSTYQKQLTNNHKGRQGRKSDVSRSDNHEKKLISKKRLSLFKVKPLKRFRK  210
Smp_141980.2   LNMANRDFLLDKPRAHSLISSTYQKQLTNNHKGRQGRKSDVSRSDNHEKKLISKKRLSLFKVKPLKRFRK  210
SmPDE4B-a1     LNMANRDFLLDKPRAHSLISSTYQKQLTNNHKGRQGRKSDVSRSDNHEKKLISKKRLSLFKVKPLKRFRK  210
SmPDE4B-a2     LNMANRDFLLDKPRAHSLISSTYQKQLTNNHKGRQGRKSDVSRSDNHEKKLISKKRLSLFKVKPLKRFRK  210
               **********************************************************************

Smp_141980.1   SKDSLHSLSPQKCTIRNDKSNMISTMTCDRSTNSKTKSSLKRIKPSKLFSTNKSSHENIGITKDLNNHES  280
Smp_141980.2   SKDSLHSLSPQKCTIRNDKSNMISTMTCDRSTNSKTKSSLKRIKPSKLFSTNKSSHENIGITKDLNNHES  280
SmPDE4B-a1     SKDSLHSLSPQKCTIRNDKSNMISTMTCDRSTNSKTKSSLKRIKPSKLFSTNKSSHENIGITKDLNNHES  280
SmPDE4B-a2     SKDSLHSLSPQKCTIRNDKSNMISTMTCDRSTNSKTKSSLKRIKPSKLFSTNKSSHENIGITKDLNNHES  280
               **********************************************************************

Smp_141980.1   CSERSEDSLGQLPMTRNSMFKHIQTRTTNLYQTTKNFDLNKQNYLNNIEHSMTNSKNTVLRRGSLCIFSQ  350
Smp_141980.2   CSERSEDSLGQLPMTRNSMFKHIQTRTTNLYQTTKNFDLNKQNYLNNIEHSMTNSKNTVLRRGSLCIFSQ  350
SmPDE4B-a1     CSERSEDSLGQLPMTRNSMFKHIQTRTTNLYQTTKNFDLNKQNYLNNIEHSMTNSKNTVLRRGSLCIFSQ  350
SmPDE4B-a2     CSERSEDSLGQLPMTRNSMFKHIQTRTTNLYQTTKNFDLNKQNYLNNIEHSMTNSKNTVLRRGSLCIFSQ  350
               **********************************************************************

Smp_141980.1   IDEPIVTPFAQILASLRKVRFNFILLTNVTSTRDSRFGAVQPIQSTEDGNSSSHLGCSGGQNKITASETL  420
Smp_141980.2   IDEPIVTPFAQILASLRKVRFNFILLTNVTSTRDSRFGAVQPIQSTEDGNSSSHLGCSGGQNKITASETL  420
SmPDE4B-a1     IDEPIVTPFAQILASLRKVRFNFILLTNVTSTRDSRFGAVQPIQSTEDGNSSSHLGCSGGQNKITASETL  420
SmPDE4B-a2     IDEPIVTPFAQILASLRKVRFNFILLTNVTSTRDSRFGAVQPIQSTEDGNSSSHLGCSGGQNKITASETL  420
               **********************************************************************
                                                    exon 2 (1.e2)

Smp_141980.1   EELEWCLERLENIQTHRSVSDMASSKFKKMLNKELSQFADAGQSGKQISEYICSTFLDSKENDPLTTTSH  490
Smp_141980.2   EELEWCLERLENIQTHRSVSDMASSKFKKMLNKELSQFADAGQSGKQISEYICSTFLDSKENDPLTTTSH  490
SmPDE4B-a1     EELEWCLERLENIQTHRSVSDMASSKFKKMLNKELSQFADAGQSGKQISEYICSTFLDSKENDPLTTTSH  490
SmPDE4B-a2     EELEWCLERLENIQTHRSVSDMASSKFKKMLNKELSQFADAGQSGKQISEYICSTFLDSKENDPLTTTSH  490
               **********************************************************************
                    exon 3 (1.e3)

Smp_141980.1   SSVISHNNTHGIMSNSINKDIMINNHDNPSEMNTCTPSDSIKNSESTGMYLFKSVEPGEIIQTTTTTTTL  560
Smp_141980.2   SSVISHNNTHGIMSNSINKDIMINNHDNPSEMNTCTPSDSIKNSESTGMYLFKSVEPGEIIQTTTTTTTL  560
SmPDE4B-a1     SSVISHNNTHGIMSNSINKDIMINNHDNPSEMNTCTPSDSIKNSESTGMYLFKSVEPGEIIQTTTTTTTL  560
SmPDE4B-a2     SSVISHNNTHGIMSNSINKDIMINNHDNPSEMNTCTPSDSIKNSESTGMYLFKSVEPGEIIQTTTTTTTL  560
               **********************************************************************
                                  exon 4 (1.e4/4B-a1.e4/4B-a2.e4)

                                       o∆∆
Smp_141980.1   TDNIDGDTTIGVTGDSSSSNIPHTINSTTVTIGSTTMNKSLSKLSSSLSTLSLKMNSNVNVNENTNNNNN  630
Smp_141980.2   TDNIDGDTTIGVTGDSSSSNIPHTINSTTVTIGSTTMNKSLSKLSSSLSTLSLKMNSNVNVNENTNNNNN  630
SmPDE4B-a1     TDNIDGDTTIGVTGDSSSSNIPHTINSTTVTIGSTTMNKSLSKLSSSLSTLSLKMNSNVNVNENTNNNNN  630
SmPDE4B-a2     TDNIDGDTTIGVTGDSSSSNIPHTC--TTVTIGSTTMNKSLSKLSSSLSTLSLKMNSNVNVNENTNNNNN  628
               ************************   *******************************************
```

```
Smp_141980.1   NNDDNNINIMMKSSGNSHTTMSIPPSGTSPLSHQHHSECDKIHCSQTQIVPYIINSTNPKKLEDLLKTSL   700
Smp_141980.2   NNDDNNINIMMKSSGNSHTTMSIPPSGTSPLSHQHHSECDKIHCSQTQIVPYIINSTNPKKLEDLLKTSL   700
SmPDE4B-a1     NNDDNNINIMMKSSGNSHTTMSIPPSGTSPLSHQHHSECDKIHCSQTQIVPYIINSTNPKKLEDLLKTSL   700
SmPDE4B-a2     NNDDNNINIMMKSSGNSHTTMSIPPSGTSPLSHQHHSECDKIHCSQTQIVPYIINSTNPKKLEDLLKTSL   698
               **********************************************************************

Smp_141980.1   DLWGIDIFEVDQLTTNPLTCIFYNIVQKRNLLQKFAIPERNLLLYMTAVEEKYNNNPYHNRVHAADVVQS   770
Smp_141980.2   DLWGIDIFEVDQLTTNPLTCIFYNIVQKRNLLQKFAIPERNLLLYMTAVEEKYNNNPYHNRVHAADVVQS   770
SmPDE4B-a1     DLWGIDIFEVDQLTTNPLTCIFYNIVQKRNLLQKFAIPERNLLLYMTAVEEKYNNNPYHNRVHAADVVQS   770
SmPDE4B-a2     DLWGIDIFEVDQLTTNPLTCIFYNIVQKRNLLQKFAIPERNLLLYMTAVEEKYNNNPYHNRVHAADVVQS   768
               **********************************************************************
                     exon 5 (1.e5)                        exon 6 (1.e6)

Smp_141980.1   THVLLNAQSLESVFTDLEIFTVLFACAIHDVGHPGVTNQYLINTNDQLAILYNDSSVLENHHLAIAFSLL   840
Smp_141980.2   THVLLNAQSLESVFTDLEIFTVLFACAIHDVGHPGVTNQYLINTNDQLAILYNDSSVLENHHLAIAFSLL   840
SmPDE4B-a1     THVLLNAQSLESVFTDLEIFTVLFACAIHDVGHPGVTNQYLINTNDQLAILYNDSSVLENHHLAIAFSLL   840
SmPDE4B-a2     THVLLNAQSLESVFTDLEIFTVLFACAIHDVGHPGVTNQYLINTNDQLAILYNDSSVLENHHLAIAFSLL   838
               **********************************************************************
                            exon 7 (1.e7)                        exon 8 (1.e8)

Smp_141980.1   GQPGHDVFENFPRKQRLSSRRMIIDMVLATDMSKHMSLLADLKTMVETKKVAGSGILTLENYIDRMQILQ   910
Smp_141980.2   GQPGHDVFENFPRKQRLSSRRMIIDMVLATDMSKHMSLLADLKTMVETKKVAGSGILTLENYIDRMQILQ   910
SmPDE4B-a1     GQPGHDVFENFPRKQRLSSRRMIIDMVLATDMSKHMSLLADLKTMVETKKVAGSGILTLENYIDRMQILQ   910
SmPDE4B-a2     GQPGHDVFENFPRKQRLSSRRMIIDMVLATDMSKHMSLLADLKTMVETKKVAGSGILTLENYIDRMQILQ   908
               **********************************************************************
                                                       exon 9 (1.e9)

Smp_141980.1   NMVHCADLSNPAKPLDLYRQWTNRVMEELFQQGDKERELGIEISPICDRNTATIEKSQVSFIDYIVHPLW   980
Smp_141980.2   NMVHCADLSNPAKPLDLYRQWTNRVMEELFQQGDKERELGIEISPICDRNTATIEKSQAPDILLSSPQFC   980
SmPDE4B-a1     NMVHCADLSNPAKPLDLYRQWTNRVMEELFQQGDKERELGIEISPICDRNTATIEKSQVSFIDYIVHPLW   980
SmPDE4B-a2     NMVHCADLSNPAKPLDLYRQWTNRVMEELFQQGDKERELGIEISPICDRNTATIEKSQVSFIDYIVHPLW   978
               **********************************************************.   *      :
                             exon 10 (1.e10)                  exon 11 (1.e11/2.e11)

Smp_141980.1   ETWSDLVYPDAQTILETLEDNREWYYNQINENNNDNNAENDE   1022
Smp_141980.2   KQHASARR----------------------------------   988
SmPDE4B-a1     ETWSDLVYPDAQTILETLEDNREWYYNQINENNNDNNAENDE   1022
SmPDE4B-a2     ETWSDLVYPDAQTILETLEDNREWYYNQINENNNDNNAENDE   1020
               :  :.
```

SmPDE4C

```
Smp_334600.1   MTTISTNIHSKSIGNSLYERRRNWRIHQMKSCMEPPINVSTTSIYNKDDDDGIRMNIMSKKSDILCNKEN  70
SmPDE4C        MTTISTNIHSKSIGNSLYERRRNWRIHQMKSCMEPPINVSMTSIYNKDDDDGIRMNIMSKKSDILCNKEN  70
               **************************************** *****************************
encoded by                                 exon 1 (1.e1/4C.e1)

Smp_334600.1   WWGKTISHEPSVLITPFAQILAILNRARDFLSNYTSNSPSYSPHDPNMCSTKSIGCDEKYVNCLHRFDVH  140
SmPDE4C        WWGKTISHEPSVLITPFAQILAILNRARDFLSNYTSNSPSYSPHDPNMCSTKSIGCDEKYVNCLHRFDVH  140
               **********************************************************************
                              exon 2 (1.e2)                  exon 3 (1.e3)

Smp_334600.1   YVNQLLNEFDWCLEVLDSLQSKRSVSSLTRMKLRSLLSQELAASFNQSNDKIIKKDSLKKSTTTTHNVRF  210
SmPDE4C        YVNQLLNEFDWCLEVLDSLQSKRSVSSLTRMKLRSLLSQELAASFNQSNGKIIKKDSLKKSTTTTHNVRF  210
               ************************************************* ********************
                        exon 4 (1.e4)

Smp_334600.1   DDLTLKNSSLLVHDQDNNNKRSNISQIQKRKQQFRSCRSQVCEYICKTFLEEDDDDEDVDNNHEDDDGDA  280
SmPDE4C        DDLTLKNSSLLVHDQDNNNKRSNISQIQKRKQQFRSCRSQVCEYICKTFLEEDDDDEDVDNNHEDDDGDA  280
               **********************************************************************
                                             exon 5 (1.e5/4C.e5)

Smp_334600.1   EEDNSKSISSHNPSTEKSIHLNSPKSKLRFSDKYEERMNESSETTESTTELKSTSSTQFNDTDVISDTLK  350
SmPDE4C        EEDNSKSISSHNPSTEKSIHLNSPKSKLRFSDKYEERMNESSETTESTTELKSTSSTQFNDTDVISDTLK  350
               **********************************************************************

Smp_334600.1   LIILNIEHVDHSIVENFIINNQSNLAPDLFKLDQISNHHPLSTFGFYLFMKTNVLQKLSIPSVTMLNCLR  420
SmPDE4C        LIILNIEHVDHSIVENFIINNQSNLAPDLFKLDQISNHHPLSTFGFYLFMKTNVLQKLSIPSVTMLNCLR  420
               **********************************************************************
                                            exon 6 (1.e6)

Smp_334600.1   QIESRYNSTAPFHNSIHALDVLHATYQLFQCNSLKNIFSDLETFAIFFASAIHDIDHPGLTNQYLINTNH  490
SmPDE4C        QIESRYNSTAPFHNSIHALDVLHATYQLFQCNSLKNIFSDLETFAIFFASAIHDIDHPGLTNQYLINTNH  490
               **********************************************************************
                   exon 7 (1.e7)                          exon 8 (1.e8)

Smp_334600.1   ELALLYNDISVLENHHLHVAFKLINTQIECDFTKYFTNQQKLLFRKMVIALVLSTDMSKHMSLLADLKTS  560
SmPDE4C        ELALLYNDISVLENHHLHVAFKLINTQIECDFTKYFTNQQKLLFRKMVIALVLSTDMSKHMSLLADLKTS  560
               **********************************************************************
                               exon 9 (1.e9)                        exon 10 (1.e10)

Smp_334600.1   VEKQKAFQGNVINLDSYSARMQILECIIHAADLSNPTKPLKIYQEWVSRIMEEMFRQGDQEKQYGIEISP  630
SmPDE4C        VEKQKAFQGNVINLDSYSARMQILECIIHAADLSNPTKPLKIYQEWVSRIMEEMFRQGDQEKQYGIEISP  630
               **********************************************************************
                                                          exon 11 (1.e11)

Smp_334600.1   MCDRETACIYSTQIGFIDYIVYPLWETMAELLHPGLQVLMDNITNNRNWYVKAKEEEEEEEVKEENKKNN  700
SmPDE4C        MCDRETACIYSTQIGFIDYIVYPLWETMAELLHPGLQVLMDNITNNRNWYVKAKEEEEEE-VKEENKKNN  699
               ************************************************************ *********
                                                exon 12 (1.e12/4C.e12)

Smp_334600.1   SIDQ   704
SmPDE4C        SIDQ   703
               ****
```

SmPDE7var

```
Smp_153640.1   --------------------------------------------------------------------------------   0
Smp_153640.2   MRRSRTDQKSGSWSHMEVDLTNKNLQRTKNSSCRRIRYRSNSFNAFVCQQSEQMAGTIQLFYLYEDKNSKNMYNLDKSKE  80
SmPDE7var-a1   MRRSRTDQKSGSWSHMEVDLTNKNLQRTKNSSCRRIRYRSNSFNAFVCQQSEQMAGTIQLFYLYEDKNSKNMYNLDKSKE  80
SmPDE7var-a2   MRRSRTDQKSGSWSHMEVDLTNKNLQRTKNSSCRRIRYRSNSFNAFVCQQSEQMAGTIQLFYLYEDKNSKNMYNLDKSKE  80
               ********************************************************************************
encoded by:       exon 1 (2.e2)                                            exon 2 (2.e3)

Smp_153640.1   ---------------------------MNVMDMMKLNVDNLKIIISDYTIRDIGLQIMNEYDLFQKLKLNYFMMARIFNS  53
Smp_153640.2   RKKSQTVERITDEIKLSFIDRPVGHVKIKNLSNWDFNIFHLKRTSSNYTIRDIGLQIMNEYDLFQKLKLNYFMMARIFNS 160
SmPDE7var-a1   RKKSQTVERITDEIKLSFIDRPVGHVKIKNLSNWDFNIFHLKRTSSNYTIRDIGLQIMNEYDLFQKLKLNYFMMARIFNS 160
SmPDE7var-a2   RKKSQTVERITDEIKLSFIDRPVGHVKIKNLSNWDFNIFHLKRTSSNYTIRDIGLQIMNEYDLFQKLKLNYFMMARIFNS 160
               ***************************:: :.  .:*: .**   *:*********************************
                                          exon 3 (1.e3/2.e4)         exon 4 (1.e4/2.e5)

Smp_153640.1   IEAAYHNFNPYHTALHAADVLQAVHCFISRSQLLTILSPTEIFASLLAAALHDADHPGVNQSYLEKTGDFLVDLYKSVSV 133
Smp_153640.2   IEAAYHNFNPYHTALHAADVLQAVHCFISRSQLLTILSPTEIFASLLAAALHDADHPGVNQSYLEKTGDFLVDLYKSVSV 240
SmPDE7var-a1   IEAAYHNFNPYHTALHAADVLQAVHCFISRSQLLTILSPTEIFASLLAAALHDADHPGVNQSYLEKTGDFLVDLYKSVSV 240
SmPDE7var-a2   IEAAYHNFNPYHTALHAADVLQAVHCFISRSQLLTILSPTEIFASLLAAALHDADHPGVNQSYLEKTGDFLVDLYKSVSV 240
               ********************************************************************************
                    exon 5 (1.e5)                            exon 6 (1.e6)

Smp_153640.1   LEKHHAKFGLCILQENGLSNALELHEWEFVRDCFLKLIPATDITYQGVYQKQFKDLTNYHMANPSLPFTISDRLLIMQMA 213
Smp_153640.2   LEKHHAKFGLCILQENGLSNALELHEWEFVRDCFLKLIPATDITYQGVYQKQFKDLTNYHMANPSLPFTISDRLLIMQMA 320
SmPDE7var-a1   LEKHHAKFGLCILQENGLSNALELHEWEFVRDCFLKLIPATDITYQGVYQKQFKDLTNYHMANPSLPFTISDRLLIMQMA 320
SmPDE7var-a2   LEKHHAKFGLCILQENGLSNALELHEWEFVRDCFLKLIPATDITYQGVYQKQFKDLTNYHMANPSLPFTISDRLLIMQMA 320
               ********************************************************************************
                  exon 7 (1.e7)            exon 8 (1.e8)            exon 9 (1.e9)

Smp_153640.1   LKCSDISNPCRIWPICKEWAIRVCCELFCQGDRERFQWSLQPIPTMDRTKFTLARIQNGFIRDMVKPLLTGWHEFLQNNL 293
Smp_153640.2   LKCSDISNPCRIWPICKEWAIRVCCELFCQGDRERFQWSLQPIPTMDRTKFTLARIQNGFIRDMVKPLLTGWHEFLQNNL 400
SmPDE7var-a1   LKCSDISNPCRIWPICKEWAIRVCCELFCQGDRERFQWSLQPIPTMDRTKFTLARIQNGFIRDMVKPLLTGWHEFLQNNL 400
SmPDE7var-a2   LKCSDISNPCRIWPICKEWAIRVCCELFCQGDRERFQWSLQPIPTMDRTKFTLARIQNGFIRDMVKPLLTGWHEFLQNNL 400
               ********************************************************************************
                                 exon 10 (1.e10)

Smp_153640.1   TLKILQNLDENLKNWLTDLSLSSSSSTSSSSGMTNYVNRHNSLDSQLLSTLPLSNNNNNQSISSNQIKPKTIKSEISNQK 373
Smp_153640.2   TLKILQNLDENLKNWLTDLSLSSSSSTSSSSGMTNYVNRHNSLDSQLLSTLPLSNNNNNQSISSNQIKPKTIKSEISNQK 480
SmPDE7var-a1   TLKILQNLDENLKNWLTDLSLSSSSSTSSSSGMTNYVNRHNSLDSQLLSTLPLSNNNNNQSISSNQIKPKTIKSEISNQK 480
SmPDE7var-a2   TLKILQNLDENLKNWLTDLSLSSSSSTSSSSGMTNYVNRHNSLDSQLLSTLPLSNNNNNQSISSNQIKPKTIKSEISDQK 480
               *****************************************************************************:**
                                          exon 11 (1.e11)

Smp_153640.1   SKIELQKPFIGSIHLTHSPIQEIETMNASESLDSTTTNNNNLHSGKLHQTIFITTRVIRRHSLPETQLAIRKTFNFSLSN 453
Smp_153640.2   SKIELQKPFIGSIHLTHSPIQEIETMNASESLDSTTTNNNNLHSGKLHQTIFITTRVIRRHSLPETQLAIRKTFNFSLSN 560
SmPDE7var-a1   SKIELQKPFIGSIHLTHSPIQEIETMNASESLDSTTTNNNNLHSGKLHQTIFITTRVIRRHSLPETQLAIRKTFNFSLSN 560
SmPDE7var-a2   SKIELQKPFIGSIHLTHSPIQEIETMNASESLDSTTTNNNNLHSGKLHQTIFITTRVIRRHSLPETQLAIRKTFNFSLSN 560
               ********************************************************************************
                                 exon 12 (1.e12/2.e13/7var-a1.e12/7var-a2.e12)

Smp_153640.1   KSSTVVLLKNDTQNILSLHSRNNNNLPVGKIKCQSSTFKSPSSSLSPSSTCTVSANLLQMLYEELKHNNKSNYVLSKIDI 533
Smp_153640.2   KSSTVVLLKNDTQNILSLHSRNNNNLPVGKIKCQSSTFKSPSSSLSPSSTCTVSANLLQMLYEELKHNNKSNYVLSKIDI 640
SmPDE7var-a1   KSSTVVLLKNDTQNILSLHSRNNNNLPVGKIKCQSSTFKSPSSSLSPSSTCTVSANLLQMLYEELKHNNKSNYVLSKIDI 640
SmPDE7var-a2   KSSTVVLLKNDTQNILSLHSRNNNNLPVGKIKCQSSTFKSPSSSLSPSSTCTVSANLLQMLYEELKHNNKSNYVLSKIDI 640
               ********************************************************************************

Smp_153640.1   KSIPNTNCYKDNKLLEPKLLDLNYDRTLLRFSALAHRRSSAPITEH 579
Smp_153640.2   KSIPNTNCYKDNKLLEPKLLDLNYDRTLLRFSALAHRRSSAPITEH 686
SmPDE7var-a1   KSIPNTNCYKDNKLLEQKVLDLNYDRTLLRFSALAHRRSSAPITEH 686
SmPDE7var-a2   KSIPNTNCYKDNKLLEQKVLDLNYDRTLLRFSALAHRRSSAPITEH 686
               **************** *:**************************
```

SmPDE8

```
Smp_044060.1  MGCVNTKSILNKSIEDIEKPEVLLKIVLIFSQPDKQTQFMINAAQQLKHNCILINTMMDNNTTKDIDKIM  70
SmPDE8        ----------------------------------------------------------------------  0
encoded by:        exon 1 (1.e1)                    exon 2 (1.e2)

Smp_044060.1  NEKLIDLQQIIDMVVLDLRKLKSNGSFSIPSIRSSQCREIIISNNNISPSLSNWCKQLRSYPPTKDSVIV  140
SmPDE8        ----------------------------------------------------------------------  0
                                         exon 3 (1.e3)

Smp_044060.1  GLLSRKCSNDQRKILMNKALKSGCNKCIFEASTVDDFYEELKNFANNEWKLLNQLRNFIYTNNITNNTTN  210
SmPDE8        ----------------------------------------------------------------------  0
                       exon 4 (1.e4)                          exon 5 (1.e5)

Smp_044060.1  NTTTSPPPTTSTTTNNNTITTKATNIITTSNNNDFIVESENNQSNLELNEISPKDECRYQMKSETRQPTI  280
SmPDE8        ------------------------------------------------------------MKSETRQPTI  10
                                                                          **********

Smp_044060.1  KESVDNEITSTDELKANVIDSVTVLHEQGKKFREFISLVFITEFYTNNIVSLSALLHSRCPDFSSPMCKV  350
SmPDE8        KESVDNEITSTDELKANVIDSVTVLHEQGKKFREFISVRKNRNSDTNNIVSLSALLHSRCPDFSSPMCKV  80
              *************************************:    :  *************************
                    exon 6 (1.e6)          exon7    exon8        exon 9 (1.e9)
                                       (1.e7/8.e7) (1.e8)

Smp_044060.1  IGILNSARVRSPLPVAKDLQKAINLICSSNVFVDQIMKPLSRTNDPITADLIEGLITGSNLAREPENLLK  420
SmPDE8        IGILNSARVRSPLPVAKDLQKAINLICSSNVFVDQIMKPLSRTNDPITADLIEGLITGSNLAREPENLLK  150
              **********************************************************************
                                exon 10 (1.e10)                      exon 11 (1.e11)

Smp_044060.1  LRSLAKSLKGSENASTTLSTLKNSPEIEACLSNFDKWDFNIFDLERITNKKPLTCLGMKILDSFNALSVL  490
SmPDE8        LRSLAKSLKGSENASTTLSTLKNSPEIEACLSNFDKWDFNIFDLERITNKKPLTCLGMKILDSFNALSVL  220
              **********************************************************************
                                  exon 12 (1.e12)

Smp_044060.1  RIPSQILVGWLTVIEEHYHVDNPYHNATHAGDVLQASAYFLQHSLIRSICTNIDEVATLLAAIVHDVDHP  560
SmPDE8        RIPSQILVGWLTVIEEHYHVDNPYHNATHAGDVLQASAYFLQHSLIRSICTNIDEVATLLAAIVHDVDHP  290
              **********************************************************************
                           exon 13 (1.e13)                       exon 14 (1.e14)

Smp_044060.1  GKTNPFLVNSNDPLAILYNDIAVLESHHAAVSFELTLRSPDINIFQNLTREEYRTMRSYIVDMVLATEMV  630
SmPDE8        GKTNPFLVNSNDPLAILYNDIAVLESHHAAVSFELTLRSPDINIFQNLTREEYRTMRSYIVDMVLATEMV  360
              **********************************************************************
                                         exon 15 (1.e15)          exon 16 (1.e16)

                                           Insertion compared to human PDE8
Smp_044060.1  RHFDIVTKFVNTLSKPMLAKNRHHDRSSVGSMSSMESCSMGMTISHSTSPSPGQERISSTLENLSTAENR  700
SmPDE8        RHFDIVTKFVNTLSKPMLAKNRHHDRSSVGSMSSMESCSMGMTISHSTSPSPGQERISSTLENLSTAENR  430
              **********************************************************************
                                                         exon 17 (1.e17)

Smp_044060.1  TLIKRLIIKCSDVNNPTRPLSICKEWATRIAEEYFCQTEEEKRRNLPIVMPNFDRQTCNISQSQLSFIDF  770
SmPDE8        TLIKRLIIKCSDVNNPTRPLSICKEWATRIAEEYFCQTEEEKRRNLPIVMPNFDRQTCNISQSQLSFIDF  500
              **********************************************************************
                                                           exon 18 (1.e18)

Smp_044060.1  FLKGMFSGFDCVFPIPELMNNLENNTTYWASNIDREKKQHGTCPVELKPTTIHQE  825
SmPDE8        FLKGMFSGFDCVFPIPELMNNLENNTTYWASNIDREKKQHGTCPVELKPTTIHQE  555
              *******************************************************
                                  exon 19 (1.e19)
```

SmPDE9A

```
Smp_197150.1   MGSVISKLTPKVIYLLINGNIERILITLSCTSFEIHDLICILSNVPKSSNIIITDVNGLHIPCSGSMLAN  70
SmPDE9A        MGSVISKLTPKVIYLLINGNIERILITLSCTSFEIHDLICILSNVPKSSNIIITDVNGLHIPCSGSMLAN  70
               **********************************************************************
encoded by:        exon 1 (1.e1)           exon 2 (1.e2)           exon 3 (1.e3)

Smp_197150.1   TYNTPYTVTITQPSEPSEISLIVRMFESIIKQINDTMKISDLKNEFTERIQLLEQRVMVESDRYNDIDVI  140
SmPDE9A        TYNTPYTVTITQPSEPSEISLIVRMFESIIKQINDTMKISDLKNEFTERIQLLEQRVMVESDRYNDIDVI  140
               **********************************************************************
                        exon 4 (1.e4/9A.e4)           exon 5 (1.e5)

Smp_197150.1   KKELKQLKIQIHERKTGLTNIGSERTYLGNIRLSNDGVKILELQNLPIFEKYTLTQSTIDFLKKPTFDIW  210
SmPDE9A        KKELKQLKIQIHERKTGLTNIGSERTYLGNIRLSNDGVKILELQNLPIFEKYTLTQSTIDFLKKPTFDIW  210
               **********************************************************************
                  exon 6 (1.e6)             exon 7 (1.e7)             exon 8 (1.e8)

Smp_197150.1   HWEPNEMLALLEHMYNELGVVSEFNINPLTLKRWLLSIQANYRNNPFHNFRHCFCVAQMMYGILYLCGLN  280
SmPDE9A        HWEPNEMLALLEHMYNELGVVSEFNINPLTLKRWLLSIQANYRNNPFHNFRHCFCVAQMMYGILYLCGLN  280
               **********************************************************************
                           exon 9 (1.e9)                       exon 10 (1.e10)

Smp_197150.1   NDFSREELGILLTAAVCHDLDHPGYSNSYQINARTELAIRYNDISPLENHHCAVAFSILNHPELNIFANV  350
SmPDE9A        NDFSREELGILLTAAVCHDLDHPGYSNSYQINARTELAIRYNDISPLENHHCAVAFSILNHPELNIFANV  350
               **********************************************************************
                                                             exon 11 (1.e11)

Smp_197150.1   NQEVFRRIRQGMTSLILSTDMARHGEILETMRRHLEEGFSMNKKEHRETFKMVLIKCCDISNEVRPLSVS  420
SmPDE9A        NQEVFRRIRQGMTSLILSTDMARHGEILETMRRHLEEGFSMNKKEHRETFKMVLIKCCDISNEVRPLSVS  420
               **********************************************************************
                                  exon 12 (1.e12)                   exon 13 (1.e13)

Smp_197150.1   EPWVDCLLEEYFNQSDREKLEGLPVAPFMDREKVTKPTAQIGFIKFVLIPMFQTVASVVYPIIDELMVTQL  490
SmPDE9A        EPWVDCLLEEYFNQSDREKLEGLPVAPFMDREKVTKPTAQIGFIKFVLIPMFQTVASVVYPIIDELMVTQL  490
               **********************************************************************
                                       exon 14 (1.e14/9A.e14)

Smp_197150.1   KSALERYEKMLAEEEETKRNLQLNEAD  517
SmPDE9A        KSALERYEKMLAEEEETKRNLQLNEAD  517
               ***************************
                    exon 15 (1.e15)
```

SmPDE9B

```
                 *
Smp_146120.1   MNATKCEEDITSDHKISNITISQNNTNDNTRTICKPAIQTTKTTTTLPRKLNADNDDNNTNSFLSLYTRC   70
Smp_146120.2   -------MKSDADHKISNITISQNNTNDNTRTICKPAIQTTKTTTTLPRKLNADNDDNNTNSFLSLYTRC   63
SmPDE9B-a1     MNATKCEEDITSDHKISNITISQNNTNDNTRTICKPAIQTTKTTTTLPRKLNADNDDNNTNSFLSLYTRC   70
SmPDE9B-a2     MNATKCEEDITSDHKISNITISQNNTNDNTRTICKPAIQTTKTTTTLPRKLNADNDDNNTNSFLSLYTRC   70
                       .  :**********************************************************
encoded by:     exon 1
               (1.e1/2.e1)

                                oΔΔ
Smp_146120.1   IKNPNRRKKKQLDNNNNNNNNSNHNHQSHFCLNFCRNCSSNTPSSSSSSSSSSTATTEAGASVAVAATTG   140
Smp_146120.2   IKNPNRRKKKQLDNNNNNNNNSNHNHQSHFCLNFCRNCSSNTPSSSSSSSSSSTATTEAGASVAVAATTG   133
SmPDE9B-a1     IKNPNRRKKKQLDNNNNNNNNSNHNHQSHFCLNFCRNCSSNTPSSSSSSSSSSTATTEAGASVAVAATTG   140
SmPDE9B-a2     IKNPNRRKKKQLDNNNNT--NSNHNHQSHFCLNFCRNCSSNTPSSSSSSSSSSTATTEAGASVAVAATTG   138
               *****************.  **************************************************
                                      exon 2 (1.e2/9B-a2.e2)

Smp_146120.1   RKESNSFSLPFPLDNKSHDSLISYSSKITNNTNFTNDISSPGTKVTLTPNHTNNIIMSVSNCSNQFNSIS   210
Smp_146120.2   RKESNSFSLPFPLDNKSHDSLISYSSKITNNTNFTNDISSPGTKVTLTPNHTNNIIMSVSNCSNQFNSIS   203
SmPDE9B-a1     RKESNSFSLPFPLDNKSHDSLISYSSKITNNTNFTNDISSPGTKVTLTPNHTNNIIMSVSNCSNQFNSIS   210
SmPDE9B-a2     RKESNSFSLPFPLDNKSHDSLISYSSKITNNTNFTNDISSPGTKVTLTPNHTNNIIMSVSNCSNQFNSIS   208
               **********************************************************************

Smp_146120.1   VDPDKKDNHKIDAQNTSVHINKRQTADKFHSNTTEYQSKSNMTTTVMTSIPTTTTIEPINVHKKINPSLM   280
Smp_146120.2   VDPDKKDNHKIDAQNTSVHINKRQTADKFHSNTTEYQSKSNMTTTVMTSIPTTTTIEPINVHKKINPSLM   273
SmPDE9B-a1     VDPDKKDNHKIDAQNTSVHINKRQTADKFHSNTTEYQSKSNMTTTVMTSIPTTTTIEPINVHKKINPSLM   280
SmPDE9B-a2     VDPDKKDNHKIDAQNTSVHINKRQTADKFHSNTTEYQSKSNMTTTVMTSIPTTTTIEPINVHKKINPSLM   278
               **********************************************************************

Smp_146120.1   IADNSAYFNDNHENLLEISKLPIYDSTKLCIKCQKNIQLVSKDYEHILLLTNNQGKHEKITVNNNSIINN   350
Smp_146120.2   IADNSAYFNDNHENLLEISKLPIYDSTKLCIKCQKNIQLVSKDYEHILLLTNNQGKHEKITVNNNSIINN   343
SmPDE9B-a1     IADNSAYFNDNHENLLEISKLPIYDSTKLCIKCQKNIQLVSKDYEHILLLTNNQGKHEKITVNNNSIINN   350
SmPDE9B-a2     IADNSAYFNDNHENLLEISKLPIYDSTKLCIKCQKNIQLVSKDYEHILLLTNNQGKHEKITVNNNSIINN   348
               **********************************************************************

Smp_146120.1   NNNGNGYMEHTTEMIDDIKSKKIKEMESDIEEYVKHVQHIFDHINLTKDQFCQTDSKSNYIIDNKQLWDA   420
Smp_146120.2   NNNGNGYMEHTTEMIDDIKSKKIKEMESDIEEYVKHVQHIFDHINLTKDQFCQTDSKSNYIIDNKQLWDA   413
SmPDE9B-a1     NNNGNGYMEHTTEMIDDIKSKKIKEMESDIEEYVKHVQHIFDHINLTKDQFCQTDSKSNYIIDNKQLWDA   420
SmPDE9B-a2     NNNGNGYMEHTTEMIDDIKSKKIKEMESDIEEYVKHVQHIFDHINLTKDQFCQTDSKSNYIIDNKQLWDA   418
               **********************************************************************

Smp_146120.1   FNTSYANQEKIFRPSHNDINIYHLDDDMNNGDVNDDNDEEIIEAHYDNVHSYRKCNHFSVDKMDKKRISK   490
Smp_146120.2   FNTSYANQEKIFRPSHNDINIYHLDDDMNNGDVNDDNDEEIIEAHYDNVHSYRKCNHFSVDKMDKKRISK   483
SmPDE9B-a1     FNTSYANQEKIFRPSHNDINIYHLDDDMNNGDVNDDNDEEIIEAHYDNVHSYRKCNHFSVDKMDKKRISK   490
SmPDE9B-a2     FNTSYANQEKIFRPSHNDINIYHLDDDMNNGDVNDDNDEEIIEAHYDNVHSYRKCNHFSVDKMDKKRISK   488
               **********************************************************************
                                           exon 3 (1.e3)

Smp_146120.1   IYRQLHNLRCQVESFSYLSWLGLTAEQPPTQKVLVPGFNAPAPNPQMHLIRRSDADSRRNIQEFKLLCKE   560
Smp_146120.2   IYRQLHNLRCQVESFSYLSWLGLTAEQPPTQKVLVPGFNAPAPNPQMHLIRRSDADSRRNIQEFKLLCKE   553
SmPDE9B-a1     IYRQLHNLRCQVESFSYLSWLGLTAEQPPTQKVLVPGFNAPAPNPQMHLIRRSDADSRRNIQEFKLLCKE   560
SmPDE9B-a2     IYRQLHNLRCQVESFSYLSWLGLTAEQPPTQKVLVPGFNAPAPNPQMHLIRRSDADSRRNIQEFKLLCKE   558
               **********************************************************************
                                                exon 4 (1.e4)

Smp_146120.1   PVSKEDLIELRSSTFNNWSRTDAQLIRLVREMFQELGFIEHYNIQLHQLDLWLTDIYRRYNRVPFHNYKH   630
Smp_146120.2   PVSKEDLIELRSSTFNNWSRTDAQLIRLVREMFQELGFIEHYNIQLHQLDLWLTDIYRRYNRVPFHNYKH   623
SmPDE9B-a1     PVSKEDLIELRSSTFNNWSRTDAQLIRLVREMFQELGFIEHYNIQLHQLDLWLTDIYRRYNRVPFHNYKH   630
SmPDE9B-a2     PVSKEDLIELRSSTFNNWSRTDAQLIRLVREMFQELGFIEHYNIQLHQLDLWLTDIYRRYNRVPFHNYKH   628
               **********************************************************************
                                           exon 5 (1.e5)
```

```
Smp_146120.1   AFMVTQMCYVLIWGGNLTNLLDIDDQLILIVSAICHDLDHPGFNNAYQINAGTVLAMRYNDQSPLENHHT  700
Smp_146120.2   AFMVTQMCYVLIWGGNLTNLLDIDDQLILIVSAICHDLDHPGFNNAYQINAGTVLAMRYNDQSPLENHHT  693
SmPDE9B-a1     AFMVTQMCYVLIWGGNLTNLLDIDDQLILIVSAICHDLDHPGFNNAYQINAGTVLAMRYNDQSPLENHHT  700
SmPDE9B-a2     AFMVTQMCYVLIWGGNLTNLLDIDDQLILIVSAICHDLDHPGFNNAYQINAGTVLAMRYNDQSPLENHHT  698
               **********************************************************************
encoded by:                         exon 6 (1.e6)                   exon 7 (1.e7)

Smp_146120.1   AVAFDLLSHKEVDPFSHLSTTTRQRIRKGVIRCILATDMSRHNEILDEFNRQVLTDLNAAWEIDPNTKKP  770
Smp_146120.2   AVAFDLLSHKEVDPFSHLSTTTRQRIRKGVIRCILATDMSRHNEILDEFNRQVLTDLNAAWEIDPNTKKP  763
SmPDE9B-a1     AVAFDLLSHKEVDPFSHLSTTTRQRIRKGVIRCILATDMSRHNEILDEFNRQVLTDLNAAWEIDPNTKKP  770
SmPDE9B-a2     AVAFDLLSHKEVDPFSHLSTTTRQRIRKGVIRCILATDMSRHNEILDEFNRQVLTDLNAAWEIDPNTKKP  768
               **********************************************************************
                                                          exon 8 (1.e8)

Smp_146120.1   TWVMNKTQKDLVMVIILKISDISNEARPLNVAGPWINRLLAEFFHQSDYEKLVGLPVAPFMDRHKVTKSA  840
Smp_146120.2   TWVMNKTQKDLVMVIILKISDISNEARPLNVAGPWINRLLAEFFHQSDYEKLVGLPVAPFMDRHKVTKSA  833
SmPDE9B-a1     TWVMNKTQKDLVMVIILKISDISNEARPLNVAGPWINRLLAEFFHQSDYEKLVGLPVAPFMDRHKVTKSA  840
SmPDE9B-a2     TWVMNKTQKDLVMVIILKISDISNEARPLNVAGPWINRLLAEFFHQSDYEKLVGLPVAPFMDRHKVTKSA  838
               **********************************************************************
                                                                   exon 9 (1.e9)

Smp_146120.1   SQCGFIRFVILPLFESLAKLLPEVKPIIVQPALEQLAYYTDLHNNEEKKTNTENQKSNTNEHQNGNNNNH  910
Smp_146120.2   SQCGFIRFVILPLFESLAKLLPEVKPIIVQPALEQLAYYTDLHNNEEKKTNTENQKSNTNEHQNGNNNNH  903
SmPDE9B-a1     SQCGFIRFVILPLFESLAKLLPEVKPIIVQPALEQLAYYTDLHNNEEKKTNTENQKSNTNEHQNGNNNNH  910
SmPDE9B-a2     SQCGFIRFVILPLFESLAKLLPEVKPIIVQPALEQLAYYTDLHNNEEKKTNTENQKSNTNEHQNGNNNNH  908
               **********************************************************************
                                                       exon 10 (1.e10)

Smp_146120.1   NEKEHSK  917
Smp_146120.2   NEKEHSK  910
SmPDE9B-a1     NEKEHSK  917
SmPDE9B-a2     NEKEHSK  915
               *******
```

SmPDE9C

```
               ▲
Smp_342020.1   -MFKRLIRCHVKSSRTPPNKDGTNNKIHLPTKCTTWLFSTISSSSKISTTSDIEASKSTETCLVDNSSKTNNCNETCNLI  79
Smp_342020.2   -MFKRLIRCHVKSSRTPPNKDGTNNKIHLPTKCTTWLFSTISSSSKISTTSDIEASKSTETCLVDNSSKTNNCNETCNLI  79
SmPDE9C        MMFKRLIRCHVKSSRTPPNKDGTNNKIHLPTKCTTWLFSTISSSSKISTTSDIEASKSTETCLVDNSSKTNNCNETCNLI  80
                *******************************************************************************
encoded by:                                  exon 1 (1.e2/9C.e2)

Smp_342020.1   NTQHPNDSQRISCNPYCPDPEGIKLPFIHFTKVRNQFLAIRSQSISSSIKEQLKSHSFNNWLYSDAELINFVKFMFVDLN  159
Smp_342020.2   NTQHPNDSQRISCNPYCPDPEGIKLPFIHFTKVRNQFLAIRSQSISSSIKEQLKSHSFNNWLYSDAELINFVKFMFVDLN  159
SmPDE9C        NTQHPNDSQRISCNPYCPDPEGIKLPFIHFTKVRNQFLAIRSQSISSSIKEQLKSHSFNNWLYSDAELINFVKFMFVDLN  160
               ********************************************************************************
                                                                 exon 2 (1.e3/9C.e3)

Smp_342020.1   LPELCHFSIDTLENWIFSTYSRYNNVPFHNFKHAFMVTQMVSIIIIITIIIIIMSNLKFTKSLMYCIIKMVNLPLYLSSV  239
Smp_342020.2   LPELCHFSIDTLENWIFSTYSRYNNVPFHNFKHAFMVTQMVSIIIIITIIIIIMSNLKFTKSLMYCIIKMVNLPLYLSSV  239
SmPDE9C        LPELCHFSIDTLENWIFSTYSRYNNVPFHNFKHAFMVTQM-----------------------MYCIIKMVNLPLYLSSV  217
               ****************************************                       *****************

Smp_342020.1   DLLILLFSALSHDLDHPGFTNSYQINSGTWLALRYNDISPLENHHCMTAFDLITNNPTANIISGLTPNESRHFRRSVIRC  319
Smp_342020.2   DLLILLFSALSHDLDHPGFTNSYQINSGTWLALRYNDISPLENHHCMTAFDLITNNPTANIISGLTPNESRHFRRSVIRC  319
SmPDE9C        DLLILLFSALSHDLDHPGFTNSYQINSGTWLALRYNDISPLENHHCMTAFDLITNNPTANIISGLTPNESRHFRRSVIRC  297
               ********************************************************************************
                exon 3 (1.e4)                              exon 4 (1.e5)

Smp_342020.1   ILSTDMAIHSECLSQFQVLRKQVYLNCQSLSIDMSSSSISSIKHNHQNIDHSCCNNNNQQKKLKNPDSIFPSLQLSSSYI  399
Smp_342020.2   ILSTDMAIHSECLSQFQVLRKQVYLNCQSLSIDMSSSSISSIKHNHQNIDHSCCNNNNQQKKLKNPDSIFPSLQLSSSYI  399
SmPDE9C        ILSTDMAIHSECLSQFQVLRKQVYLNCQSLSIDMSSSSISSIKHNHQNIDHSCCNNNNQQKKLKNPDSIFPSLQLSSSYI  377
               ********************************************************************************
                                                  exon 5 (1.e6)

                                                   156aa-insert
Smp_342020.1   GYSSPSPTQQQPTLTTSHDHYHRHQVNHNDNDETGVDRQNRSEKYEQYNSCINQHVNNNNNNVVNFGGDNDSDHNGDDIN  479
Smp_342020.2   GYSSPSPTQQQPTLTTSHDHYHRHQVNHNDNDETGVDRQNRSEKYEQYNSCINQHVNNNNNNVVNFGGDNDSDHNGDDIN  479
SmPDE9C        GYSSPSPTQQQPTLTTSHDHYHRHQVNHNDNDETGVDRQNRSEKYEQYNSCINQHVNNNNNNVVNFGGDNDSDHNGDDIN  457
               ********************************************************************************

Smp_342020.1   NNDYGHINSSLIQSSLISLINQEPEYLLRLLMILLKVCDISNEIRSPLVADAWVDCLFNEFFLQAAAEKQAGLPVAPHMD  559
Smp_342020.2   NNDYGHINSSLIQSSLISLINQEPEYLLRLLMILLKVCDISNEIRSPLVADAWVDCLFNEFFLQAAAEKQAGLPVAPHMD  559
SmPDE9C        NNDYGHINSSLIQSSLISLINQEPEYLLRLLMILLKVCDISNEIRSPLVADAWVDCLFNEFFLQAAAEKQAGLPVAPHMD  537
               ********************************************************************************

Smp_342020.1   PDLVVKSNSQLNFLHSILIPLVKELTYIFRELHVLLESAHRRSEHFFQIKQYELAQQVVDSNCCSTTVTTSTSSTLPITT  639
Smp_342020.2   PDLVVKSNSQLNFLHSILIPLVKELTYIFRELHVLLESAHRRSEHFFQIKQYELAQQVVDSNCCSTTVTTSTSSTLPITT  639
SmPDE9C        PDLVVKSNSQLNFLHSILIPLVKELTYIFRELHVLLESAHRRSEHFFQIKQYELAQQVVDSNCCSTTVTTSTSSTLPITT  617
               ********************************************************************************
                  exon 6 (1.e7)                           exon 7 (1.e8/2.e8/9C.e8)

Smp_342020.1   ITSTSVCHVNKSN--ATEKNINCVQSALCPGEITLDANENLIQKQQQPHSRHESPTIHTHTGHLYSPISQSSSSTLHCKS  717
Smp_342020.2   ITSTSVCHVNKSNVRATEKNINCVQSALCPGEITLDANENLIQKQQQPHSRHESPTIHTHTGHLYSPISQSSSSTLHCKS  719
SmPDE9C        ITSTSVCHVNKSNVRVSLLLLLFN--------------------------------------------------------  641
               *************   :   :   ********************************************************
                                                          exon 8 (1.e9)

Smp_342020.1   SPIKVQQSQVPHCVSVESPYLKKQEYASFTSDVYNDTTH  756
Smp_342020.2   SPIKVQQSQVPHCVSVESPYLKKQEYASFTSDVYNDTTH  758
SmPDE9C        ---------------------------------------  641
               ***************************************
```

SmPDE11

```
                  o                        o
Smp_179590.1   MSSLVRMCELCGGHIGEQSELSFEDMVTNWLDENPEFTFKYFVKSASPSMVEAWANGRNHGEYDCLFDNS  70
Smp_179590.2   MSSLVRMCELCGGHIGEQSELSFEDMVTNWLDENPEFTFKYFVKSASPSMVEAWANGRNHGEYDCLFDNS  70
SmPDE11-a1     MSSLVRMCELCGGHIGEQSELSFEDMVTNWLDENPEFTFKYFVKSASPSMVEAWANGRNHGEYDCLFDNS  70
SmPDE11-a2     MSSLVGMCELCGGHIGEQSELSFEDMITNWLDENPEFTFKYFVKSASPSMVEAWANGRNHGEYDCLFDNS  70
               ***** ********************:*******************************************
encoded by:                            exon 1 (1.e1/11-a2.e1)

Smp_179590.1   ITVMDEHNDKNDDKTPVTTSLSLPIRKISSQDLELTYDKRILSSNEDGKPTFINSVFFPFPSNEHIDSNT  140
Smp_179590.2   ITVMDEHNDKNDDKTPVTTSLSLPIRKISSQDLELTYDKRILSSNEDGKPTFINSVFFPFPSNEHIDSNT  140
SmPDE11-a1     ITVMDEHNDKNDDKTPVTTSLSLPIRKISSQDLELTYDKRILSSNEDGKPTFINSVFFPFPSNEHIDSNT  140
SmPDE11-a2     ITVMDEHNDKNDDKTPVTTSLSLPIRKISSQDLELTYDKRILSSNEDGKPTFINSVFFPFPSNEHIDSNT  140
               **********************************************************************

Smp_179590.1   SSVRQTVASPSRPTHLTERDLISELALDICRELDVTSLSFKIVQNVCRLINADRGSFFLVEKSRSTGEDV  210
Smp_179590.2   SSVRQTVASPSRPTHLTERDLISELALDICRELDVTSLSFKIVQNVCRLINADRGSFFLVEKSRSTGEDV  210
SmPDE11-a1     SSVRQTVASPSRPTHLTERDLISELALDICRELDVTSLSFKIVQNVCRLINADRGSFFLVEKSRSTGEDV  210
SmPDE11-a2     SSVRQTVASPSRPTHLTERDLISELALDICRELDVTSLSFKIVQNVCRLINADRGSFFLVEKSRSTGEDV  210
               **********************************************************************

Smp_179590.1   LVSKLFDITPECIFDDVLQRCSSNHIIVPFNVGVTGYVARTGDYANIPDAYADPRFDDSVDRVTGYKTRC  280
Smp_179590.2   LVSKLFDITPECIFDDVLQRCSSNHIIVPFNVGVTGYVARTGDYANIPDAYADPRFDDSVDRVTGYKTRC  280
SmPDE11-a1     LVSKLFDITPECIFDDVLQRCSSNHIIVPFNVGVTGYVARTGDYANIPDAYADPRFDDSVDRVTGYKTRC  280
SmPDE11-a2     LVSKLFDITPECIFDDVLQRCSSNHIIVPFNVGVTGYVARTGDYANIPDAYADPRFDDSVDRVTGYKTRC  280
               **********************************************************************

Smp_179590.1   LLCMPIKNVDGKVLGVALVINKKVPSDQHHDQSINSVQPSSCESESISKHASFTEEDVKIFQSYVTFCGI  350
Smp_179590.2   LLCMPIKNVDGKVLGVALVINKKVPSDQHHDQSINSVQPSSCESESISKHASFTEEDVKIFQSYVTFCGI  350
SmPDE11-a1     LLCMPIKNVDGKVLGVALVINKKVPSDQHHDQSINSVQPSSCESESISKHASFTEEDVKIFQSYVTFCGI  350
SmPDE11-a2     LLCMPIKNVDGKVLGVALVINKKVPSDQHHDQSINSVQPSSCESESISKHASFTEEDVKIFQSYVTFCGI  350
               **********************************************************************
                                          exon 2 (1.e2)

Smp_179590.1   GLHNAQIYEQSRLETYRNQVLLELARIIFSEQLDITRLIYSVLSHTICLLQCQRCQLLLVKTTSSMSSYS  420
Smp_179590.2   GLHNAQIYEQSRLETYRNQVLLELARIIFSEQLDITRLIYSVLSHTICLLQCQRCQLLLVKTTSSMSSYS  420
SmPDE11-a1     GLHNAQIYEQSRLETYRNQVLLELARIIFSEQLDITRLIYSVLSHTICLLQCQRCQLLLVKTTSSMSSYS  420
SmPDE11-a2     GLHNAQIYEQSRLETYRNQVLLELARIIFSEQLDITRLIYSVLSHTICLLQCQRCQLLLVKTTSSMSSYS  420
               **********************************************************************
                                                      exon 3 (1.e3)

Smp_179590.1   SIDEMGPFHDHFSQIFELAWNEKSDSPDVKKKKHSIDEARFPVQLDLAIHVLQTGESLHVNINGTTTISN  490
Smp_179590.2   SIDEMGPFHDHFSQIFELAWNEKSDSPDVKKKKHSIDEARFPVQLDLAIHVLQTGESLHVNINGTTTISN  490
SmPDE11-a1     SIDEMGPFHDHFSQIFELAWNEKSDSPDVKKKKHSIDEARFPVQLDLAIHVLQTGESLHVNINGTTTISN  490
SmPDE11-a2     SIDEMGPFHDHFSQIFELAWNEKSDSPDVKKKKHSIDEARFPVQLDLAIHVLQTGESLHVNINGTTTISN  490
               **********************************************************************
                             exon 4 (1.e4)             exon 5 (1.e5)

                o
Smp_179590.1   NNNKNKNNNNEYKKIDETLEEDLDPVWRSRSVLCMPIKHSDGKVLAVCIITNKSTVDLRINNNNFSQQVT  560
Smp_179590.2   NNNKNKNNNNEYKKIDETLEEDLDPVWRSRSVLCMPIKHSDGKVLAVCIITNKSTVDLRINNNNFSQQVT  560
SmPDE11-a1     NNNKNKNNNNEYKKIDETLEEDLDPVWRSRSVLCMPIKHSDGKVLAVCIITNKSTVDLRINNNNFSQQVT  560
SmPDE11-a2     NYNKNKNNNNEYKKIDETLEEDLDPVWRSRSVLCMPIKHSDGKVLAVCIITNKSTVDLRINNNNFSQQVT  560
               * ********************************************************************
             exon 6 (1.e6/11-a2.e6)                    exon 7 (1.e7)
```

```
Smp_179590.1   RHFDFKPVTDNVQCSSNDNLSSKEPVTMSTSINDWSGIFTYSDEFLFEAFALFVGLGISNSQLYEKAIRS  630
Smp_179590.2   RHFDFKPVTDNVQCSSNDNLSSKEPVTMSTSINDWSGIFTYSDEFLFEAFALFVGLGISNSQLYEKAIRS  630
SmPDE11-a1     RHFDFKPVTDNVQCSSNDNLSSKEPVTMSTSINDWSGIFTYSDEFLFEAFALFVGLGISNSQLYEKAIRS  630
SmPDE11-a2     RHFDFKPVTDNVQCSSNDNLSSKEPVTMSTSINDWSGIFTYSDEFLFEAFALFVGLGISNSQLYEKAIRS  630
               **********************************************************************

Smp_179590.1   AAKQKVIMDVLSYHATAPTSEAKRLATSLIPTMRFYHLDKFSFTDVRLSDEDTLKACIRMFQEMNFMKSI  700
Smp_179590.2   AAKQKVIMDVLSYHATAPTSEAKRLATSLIPTMRFYHLDKFSFTDVRLSDEDTLKACIRMFQEMNFMKSI  700
SmPDE11-a1     AAKQKVIMDVLSYHATAPTSEAKRLATSLIPTMRFYHLDKFSFTDVRLSDEDTLKACIRMFQEMNFMKSI  700
SmPDE11-a2     AAKQKVIMDVLSYHATAPTSEAKRLATSLIPTMRFYHLDKFSFTDVRLSDEDTLKACIRMFQEMNFMKSI  700
               **********************************************************************

Smp_179590.1   HFDQLSFARWLLSVRKNYREVTYHNWRHAFNVTQTMFCILLKGDFQSVFTDLECLALLTACLSHDIDHRG  770
Smp_179590.2   HFDQLSFARWLLSVRKNYREVTYHNWRHAFNVTQTMFCILLKGDFQSVFTDLECLALLTACLSHDIDHRG  770
SmPDE11-a1     HFDQLSFARWLLSVRKNYREVTYHNWRHAFNVTQTMFCILLKGDFQSVFTDLECLALLTACLSHDIDHRG  770
SmPDE11-a2     HFDQLSFARWLLSVRKNYREVTYHNWRHAFNVTQTMFCILLKGDFQSVFTDLECLALLTACLSHDIDHRG  770
               **********************************************************************
                         exon 8 (1.e8)

Smp_179590.1   TDNQFQIKTMSPLAKLYSTSVLEHHHFNQFMMILSIKGNNFLCNLNRSEYDTVVKLIREAILATDLSRYF  840
Smp_179590.2   TDNQFQIKTMSPLAKLYSTSVLEHHHFNQFMMILSIK---------------------------------  807
SmPDE11-a1     TDNQFQIKTMSPLAKLYSTSVLEHHHFNQFMMILSIKGNNFLCNLNRSEYDTVVKLIREAILATDLSRYF  840
SmPDE11-a2     TDNQFQIKTMSPLAKLYSTSVLEHHHFNQFMMILSIKGNNFLCNLNRSEYDTVVKLIREAILATDLSRYF  840
               **********************************************************************
                        exon 9 (1.e9)

Smp_179590.1   ARLPKFQQTLHHLKEINDQSTNLWRNDREQRLLLGCMFMTACDVSAITKPWPVQKLTAEMVANEFFEQGD  910
Smp_179590.2   --------------------------------------------------------TAEMVANEFFEQGD  821
SmPDE11-a1     ARLPKFQQTLHHLKEINDQSTNLWRNDREQRLLLGCMFMTACDVSAITKPWPVQKLTAEMVANEFFEQGD  910
SmPDE11-a2     ARLPKFQQTLHHLKEINDQSTNLWRNDREQRLLLGCMFMTACDVSAITKPWPVQKLTAEMVANEFFEQGD  910
               **********************************************************************
                                  exon 10 (1.e10)                     exon 11 (1.e11)

Smp_179590.1   LEKERLNVTPAALMDRERSNELPKLQVSFIDSICVPIYEAIVQVSPNFEPLLKGCKRNRTCWLILSENGE  980
Smp_179590.2   LEKERLNVTPAALMDRERSNELPKLQVSFIDSICVPIYEAIVQVSPNFEPLLKGCKRNRTCWLILSENGE  891
SmPDE11-a1     LEKERLNVTPAALMDRERSNELPKLQVSFIDSICVPIYEAIVQVSPNFEPLLKGCKRNRTCWLILSENGE  980
SmPDE11-a2     LEKERLNVTPAALMDRERSNELPKLQVSFIDSICVPIYEAIVQVSPNFEPLLKGCKRNRTCWLILSENGE  980
               **********************************************************************
                              exon 12 (1.e12)

                                               o
Smp_179590.1   VDHSLYGLNDESETIPGTSTETTAKSITTTTAEGSTQLENVITSTSNIITSLGTLNPDSAERIAKSKPFT  1050
Smp_179590.2   VDHSLYGLNDESETIPGTSTETTAKSITTTTAEGSTQLENVITSTSNIITSLGTLNPDSAERIAKSKPFT  961
SmPDE11-a1     VDHSLYGLNDESETIPGTSTETTAKSITTTTAEGSTQLENVITSTSNIITSLGTLNPDSAERIAKSKPFT  1050
SmPDE11-a2     VDHSLYGLNDESETIPGTSTETTAKSITTTTTEGSTQLENVITSTSNIITSLGTLNPDSAERIAKSKPFT  1050
               *******************************:**************************************
                                   exon 13 (1.e13/2.e12/11-a2.e13)

Smp_179590.1   SSNIRRASVSSMTTVGGQEPPIQCQSSVESK  1081
Smp_179590.2   SSNIRRASVSSMTTVGGQEPPIQCQSSVESK  992
SmPDE11-a1     SSNIRRASVSSMTTVGGQEPPIQCQSSVESK  1081
SmPDE11-a2     SSNIRRASVSSMTTVGGQEPPIQCQSSVESK  1081
               *******************************
```
