# Supplementary material for: Cloning and functional complementation of ten Schistosoma mansoni phosphodiesterases expressed in the mammalian host stages
Source: PLoS Negl Trop Dis. 2020 Jul 30;14(7):e0008447. doi: 10.1371/journal.pntd.0008447 (PMC7430754; doi:10.1371/journal.pntd.0008447)
Supplement: S2 Fig — (PDF) [file pntd.0008447.s002.pdf]

S2 Fig. PCR of SmPDE9C in multiple *S. mansoni* lifecycle stages

979 bp →

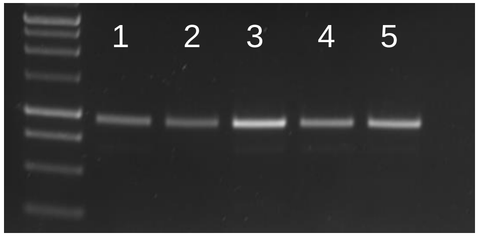

1 = schistosomulae cDNA
2 = 4 week male worm cDNA
3 = 4 week female worm cDNA
4 = adult male worm cDNA
5 = adult female worm cDNA

The single 979 bp band indicates that the Catalytic Domain insertion was found in all stages.
