# Supplementary material for: Cloning and functional complementation of ten Schistosoma mansoni phosphodiesterases expressed in the mammalian host stages
Source: PLoS Negl Trop Dis. 2020 Jul 30;14(7):e0008447. doi: 10.1371/journal.pntd.0008447 (PMC7430754; doi:10.1371/journal.pntd.0008447)
Supplement: S3 Fig — (PDF) [file pntd.0008447.s003.pdf]

S3 Fig. Sequence identity in the catalytic core between *S.mansoni* and human PDEs

The catalytic core sequences (helices H3-H16) were pairwise aligned using the program needleall (EMBOSS software package version 6.5) and percentage identity scores were calculated using a script that enabled the exclusion of gaps. The H3-H16 region of each PDE sequence was adopted from several representative PDB entries. If not available, the helix boundaries were determined from sequence alignments and secondary structure predictions using the programs PredictProtein (www.predictprotein.org), JPred4 (http://www.compbio.dundee.ac.uk/jpred/) and YASPIN (http://www.ibi.vu.nl/programs/yaspinwww/).
Residues of hPDEs are numbered according to the canonical UniProt sequences (e.g PDE1A_HUMAN).

	H3-H16	1<br>285-602	2*<br>620-906	4A<br>373-666	4B<br>716-1008	4C<br>389-683	7var<br>128-422	8<br>201-532	9A<br>213-511	9B<br>581-890	9C<br>144-596	11<br>679-977
**SmPDE1**	285-602	100	32	43	42	43	30	34	33	32	37	32
**SmPDE2***	620-906	32	100	36	34	34	29	32	30	32	33	39
**SmPDE4A**	373-666	43	36	100	64	51	33	36	33	34	35	37
**SmPDE4B**	716-1008	42	34	64	100	52	33	38	31	35	29	32
**SmPDE4C**	389-683	43	34	51	52	100	31	36	34	36	32	28
**SmPDE7var**	128-422	30	29	33	33	31	100	32	30	30	29	30
**SmPDE8**	201-532	34	32	36	38	36	32	100	38	32	30	32
**SmPDE9A**	213-511	33	30	33	31	34	30	38	100	46	45	33
**SmPDE9B**	581-890	32	32	34	35	36	30	32	46	100	44	31
**SmPDE9C**	144-596	37	33	35	29	32	29	30	45	44	100	31
**SmPDE11**	679-977	32	39	37	32	28	30	32	33	31	31	100
**hPDE1A**	174-521	56	36	42	38	35	30	33	31	33	35	31
**hPDE1B**	178-502	57	37	43	40	40	30	33	32	35	35	34
**hPDE1C**	183-527	58	34	42	42	33	30	30	32	33	32	34
**hPDE2A**	614-899	30	58	31	30	29	27	30	31	32	35	43
**hPDE3A**	709-1088	37	36	37	37	36	36	36	30	35	33	35
**hPDE3B**	694-1074	35	33	40	39	38	36	35	28	36	34	35
**hPDE4A**	389-683	42	36	70	68	52	31	38	31	38	28	34
**hPDE4B**	362-655	42	37	69	67	52	33	37	30	35	28	33
**hPDE4C**	344-631	43	37	67	66	51	34	38	33	36	31	35
**hPDE4D**	418-711	43	35	69	68	52	34	36	31	37	28	34
**hPDE5A**	567-858	29	42	31	31	30	31	28	35	31	34	51
**hPDE6A**	514-814	30	38	30	32	31	30	29	33	28	27	42
**hPDE6B**	512-812	30	36	31	31	31	33	28	33	30	23	43
**hPDE6C**	517-817	30	35	33	32	29	31	29	32	29	24	43
**hPDE7A**	168-454	35	33	35	36	32	42	36	33	35	34	32
**hPDE7B**	129-416	35	29	36	37	32	38	32	30	31	31	29
**hPDE8A**	512-816	35	33	38	38	39	35	55	37	32	30	29
**hPDE8B**	571-871	33	30	39	37	36	31	52	37	30	30	26
**hPDE9A**	267-563	35	30	34	31	34	31	38	65	47	44	29
**hPDE10A**	475-757	25	40	30	28	30	31	26	32	28	31	46
**hPDE11**	619-911	30	39	36	30	25	30	28	33	30	32	48
**TbrPDEB1**	623-917	35	31	35	35	32	33	33	35	31	31	41
**TcrPDEC1**	326-611	32	28	32	32	30	26	33	27	29	30	27
**LmjPDEB1**	635-930	29	32	37	33	32	34	32	33	33	30	37

color coding:

25	75	100

* Smp_135500.1 (WBPS14)
