# Supplementary material for: Cloning and functional complementation of ten Schistosoma mansoni phosphodiesterases expressed in the mammalian host stages
Source: PLoS Negl Trop Dis. 2020 Jul 30;14(7):e0008447. doi: 10.1371/journal.pntd.0008447 (PMC7430754; doi:10.1371/journal.pntd.0008447)
Supplement: S4 Fig — (PDF) [file pntd.0008447.s004.pdf]

S4 Fig. PCRs for Sm4A complementation

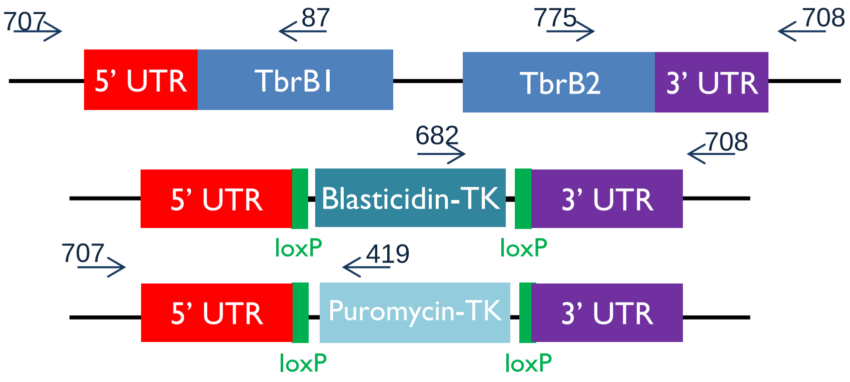

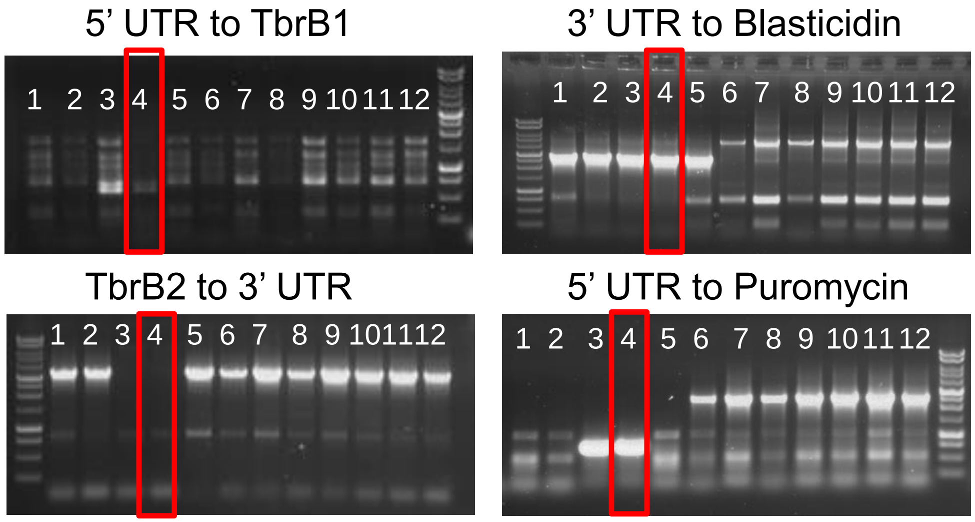

1 = B1/2 sKO
2 = B1/2 sKO + Flag-Sm4A-GFP
3 = B1/2 dKO ? + Flag-Sm4A-GFP cl. 1-2
4 = B1/2 dKO ? + Flag-Sm4A-GFP cl. 1-3
5 = B1/2 dKO ? + Flag-Sm4A-GFP cl. 2-1
6 = B1/2 dKO ? + Flag-Sm4A-GFP cl. 2-2
7 = B1/2 dKO ? + Flag-Sm4A-GFP cl. 2-3
8 = B1/2 dKO ? + Flag-Sm4A-GFP cl. 2-4
9 = B1/2 dKO ? + Flag-Sm4A-GFP cl. 2-5
10 = B1/2 dKO ? + Flag-Sm4A-GFP cl. 2-6
11 = B1/2 dKO ? + Flag-Sm4A-GFP cl. 2-7
12 = B1/2 dKO ? + Flag-Sm4A-GFP cl. 2-8
