# Supplementary material for: Cloning and functional complementation of ten Schistosoma mansoni phosphodiesterases expressed in the mammalian host stages
Source: PLoS Negl Trop Dis. 2020 Jul 30;14(7):e0008447. doi: 10.1371/journal.pntd.0008447 (PMC7430754; doi:10.1371/journal.pntd.0008447)
Supplement: S5 Fig — (PDF) [file pntd.0008447.s005.pdf]

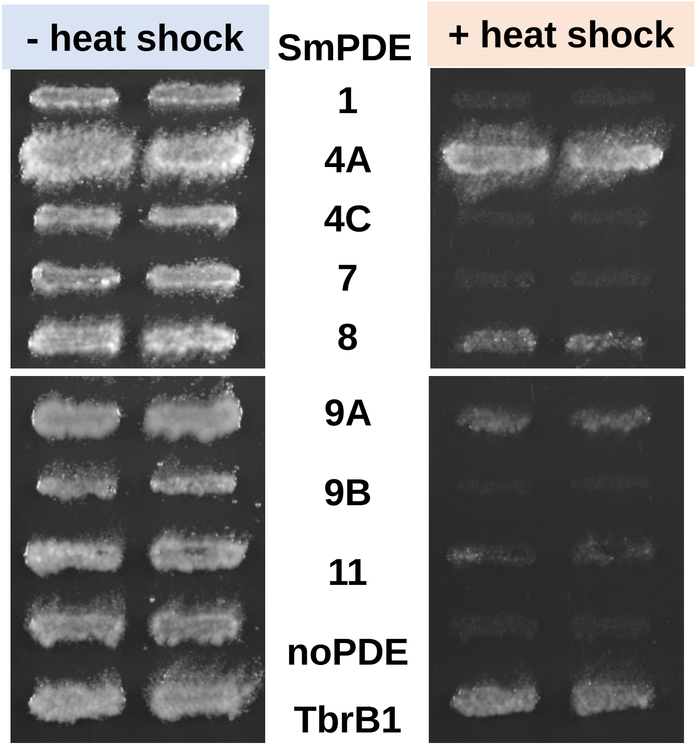

S5 Fig. **Complementation temperature sensitivity by SmPDEs**. Temperature sensitivity of the *pde 1/2*-deletion strain PM943 (**W303Ia genetic background; Ref Ma et al. Molecular Biology of the Cell Vol. 10, 91–104, January 1999)** is complemented to various extents by SmPDE4a, SmPDE8, SmPDE9a and SmPDE11, respectively. PM943 transformed with TbrPDEB1 was taken along as positive control. Duplicate patches of recombinant yeast strains were subjected to heat shock (55°C for 15 min) or control conditions (30°C) and were then grown at 30°C for 2 days. **Complementation by SmPDE1 is less evident under these conditions in comparison with complementation in the PP5 genetic background.**
