# Supplementary material for: Cloning and functional complementation of ten Schistosoma mansoni phosphodiesterases expressed in the mammalian host stages
Source: PLoS Negl Trop Dis. 2020 Jul 30;14(7):e0008447. doi: 10.1371/journal.pntd.0008447 (PMC7430754; doi:10.1371/journal.pntd.0008447)
Supplement: S1 Table — (PDF) [file pntd.0008447.s006.pdf]

S1Table: List of plasmids used for knockouts and expression in *T. brucei*

Plasmid	Use
pHDK76	Knock out construct TbrB1-B2 – Blasticidin-HSVTK
pHDK79	$pRPa^{iB1-6Myc}$ – tet-inducible expression of TbrB1 from rRNA locus, 6Myc tag
pHDK80	$pRPa^{iB2-6Myc}$ – tet-inducible expression of TbrB2 from rRNA locus, 6Myc tag
pHDK82	Knock out construct TbrB1-B2 – Puromycin-HSVTK
pHDK133	$pRPa^{i210B1-Sm4A-GFP}$ – tet-inducible expression of Sm4A from rRNA locus, GFP tag
pHDK194	$pRPa^{i210B1-Sm4B-GFP}$ – tet-inducible expression of Sm4B from rRNA locus, GFP tag
pHDK211	$pRPa^{i210B1-Sm8F-GFP}$ – tet-inducible expression of Sm8-full length from rRNA locus, GFP tag
pHDK212	$pRPa^{i210B1-Sm8LD-GFP}$ – tet-inducible expression of Sm8-loop deleted from rRNA locus, GFP tag
pHDK232	$pRPa^{i210B1-Sm11-GFP}$ – tet-inducible expression of Sm11 from rRNA locus, GFP tag
pHDK233	$pRPa^{i210B1-Sm9A-GFP}$ – tet-inducible expression of Sm9A from rRNA locus, GFP tag
pHDK241	$pRPa^{i210B1-Sm1-GFP}$ – tet-inducible expression of Sm1 from rRNA locus, GFP tag
