# Supplementary material for: Cloning and functional complementation of ten Schistosoma mansoni phosphodiesterases expressed in the mammalian host stages
Source: PLoS Negl Trop Dis. 2020 Jul 30;14(7):e0008447. doi: 10.1371/journal.pntd.0008447 (PMC7430754; doi:10.1371/journal.pntd.0008447)
Supplement: S2 Table — (PDF) [file pntd.0008447.s007.pdf]

S2 Table. Antischistosomal effects of PDE inhibitors

Effect of NPD001 on mature worms

Schistosoma mansoni mature (6 weeks) worm killing and ovipositing under different concentrations of NPD-001 compared to positive and negative controls.

Test cpds	Dose	End of observation (5 days)					Notes
		% Worm killing			Ovipositing		
		Total worms	Male ♂	Female ♀	No. of ova/couple	% reduction	
Untreated control		0/17 (0%)	0/9 (0%)	0/8 (0%)	155	-	Coupling
Positive control (PZQ)	100 µM	14/14 (100%)	7/7 (100%)	7/7 (100%)	0	100%	-
	50 µM	13/13 (100%)	7/7 (100%)	6/6 (100%)	0	100%	-
	25 µM	15/15 (100%)	9/9 (100%)	6/6 (100%)	0	100%	-
	10 µM	15/15 (100%)	8/8 (100%)	7/7 (100%)	0	100%	-
	5 µM	13/13 (100%)	7/7 (100%)	6/6 (100%)	0	100%	-
Negative control (DMSO)	DMSO=2%	0/15 (0%)	0/7 (0%)	0/8 (0%)	145	7%	Coupling
	DMSO=1%	0/15 (0%)	0/9 (0%)	0/6 (0%)	150	3%	Coupling
	DMSO=0.5%	0/13 (0%)	0/7 (0%)	0/6 (0%)	157	0%	Coupling
	DMSO=0.25 %	0/13 (0%)	0/7 (0%)	0/6 (0%)	142	8%	Coupling
	DMSO=0.125 %	0/12 (0%)	0/6 (0%)	0/6 (0%)	140	10%	Coupling
**NPD-001** Batch IV	100 µM	5/15 (33%)	5/8 (63%)	0/7 (0%)	0	100%	Uncoupling, sluggish male
	50 µM	0/17 (0%)	0/10 (0%)	0/7 (0%)	120	23%	Coupling
	25 µM	0/16 (0%)	0/8 (0%)	0/8 (0%)	135	13%	Coupling
	10 µM	0/14 (0%)	0/8 (0%)	0/6 (0%)	170	0%	Coupling
	5 µM	0/13 (0%)	0/8 (0%)	0/5 (0%)	160	0%	Coupling

NB: Stock solution 5mM of test cpd dissolved in 1ml DMSO and then diluted to different concentrations with media.

NPD-001 showed sluggish worm movement, 33% worm killing and uncoupling with no ova at 100µM and trivial reduction in number of ova with 50µM and 25µM. No reduction in number of ova was recorded with the lower concentrations of 10µM and 5µM.

Effect of NPD010 (trequinsin) on mature worms

Schistosoma mansoni **mature (6 weeks) worm killing and ovipositing under different concentrations of NPD-010 test compound compared to positive and negative controls.**

Test cpds	Dose	End of observation (5 days)					Notes
		% Worm killing			Ovipositing		
		Total worms	Male ♂	Female ♀	No. of ova/couple	% reduction	
Untreated control		0/15 (0%)	0/9 (0%)	0/6 (0%)	140	-	Intact couples
Positive control (PZQ)	100 µM	13/13 (100%)	6/6 (100%)	7/7 (100%)	0	100%	-
	50 µM	15/15 (100%)	8/8 (100%)	7/7 (100%)	0	100%	-
	25 µM	15/15 (100%)	9/9 (100%)	6/6 (100%)	0	100%	-
	10 µM	14/14 (100%)	8/8 (100%)	6/6 (100%)	0	100%	-
	5 µM	14/14 (100%)	7/7 (100%)	7/7 (100%)	0	100%	-
Negative control (DMSO)	DMSO=2%	0/14 (0%)	0/6 (0%)	0/8 (0%)	125	11%	Intact couples
	DMSO=1%	0/16 (0%)	0/10 (0%)	0/6 (0%)	130	7%	Intact couples
	DMSO=0.5%	0/14 (0%)	0/8 (0%)	0/6 (0%)	125	11%	Intact couples
	DMSO=0.25%	0/12 (0%)	0/6 (0%)	0/6 (0%)	130	7%	Intact couples
	DMSO=0.125%	0/14 (0%)	0/8 (0%)	0/6 (0%)	120	14%	Intact couples
NPD-010	100 µM	0/15 (0%)	0/9 (0%)	0/6 (0%)	0	100%	Uncoupling
	50 µM	0/12 (0%)	0/6 (0%)	0/6 (0%)	0	100%	Uncoupling
	25 µM	0/16 (0%)	0/10 (0%)	0/6 (0%)	100	29%	Intact couples
	10 µM	0/16 (0%)	0/9 (0%)	0/7 (0%)	110	21%	Intact couples
	5 µM	0/14 (0%)	0/8 (0%)	0/6 (0%)	100	29%	Intact couples

NPD-010 did not show any worm killing; the compound showed uncoupling at 100 and 50µM with a slight reduction in the number of ova with lower concentrations.

Effect of NPD029 on mature worms

Schistosoma mansoni mature (6 weeks) worm killing and ovipositing under different concentrations of NPD-0000029 compared to positive and negative controls.

Test cpds	Dose	End of observation (5 days)					Notes
		% Worm killing			Ovipositing		
		Total worms	Male ♂	Female ♀	No. of ova/couple	% reduction	
Untreated control		0/14 (0%)	0/8 (0%)	0/6 (0%)	150	-	Coupling
Positive control (PZQ)	100 µM	13/13 (100%)	7/7 (100%)	6/6 (100%)	0	100%	-
	50 µM	14/14 (100%)	8/8 (100%)	6/6 (100%)	0	100%	-
	25 µM	14/14 (100%)	8/8 (100%)	6/6 (100%)	0	100%	-
	10 µM	13/13 (100%)	7/7 (100%)	6/6 (100%)	0	100%	-
	5 µM	12/12 (100%)	6/6 (100%)	6/6 (100%)	0	100%	-
Negative control (DMSO)	DMSO=2%	0/15 (0%)	0/7 (0%)	0/8 (0%)	135	10%	Coupling
	DMSO=1%	0/15 (0%)	0/8 (0%)	0/7 (0%)	140	7%	Coupling
	DMSO=0.5%	0/14 (0%)	0/7 (0%)	0/7 (0%)	125	17%	Coupling
	DMSO=0.25 %	0/13 (0%)	0/7 (0%)	0/6 (0%)	130	13%	Coupling
	DMSO=0.125 %	0/14 (0%)	0/8 (0%)	0/6 (0%)	140	7%	Coupling
NPD-029	100 µM	0/15 (0%)	0/9 (100%)	0/6 (0%)	0	100%	Uncoupling, sluggish male
	50 µM	0/14 (0%)	0/9 (0%)	0/5 (0%)	90	40%	Sluggish male, Uncoupling on 3rd Day
	25 µM	0/18 (0%)	0/10 (0%)	0/8 (0%)	100	33%	Uncoupling on 3rd Day
	10 µM	0/18 (0%)	0/10 (0%)	0/8 (0%)	160	0%	Coupling
	5 µM	0/16 (0%)	0/10 (0%)	0/6 (0%)	150	0%	Coupling

NPD-029 did not show any worm killing, even with the highest concentration tested. NPD-029 revealed only sluggish male worm movement with the concentration of 100 µM, 50 µM with a delayed effect on coupling that was recorded 3 days after addition of the compound with the concentrations of 50 µM and 25 µM.

Effect of NPD0547 on mature worms

Schistosoma mansoni mature (6 weeks) worm killing and ovipositing under different concentrations of NPD-0547 compared to positive and negative controls.

Test cpds	Dose	End of observation (5 days)					Notes
		% Worm killing			Ovipositing		
		Total worms	Male ♂	Female ♀	No. of ova/couple	% reduction	
Untreated control		0/15 (0%)	0/8 (0%)	0/7 (0%)	110	-	Coupling
Positive control (PZQ)	100 µM	13/13 (100%)	6/6 (100%)	7/7 (100%)	0	100%	-
	50 µM	15/15 (100%)	8/8 (100%)	7/7 (100%)	0	100%	-
	25 µM	15/15 (100%)	9/9 (100%)	6/6 (100%)	0	100%	-
	10 µM	14/14 (100%)	8/8 (100%)	6/6 (100%)	0	100%	-
	5 µM	14/14 (100%)	7/7 (100%)	7/7 (100%)	0	100%	-
Negative control (DMSO)	DMSO=2%	0/13 (0%)	0/7 (0%)	0/6 (0%)	105	5%	Coupling
	DMSO=1%	0/14 (0%)	0/8 (0%)	0/6 (0%)	100	9%	Coupling
	DMSO=0.5%	0/13 (0%)	0/7 (0%)	0/6 (0%)	105	5%	Coupling
	DMSO=0.25 %	0/12 (0%)	0/6 (0%)	0/6 (0%)	110	0%	Coupling
	DMSO=0.125 %	0/11 (0%)	0/6 (0%)	0/5 (0%)	100	9%	Coupling
NPD-0547	100 µM	1/13 (8%)	1/7 (14%)	0/6 (0%)	0	100%	Uncoupling, exaggerated Spastic contractions
	50 µM	0/13 (0%)	0/6 (0%)	0/7 (0%)	0	100%	Uncoupling, exaggerated Spastic contractions
	25 µM	0/11 (0%)	0/6 (0%)	0/5 (0%)	105	5%	Coupling
	10 µM	0/12 (0%)	0/6 (0%)	0/6 (0%)	110	0%	Coupling
	5 µM	0/12 (0%)	0/6 (0%)	0/6 (0%)	100	9%	Coupling

NPD-0547 showed exaggerated spastic contraction with 8% worm killing, uncoupling with no ova at 100 and 50 µM and a trivial reduction in the number of ova at concentrations of 25 and 5µM.

Effect of NPD0226 on mature worms

Schistosoma mansoni mature (6 wks old) worm killing and ovipositing under 100 µM concentration of NPD-0226 compared to positive and negative controls.

Test cpds	Dose	End of observation (5 days)					Notes
		% Worm killing			Ovipositing		
		Total worms	Male ♂	Female ♀	No. of ova/couple	% reduction	
Untreated control		0/15 (0%)	0/8 (0%)	0/7 (0%)	120		Intact couples
Positive control (PZQ)	100 µM	14/14 (100%)	7/7(100%)	7/7 (100%)	0	100%	-
Negative control (DMSO)	DMSO=2%	0/15 (0%)	0/8 (0%)	0/7 (0%)	100	17%	Intact couples
NPD-0226	100 µM	0/15 (0%)	0/8 (0%)	0/7 (0%)	40	67%	Intact couples exaggerated spastic contraction

Effect of NPD0356 on mature worms

***Schistosoma mansoni* worm (6 weeks old) killing and ovipositing under different concentrations of NPD-0356 compared to positive and negative controls.**

Test cpds	Dose	End of observation (5 days)					Notes
		% Worm killing			Ovipositing		
		Total worms	Male ♂	Female ♀	No. of ova/couple	% reduction	
Untreated control	(a)<br>(b)	0/15 (0%)<br>0/12 (0%)	0/8 (0%)<br>0/6 (0%)	0/7 (0%)<br>0/6 (0%)	125<br>100	-<br>-	Intact couples
Positive control (PZQ)	100 µM	13/13 (100%)	6/6 (100%)	7/7 (100%)	0	100%	-
	50 µM	15/15 (100%)	8/8 (100%)	7/7 (100%)	0	100%	-
	25 µM	15/15 (100%)	9/9 (100%)	6/6 (100%)	0	100%	-
	10 µM	14/14 (100%)	8/8 (100%)	6/6 (100%)	0	100%	-
	5 µM	14/14 (100%)	7/7 (100%)	7/7 (100%)	0	100%	-
Negative control (DMSO)	DMSO=2%(a)<br>DMSO=2%(b)	0/14 (0%)<br>0/15 (0%)	0/7 (0%)<br>0/9 (0%)	0/7 (0%)<br>0/6 (0%)	125<br>95	0%<br>5%	Intact couples
	DMSO=1%(a)<br>DMSO=1%(b)	0/15 (0%)<br>0/14 (0%)	0/9 (0%)<br>0/7 (0%)	0/6 (0%)<br>0/7 (0%)	130<br>90	0%<br>10%	Intact couples
	DMSO=0.5%(a)<br>DMSO=0.5%(b)	0/14 (0%)<br>0/13 (0%)	0/7 (0%)<br>0/7 (0%)	0/7 (0%)<br>0/6 (0%)	125<br>100	0%<br>0%	Intact couples
	DMSO=0.25%(a)<br>DMSO=0.25%(b)	0/13 (0%)<br>0/14 (0%)	0/7 (0%)<br>0/8 (0%)	0/6 (0%)<br>0/6 (0%)	130<br>105	0%<br>0%	Intact couples
	DMSO=0.125%(a)<br>DMSO=0.25%(b)	0/14 (0%)<br>0/12 (0%)	0/8 (0%)<br>0/6 (0%)	0/6 (0%)<br>0/6 (0%)	120<br>95	4%<br>5%	Intact couples
NPD-0356	100 µM (a)<br>100 µM (b)	6/16 (38%)<br>7/14 (50%)	6/9 (67%)<br>7/8 (88%)	0/7 (0%)<br>0/6 (0%)	0<br>0	100%<br>100%	Uncoupling, sluggish male<br>Uncoupling,sluggish male
	50 µM (a)<br>50 µM (b)	5/14 (36%)<br>5/13 (39%)	5/7 (71%)<br>5/7 (71%)	0/7 (0%)<br>0/6 (0%)	0<br>0	100%<br>100%	Uncoupling, sluggish male<br>Uncoupling,sluggish male
	25 µM (a)<br>25 µM (b)	4/15 (27%)<br>2/14 (14%)	4/7 (57%)<br>2/7 (29%)	0/8 (0%)<br>0/7 (0%)	0<br>80	100%<br>20%	Uncoupling, sluggish male<br>Intact couples, sluggish male
	10 µM (a)<br>10 µM (b)	0/16 (0%)<br>0/14 (0%)	0/9 (0%)<br>0/7 (0%)	0/7 (0%)<br>0/7 (0%)	130<br>90	0%<br>10%	Intact couples, 50% male sluggish<br>Intact couples
	5 µM (a)<br>5 µM (b)	0/18 (0%)<br>0/14 (0%)	0/11 (0%)<br>0/7 (0%)	0/7 (0%)<br>0/7 (0%)	140<br>100	0%<br>0%	Intact couples<br>Intact couples

NPD-0356 showed worm killing, sluggish worm movement of residual worms, worm uncoupling and an absence of ova with concentrations of 100, 50 and 25 µM in two experiments (a & b).

Effect of NPD0356 on early-mature worms

***Schistosoma mansoni* early mature (4 weeks) worm killing and ovipositing under different concentrations of NPD-0356 compared to positive and negative controls.**

Test cpds	Dose	End of observation (5 days)					Notes
		% Worm killing			Ovipositing		
		Total worms	Male ♂	Female ♀	No. of ova/couple	% reduction	
Untreated control		0/14 (0%)	0/7 (0%)	0/7 (0%)	90	-	Intact couples
Positive control (PZQ)	100 µM	14/14 (100%)	7/7 (100%)	7/7 (100%)	0	100%	-
	50 µM	15/15 (100%)	8/8 (100%)	7/7 (100%)	0	100%	-
	25 µM	14/14 (100%)	7/7 (100%)	7/7 (100%)	0	100%	-
	10 µM	13/13 (100%)	7/6 (100%)	6/6 (100%)	0	100%	-
	5 µM	14/14 (100%)	7/7 (100%)	7/7 (100%)	0	100%	-
Negative control (DMSO)	DMSO=2%	0/15 (0%)	0/8 (0%)	0/7 (0%)	80	11%	Intact couples
	DMSO=1%	0/14 (0%)	0/8 (0%)	0/6 (0%)	85	6%	Intact couples
	DMSO=0.5%	0/15 (0%)	0/8 (0%)	0/7 (0%)	90	0%	Intact couples
	DMSO=0.25%	0/14 (0%)	0/7 (0%)	0/7 (0%)	95	0%	Intact couples
	DMSO=0.125%	0/13 (0%)	0/7 (0%)	0/6 (0%)	95	0%	Intact couples
NPD-0356 Batch IV	100 µM	8/16(50%)	8/8 (100%)	0/8 (0%)	0	100%	Uncoupling
	50 µM	8/16 (50%)	8/8 (100%)	0/8 (0%)	0	100%	Uncoupling
	25 µM	7/16 (44%)	7/8 (88%)	0/8 (0%)	0	100%	Uncoupling, sluggish male
	10 µM	0/16 (0%)	0/8 (0%)	0/8 (0%)	25	72%	Uncoupling
	5 µM	0/15 (0%)	0/7 (0%)	0/8 (0%)	45	50%	Intact couples

NB: Stock solution 5mM of test cpds dissolved in 1ml DMSO and then diluted to different concentrations with media.

When NPD-0356 was examined for its effect on early mature worms, there were higher reductions in worm number than that recorded when mature worms were examined at concentrations of 100, 50 and 25 µM. The reduction in the number of ova was recorded with concentrations as low as 5 µM.

Effect of NPD1014 on mature worms

Schistosoma mansoni **worm (6 weeks old) killing and ovipositing under different concentrations of NPD-01014 compared to positive and negative controls.**

Test cpds	Dose	End of observation (5 days)					Notes
		% Worm killing			Ovipositing		
		Total worms	Male ♂	Female ♀	No. of ova/couple	% reduction	
Untreated control	(a)<br>(b)	0/16 (0%)<br>0/15 (0%)	0/8 (0%)<br>0/8 (0%)	0/8 (0%)<br>0/7 (0%)	100<br>110	-<br>-	Intact couples
Positive control (PZQ)	100 µM	13/13 (100%)	6/6 (100%)	7/7 (100%)	0	100%	-
	50 µM	15/15 (100%)	8/8 (100%)	7/7 (100%)	0	100%	-
	25 µM	15/15 (100%)	9/9 (100%)	6/6 (100%)	0	100%	-
	10 µM	14/14 (100%)	8/8 (100%)	6/6 (100%)	0	100%	-
	5 µM	14/14 (100%)	7/7 (100%)	7/7 (100%)	0	100%	-
Negative control (DMSO)	DMSO=2%(a)<br>DMSO=2%(b)	0/15 (0%)<br>0/15 (0%)	0/8 (0%)<br>0/8 (0%)	0/7 (0%)<br>0/7 (0%)	100<br>105	0%<br>5%	Intact couples
	DMSO=1%(a)<br>DMSO=1%(b)	0/14 (0%)<br>0/14 (0%)	0/7 (0%)<br>0/7 (0%)	0/7 (0%)<br>0/7 (0%)	110<br>120	0%<br>0%	Intact couples
	DMSO=0.5%(a)<br>DMSO=0.5%(b)	0/14 (0%)<br>0/14 (0%)	0/7 (0%)<br>0/7 (0%)	0/7 (0%)<br>0/7 (0%)	90<br>110	10%<br>0%	Intact couples
	DMSO=0.25%(a)<br>DMSO=0.25%(b)	0/15 (0%)<br>0/15 (0%)	0/7 (0%)<br>0/7 (0%)	0/8 (0%)<br>0/8 (0%)	105<br>100	0%<br>9%	Intact couples
	DMSO=0.125%(a)<br>DMSO=0.125%(b)	0/14 (0%)<br>0/14 (0%)	0/7 (0%)<br>0/7 (0%)	0/7 (0%)<br>0/7 (0%)	95<br>120	5%<br>0%	Intact couples
NPD-01014	100 µM (a)<br>100 µM (b)	7/13 (54%)<br>7/12 (58%)	7/7 (100%)<br>7/7 (100%)	0/6 (0%)<br>0/5 (0%)	0<br>0	100%<br>100%	Uncoupling
	50 µM (a)<br>50 µM (b)	6/14 (43%)<br>4/13 (31%)	6/9 (67%)<br>4/8 (50%)	0/5 (0%)<br>0/5 (0%)	0<br>0	100%<br>100%	Uncoupling
	25 µM (a)<br>25 µM (b)	0/16 (0%)<br>0/14 (0%)	0/8 (0%)<br>0/8 (0%)	0/8 (0%)<br>0/6 (0%)	120<br>-	0%<br>-	Intact couples, exaggerated spastic contractions<br>No couples to be tested
	10 µM (a)<br>10 µM (b)	0/16 (0%)<br>0/13 (0%)	0/9 (0%)<br>0/7 (0%)	0/7 (0%)<br>0/6 (0%)	130<br>-	0%<br>-	Intact couples, exaggerated spastic contractions<br>No couples to be tested
	5 µM (a)<br>5 µM (b)	0/18 (0%)<br>0/12 (0%)	0/9 (0%)<br>0/6 (0%)	0/9 (0%)<br>0/6 (0%)	125<br>-	0%<br>-	Intact couples, exaggerated spastic contractions<br>No couples to be tested

NB: Stock solution 5mM of test cpds dissolved in 1ml DMSO and then diluted to different concentrations with media.

NPD-01014 at concentrations of 100 and 50 µM, revealed worm killing of 54% and 58%, and of 43% and 31%, respectively, in two experiments (a & b), with uncoupling and absence of ova. No reduction in the number of ova was recorded when lower concentrations were tested. The compound showed exaggerated spastic contractions with the low concentrations of 25, 10 and 5 µM.

Effect of NPD1010 on early-mature worms

Schistosoma mansoni **early mature (4 weeks) worm killing and ovipositing under different concentrations of NPD-01014 compared to positive and negative controls.**

Test cpds	Dose	End of observation (5 days)					Notes
		% Worm killing			Ovipositing		
		Total worms	Male ♂	Female ♀	No. of ova/couple	% reduction	
Untreated control		0/14 (0%)	0/7 (0%)	0/7 (0%)	90	-	Intact couples
Positive control (PZQ)	100 µM	14/14 (100%)	7/7 (100%)	7/7 (100%)	0	100%	-
	50 µM	15/15 (100%)	8/8 (100%)	7/7 (100%)	0	100%	-
	25 µM	14/14 (100%)	7/7 (100%)	7/7 (100%)	0	100%	-
	10 µM	13/13 (100%)	7/6 (100%)	6/6 (100%)	0	100%	-
	5 µM	14/14 (100%)	7/7 (100%)	7/7 (100%)	0	100%	-
Negative control (DMSO)	DMSO=2%	0/15 (0%)	0/8 (0%)	0/7 (0%)	80	11%	Intact couples
	DMSO=1%	0/14 (0%)	0/8 (0%)	0/6 (0%)	85	6%	Intact couples
	DMSO=0.5%	0/15 (0%)	0/8 (0%)	0/7 (0%)	90	0%	Intact couples
	DMSO=0.25%	0/14 (0%)	0/7 (0%)	0/7 (0%)	95	0%	Intact couples,
	DMSO=0.125%	0/13 (0%)	0/7 (0%)	0/6 (0%)	95	0%	Intact couples
NPD-01014 Batch IV	100 µM	11/15 (73%)	6/7 (86%)	5/8 (63%)	0	100%	Uncoupling, exaggerated spastic contractions
	50 µM	0/14 (0%)	0/8 (0%)	0/6 (0%)	0	100%	Uncoupling, exaggerated spastic contractions
	25 µM	0/14 (0%)	0/7 (0%)	0/7 (0%)	0	100%	Intact couples, exaggerated spastic contractions
	10 µM	0/15 (0%)	0/8 (0%)	0/7 (0%)	0	100%	Intact couples, exaggerated spastic contractions
	5 µM	0/16 (0%)	0/8 (0%)	0/8 (0%)	65	28%	Intact couples, exaggerated spastic contractions

NB: Stock solution 5mM of test cpds dissolved in 1ml DMSO and then diluted to different concentrations with media.
