# Supplementary material for: Cloning and functional complementation of ten Schistosoma mansoni phosphodiesterases expressed in the mammalian host stages
Source: PLoS Negl Trop Dis. 2020 Jul 30;14(7):e0008447. doi: 10.1371/journal.pntd.0008447 (PMC7430754; doi:10.1371/journal.pntd.0008447)
Supplement: S3 Table — (PDF) [file pntd.0008447.s008.pdf]

S3 Table. Ability of SmPDEs to complement in the two yeast systems.

PDE	S. cerevisiae expressed?	Complementing S. cerevisiae?	S. pombe expressed	Complementing S. pombe?
SmPDE1	Yes	Yes	ND	
SmPDE4A	Yes	Yes	Yes	cAMP, ++++, cGMP +++
SmPDE4B	Yes	No – inactive	ND	
SmPDE7var	No	-	ND	
SmPDE8	Yes	Yes - low activity	Yes	cAMP ++
SmPDE9A	Yes	Yes – low activity	ND	
SmPDE9C	No	-	ND	
SmPDE11	Yes	Yes – low activity	Yes	cAMP +, cGMP ++

ND, not done.
