# Supplementary material for: Cloning and functional complementation of ten Schistosoma mansoni phosphodiesterases expressed in the mammalian host stages
Source: PLoS Negl Trop Dis. 2020 Jul 30;14(7):e0008447. doi: 10.1371/journal.pntd.0008447 (PMC7430754; doi:10.1371/journal.pntd.0008447)
Supplement: S4 Table — (PDF) [file pntd.0008447.s009.pdf]

**S4 Table.**
Primers used for the work on *T. brucei* strains and constructs.

| Gene/Use | Primer | Primer sequence | Use |
|---|---|---|---|
| Sm1 | HDK734 | ACGTCTAGACCACTGATCTG | F – 5' UTR-exon 1 |
| | HDK755 | CACTCCATAATCTAATAGATC | R – 5' UTR-exon 1 |
| | HDK670 | AGGTGATCGTGAACGTGAATTA | F – 3' UTR-exon 11 |
| | HDK735 | CTAGATCCCACTGCTAGATAC | R – 3' UTR-exon 11 |
| | HDK797 | ATGGGCTCTTGTGCATCAACT | F – full CDS |
| | HDK798 | TTAATATAAGCAAATTATTGAA | R – full CDS |
| | HDK875 | GCTCTTGTGCATCAACTGGT | F – qPCR |
| | HDK876 | CCATTGCATCACCTTCTTCA | R – qPCR |
| | | | |
| Sm2 | HDK1085 | AGAGCTGACCTACGAAATGA | F – 5' UTR-exon 1 |
| | HDK1086 | TTACTTCGTTTTCCCATGAT | R – 5' UTR-exon 1 |
| | HDK1089 | GCGTGAACAGCCTCCATCAT | F – 3' UTR-exon 24 |
| | HDK1090 | GTCCCATAGTACCAACATTTATG | R – 3' UTR-exon 24 |
| | HDK1022 | ATGAATAAAAACTATTCGAGAATATC | F – full CDS/ first half CDS |
| | HDK1023 | TTATGTACACTGATACTTAATAATATAAC | R – full CDS/ second half CDS |
| | HDK963 | CTGATTATTGGCCAACGGTA | R – first half CDS |
| | HDK962 | GGCTCAATTCATTCTGATGG | F – second half CDS |
| | HDK1218 | ACGATGTCAGTGTTCTTCTTGG | F - exon 2 |
| | HDK1220 | TGTGCTGGTTGTTGTTCGTC | R - exon 4 |
| | HDK1235 | GAACACTGGCTCAATTCATTC | F - exon 20 |
| | HDK1237 | CTTCGGAACTATATAAAGCAGC | R - exon 21 |
| | HDK1239 | CGTGACATAATTGTAGCAAC | F - exon 22 (pair HDK1023) |
| | | | |
| Sm4A | HDK730 | GACATGAATCGGTGTTGTAT | F – 5' UTR-exon 1 |
| | HDK752 | GTGACACTGCATCTGACAGTCGT | R – 5' UTR-exon 1 |
| | HDK753 | AGCTTCATTGATTTTGTGTGTCA | F – 3' UTR-exon 14 |
| | HDK731 | GTGGATATTAGTGTGATGTAGA | R – 3' UTR-exon 14 |
| | HDK782 | ATGGAGTTACGAACCGA | F – full CDS |
| | HDK783 | TTATGTGTTTCCTGAAGTTGTAGA | R – full CDS |
| | HDK871 | TAGTACAGGCGGAGCAACTG | F – qPCR |
| | HDK872 | AATGCATTCCGTATTCGTTG | R – qPCR |
| | | | |
| Sm4B | HDK764 | CATGGATGTCATTTCAACTTGA | F – 5' UTR-exon 1 |
| | HDK765 | GCGAATGGTGTTACAATAGGT | R – 5' UTR-exon 1 |
| | HDK766 | CTGCTGCATATCCTGTACACGCA | F – 3' UTR-intron pre-exon 11 |
| | HDK767 | GCATACACGTTTGGGCAGTGAT | R – 3' UTR-intron pre-exon 11 |
| | HDK812 | ATGATAATGTGGATCATTTGT | F – full CDS |
| | HDK813 | TCATTCATCATTTTCTGCATT | R – full CDS |
| | HDK873 | AAGAATTCCGAATCAACTGGT | F – qPCR |

| | | | |
|---|---|---|---|
| | HDK874 | ATCACCGGTCACTCCTATCG | R – qPCR |
| | | | |
| Sm4C | HDK732 | GTCTGGTTCAACGATTATATGA | F – 5’ UTR-exon 2 |
| | HDK669 | GAAATCTCGTGCCCGATTTAGA | R – 5’ UTR-exon 2 |
| | HDK754 | ATTGGTTTCATTGACTATATAG | F – 3’ UTR-exon 11 |
| | HDK733 | GACACAAAGGTGCTCTGTTCA | R – 3’ UTR-exon 11 |
| | HDK784 | ATGCGGTCAGCGTTCGAATCCT | F – full CDS |
| | HDK785 | TTACTGATCAATACTATTGTTC | R – full CDS |
| | HDK959 | CACCAATCAAATTCATTCAACA | R – first half CDS |
| | HDK958 | ATTCTAAATCGGGCACGAGA | F – second half CDS |
| | HDK845 | CCATTAGAACTTGTAAACCTGGA | R – second half CDS |
| | HDK1246 | CACATATTAGGATCATGCGGAG | R - exon 3 (pair HDK732) |
| | HDK1248 | TCCAATCATCATCCTTTAAGTAC | F - exon 5 |
| | HDK1250 | GAATTGAATTATGAAATGGTGC | R - exon 6 |
| | HDK1252 | TCAGTGATCTTGAAACATTTGC | F - exon 7 |
| | HDK1254 | TTTGTAAAATCACATTCTATCTGTG | R - exon 8 |
| | HDK1255 | GCATTTCAAGGCAATGTAATC | F - exon 9 |
| | HDK1256 | CAGTTTCTCTATCACACATTGGAC | R - exon 10 |
| | HDK1257 | CTGATTTAAGTAATCCAACAAAGC | F - exon 10 |
| | HDK1260 | GCTTTCACATACCAGTTTCTATTG | R - exon 11 |
| | | | |
| Sm7var | HDK1431 | ACTTGTCAGTCATGATTTAGTCC | F – 5’ UTR-exon 1 |
| | HDK1432 | TCGACCTCCATATGACTCCA | R – 5’ UTR-exon 1 |
| | HDK1433 | GGATCCATTCATTTAACTCAT | F – 3’ UTR-exon 12 |
| | HDK1434 | AGATGAAGTACATGATTGACTG | R – 3’ UTR-exon 12 |
| | HDK1455 | GCACACAATGAGGCGATCGAGAACCGATCA | F – exon 1 – exon 12 |
| | HDK1458 | GTAGTAGAATCCAATGATTCAGA | R – exon 1 – exon 12 |
| | HDK1474 | ACGTCGACATTCATTACCGG | F – qPCR |
| | HDK1475 | TGGTGATAGTGATGATGATGGTG | R – qPCR |
| | | | |
| Sm8 | HDK1112 | TGCATTTCAGATTTCACCAA | F – 5’ UTR-intron post-exon 1 |
| | HDK1080 | GTGTCCCACATTAGGACGAT | R – 5’ UTR-intron post-exon 1 |
| | HDK1083 | CCTATACCTGAACTAATGAAT | F – 3’ UTR-exon 12 |
| | HDK1084 | GGTATACAATCGATATGATTC | R – 3’ UTR-exon 12 |
| | HDK1024 | ATGAGCAAGGAAA | F – first half CDS |
| | HDK1082 | CATCACCTGCATGTGTAGCAT | R – first half CDS |
| | HDK1081 | CTAGCTAGAGAACCAGAGAACT | F – second half CDS |
| | HDK1026 | TTATTCTTGATGAATGGTTGTTGG | R – second half CDS |
| | HDK964 | TGTTCCATGGGAATGACAAT | F – qPCR |
| | HDK965 | GGATTATTAACATCGGAGCATTT | R – qPCR |
| | | | |
| Sm9A | HDK736 | CCTTCATTGAAGATCTTCTTC | F – 5’ UTR-exon 3 |
| | HDK756 | CTTATATGTATTAGCTAACATTG | R – 5’ UTR-exon 3 |

| | | | |
|---|---|---|---|
| | HDK757 | TCTGATCGAGAGAAATTAGAAG | F – 3’ UTR-exon 13 |
| | HDK737 | CTGATAGCCACCTGCACTAGT | R – 3’ UTR-exon 13 |
| | HDK846 | GTGAATGGATTACATATACCAT | F – exon3-exon14 |
| | HDK847 | GTGTCACCATAAGTTCATCAA | R – exon3-exon14 |
| | HDK940 | CACGTGAAGAATTAGGCATCC | F – qPCR |
| | HDK941 | GCCAGTTCAGTTCTTGCATTT | R – qPCR |
| | | | |
| Sm9B | HDK738 | CGGTAGCGGTTGCAGCAACA | F – 5’ UTR-exon 1 |
| | HDK758 | GTTGAGTCATAGATTGGAAGT | R – 5’ UTR-exon 1 |
| | HDK759 | ACTTGTTGGTCTTCCAGTTGCT | F – 3’ UTR-exon 7 |
| | HDK739 | CAGTGATGGCGTCACGGATGT | R – 3’ UTR-exon 7 |
| | HDK786 | ATGTCTGTATCCAATTGTTCA | F – full CDS |
| | HDK788 | GCTAATGATTCAAATAGAGGCA | R – full CDS |
| | HDK877 | GAACCGTGTTAGCGATGAGA | F – qPCR |
| | HDK878 | TGTGAGAACGGATCGACTTC | R – qPCR |
| | | | |
| Sm9C | HDK761 | CTAGTGACTCACATGTCCATA | F – 5’ UTR-exon 2 |
| | HDK762 | CGAATAGCTAGAAATTGATTC | R – 5’ UTR-exon 2 |
| | HDK763 | TGGAATCTGCGCATAGACGA | F – 3’ UTR-exon 8 |
| | HDK741 | GCCATATGCAATTCGAGTCA | R – 3’ UTR-exon 8 |
| | HDK789 | ATGATGTTTAAACGATTAATTC | F – first half CDS |
| | HDK961 | TGAATTGCCATATCTGTGCTT | R – first half CDS |
| | HDK960 | GCGTTCGATCTTATCACCAA | F – second half CDS |
| | HDK790 | CTAATTCATATTGTTTAATCTGA | R – second half CDS |
| | HDK960 | GCGTTCGATCTTATCACCAA | F – qPCR |
| | HDK961 | TGAATTGCCATATCTGTGCTT | R – qPCR |
| | | | |
| Sm11 | HDK1076 | GTGCATACCTTCATCGTCGATG | F – 5’ UTR-exon 1 |
| | HDK1067 | GACGCGTAACTTGTTGACTGA | R – 5’ UTR-exon 1 / R first half CDS |
| | HDK1077 | GGCTGTAAACGTAATCGTACATG | F – 3’ UTR-exon 8 |
| | HDK1078 | CATCAACACACGTACACAAGC | R – 3’ UTR-exon 8 |
| | HDK1090 | ATGTCCAGTTTAGTGAGAATGTG | F – first half CDS |
| | HDK1066 | GGAGAAGTCGTTCCGTTCTC | F – second half CDS |
| | HDK1028 | TTATTTTGATTCAACAGATGATTGAC | R – second half CDS |
| | HDK966 | TCCTTTCCCTTCCAATGAAC | F – qPCR |
| | HDK967 | TCACGTCAAGTTCACGACAA | R – qPCR |
| | | | |
| COX1 | HDK714 | TACGGTTGGTGGTGTCACAG | F – qPCR control |
| | HDK715 | ACGGCCATCACCATACTAGC | R – qPCR control |
| | | | |
| TbrB1 | HDK656 | TTAATTAAATGTTCATGAACAAGCCCTTTGGCAGCAAGC | F – full CDS TbrB1 |
| | HDK657 | TCTAGAACGAGTACTGCTGTTGTTGCCAGAACTCGT | R – full CDS TbrB1 |
| | HDK903 | AAGCTTATGTTCATGAACAAGCCCTTTG | F – First 210 bp TbrB1 |

| | | | |
|---|---|---|---|
| | HDK904 | TTAATTAATCCGCTTTGATCGAGAACCTC | R – First 210 bp TbrB1 |
| TbrB2 | HDK658 | TTAATTAAATGACACACAACGGTGGTCGTCATCTGCTTG | F – full CDS TbrB2 |
| | HDK659 | TCTAGAAGACGAAGCCCCAGTACTCCT | R – full CDS TbrB2 |
| | | | |
| Knock out vector TbrB1-B2 | HDK536 | CAGCTGCGAACTGACGGAAGTTGGT | F – 5' UTR TbrB1 |
| | HDK537 | AAGCTTCGTAGTGTCCAACTGTGCAC | R – 5' UTR TbrB1 |
| | HDK538 | GGATCCCGTGAGCCTGGTTGACGAAG | F – 5' UTR TbrB2 |
| | HDK539 | CCTGCAGGCAGAATTATGAGATGAAG | R – 5' UTR TbrB2 |
| | | | |
| Checking integration of constructs | HDK707 | GCTGGTGTTCGGGCTGTTAG | F - Upstream TbrB1-B2 locus |
| | HDK708 | GAGAGAATGCCAGTTGCCGATC | R - Downstream TbrB1-B2 locus |
| | HDK87 | GGCATACATCCACAACATGGT | R – mid-TbrB1 |
| | HDK775 | CTACGATGCTTGAGTCATCA | F – mid-TbrB2 |
| | HDK682 | CTTAAGATGGCCAAGCCTTTGTCTCA | F – mid-Blasticidin gene |
| | MB419 | TGAGGAAGAGTTCTTGCAGC | R – mid-Puromycin gene |
| | HDK1012 | CCGTTTACGTCGCCGTCCAG | R – check presence of SmPDE (pair HDK903) |
| | HDK713 | ATGCAAGCTAGGCCACACCT | F – check for circular plasmid |
| | HDK535 | CGGACAGGTATCCGGTAAGC | R – check for circular plasmid |
