# Supplementary material for: Cloning and functional complementation of ten Schistosoma mansoni phosphodiesterases expressed in the mammalian host stages
Source: PLoS Negl Trop Dis. 2020 Jul 30;14(7):e0008447. doi: 10.1371/journal.pntd.0008447 (PMC7430754; doi:10.1371/journal.pntd.0008447)
Supplement: S6 Table — (PDF) [file pntd.0008447.s011.pdf]

S6 Table. *S. pombe* strain list.

Strain name	Genotype
SP578	h^{90} *ade6-M216 leu1-32 cgs2-2*
CHP1207 h^{-}	*fbp1::ura4*$^{+}$ *ura4::fbp1-lacZ leu1-32 his7-366 pap1Δ::ura4*$^{-}$ *cgs2-2 git2Δ::his7*$^{+}$
CHP1265 h^{-}	*fbp1::ura4*$^{+}$ *ura4::fbp1-lacZ leu1-32 his7-366 pap1Δ::ura4*$^{-}$ *cgs2-2 git2Δ::his7*$^{+}$
CHP2344 h^{-}	*fbp1::ura4*$^{+}$ *ura4::fbp1-lacZ leu1-32 pap1Δ::ura4*$^{-}$ *cgs2-2 ars1::[pNMT1-SmPDE8] git2Δ::his7*$^{+}$
CHP2345 h^{-}	*fbp1::ura4*$^{+}$ *ura4::fbp1-lacZ leu1-32 pap1Δ::ura4*$^{-}$ *cgs2-2 git2Δ::his7*$^{+}$ *ars1::[pNMT1-SmPDE4A] git2Δ::his7*$^{+}$
CHP2346 h^{-}	*fbp1::ura4*$^{+}$ *ura4::fbp1-lacZ leu1-32 pap1Δ::ura4*$^{-}$ *cgs2-2 git2Δ::his7*$^{+}$ _*ars1::[pNMT1-SmPDE11] git2Δ::his7*$^{+}$
