# Supplementary material for: Cloning and functional complementation of ten Schistosoma mansoni phosphodiesterases expressed in the mammalian host stages
Source: PLoS Negl Trop Dis. 2020 Jul 30;14(7):e0008447. doi: 10.1371/journal.pntd.0008447 (PMC7430754; doi:10.1371/journal.pntd.0008447)
Supplement: S7 Table — (PDF) [file pntd.0008447.s012.pdf]

S7 Table. Primers used in the subcloning of SmPDEs for expression in *S. pombe*.

Name	Sequence
SmPDE11-F	AATTGAATAAGTTGAATTAATTATTTCAATCTCATTCTCACTTTCTGACTTATGAGTTCACTGGTGCGAATGT
SmPDE11-R	CAAACAAGGCATCGACTTTTTCAATAACCAACCAAAAAAATTTTACAGAACTTATCTAGACTTACTTTCCACAC
SmPDE8-F	AATTGAATAAGTTGAATTAATTATTTCAATCTCATTCTCACTTTCTGACTTATGAAGAGCGAGACACGTCAAC
SmPDE8-R	CAAACAAGGCATCGACTTTTTCAATAACCAACCAAAAAAATTTTACAGAAGCTTATCTAGATTCCTGATGTATGG
SmPDE4A-F	AATTGAATAAGTTGAATTAATTATTTCAATCTCATTCTCACTTTCTGACTTCATGGAACTCAGAACAGACAAAG
SmPDE4A-R	CAAACAAGGCATCGACTTTTTCAATAACCAACCAAAAAAATTTTACAGAATTACGTGTTCCCTGAGGTTGTA
nmt-F	GTCATTCGGCAATGTGCAGCG
nmt-R	TGCCTTCTGACATAAAACGCCTAG
SmPDE11-746F	CCAGGACGGGAGATTACGCT
SmPDE11-2452R	ACCTGTTAAGGTTGCACAGG
SmPDE4A-691F	ATGCTGGAGCGCCGACGTTC
SmPDE8-821F	ATGAGGTCGCCACTTTGCTG
