# Supplementary material for: Cloning and functional complementation of ten Schistosoma mansoni phosphodiesterases expressed in the mammalian host stages
Source: PLoS Negl Trop Dis. 2020 Jul 30;14(7):e0008447. doi: 10.1371/journal.pntd.0008447 (PMC7430754; doi:10.1371/journal.pntd.0008447)
Supplement: S1 File — (PDF) [file pntd.0008447.s013.pdf]

S1 File. Protein coding exons

SmPDE1-sv1

>**Smp_134500.1.e3** = SM_V7_7:?-12693768 (ATG maps to 12693993-12693991)
ATGGGCTCTTGTGCATCAACTGGTGTTTCACAAGCTGGTAGTCCGGCAAAAAGTCAATTAACGAATGGTATAC
AACAAACTTCATTTAATATACAAGAAATGAATCAAACAGAAATAAGAAATATAAATGAAGAAGGTGATGCAAT
GGCTGGTCAAGAAAAGAAAACTGATAATCCTAGAAGTAATTATGTATTGTCTGGTATAGATCTATTAGATTAT
GGAGTGG

>**Smp_134500.1.e4** = SM_V7_7:12691651-12691444
GGTTAAAAGATATACCACTTCGAACTTCTGATACAAATATGACAACAGATGAAGAACTTGATATGGAATGTGC
ATTATCTGGTGTATCACAATTTTTTTATAAAAATTTGACTACATGTACATTAGGTCAATATGCACAAATACCA
AATGATACAGATATGTTAAGATCAGTAAATACACCTGAATCATTAAGAGCTTGTTATGTAAG

>**Smp_134500.1.e5** = SM_V7_7:12689696-12689583
AATGCGTCATATCTATCGAATGATTGAAACCGATCGTATGGGTAAAGCAACATTAATGAAAAATATACAGTAT
GCAATAAACGTTATGGAAAATGCTTATATAGCAGAGAAACG

>**Smp_134500.1.e6** = SM_V7_7:12687772-12687593
ACGTATTCGTGAAGAAGAAGAAGATTTATCAGAAGCTGCTACAGAATATGTACCAGATGAAGTACGTAATTGG
CTTGCATCAACATTTACACGTACTGTACAAAGTGTTGGAATTGGTGATCAAAAGCCTAGATTTCGTAGTGTTG
CTAATGCAATTAGAGCTGGAATATTTGTTGAGAG

>**Smp_134500.1.e7** = SM_V7_7:12686521-12686455
AATTTATCGTCGTATGTCAAGTTGTTCCAATCTTATTGTTCCACCAAATGTACTTCTATTTCTTAAA

>**Smp_134500.1.e8** = SM_V7_7:12685028-12684909
ACTGGTTTAGATACATGGAATTTCGATGTATTTGGATTGAATGAAGCAAGTGAAAATCATGCATTGAAATTTG
TTGCATTCGAATTATTACATAAATATAATCTTATTAATAAATTCCAG

>**Smp_134500.1.e9** = SM_V7_7:12682744-12682604
ATAAATAGTACTGCATTAGAAAGTTTATTAATTCAACTTGAAACAGGTTATAGTAAATATAGTAATCCATATC
ATAATTTGGTTCATGCAGCTGATGTTATGCAAACATGTCATATGATCATTTTTATGAATGATCTTAGG

>**Smp_134500.2.e10** = SM_V7_7:12678573-12678473
AATTGGTTAAATGATTTGGATATATTTGCTGTGTTATTCGCTGCAGTAATACATGATTATGAACATACTGGTA
CAACTAATAATTTCCATATAGCAACACG

>**Smp_134500.1.e11** = SM_V7_7:12678088-12677963
TTCTGAATTAGCTTTAATTTATAATGATCGTGGAGTTTTGGAAAATCATCATGTTAGTGCAGTTTTCCGTTTA
ATGCAAGAAGAAGAATTTTCAATATTAACTGGATTGGAAGCTGATCAATACAA

>**Smp_134500.1.e12** = SM_V7_7:12676710-12676609
AGAATTCCGTCAACTTGTAATTGATATGGTTTTATGCACGGATATGTCATTGCACTTCCAACAAATTAAAAAT
ATGAAAACTATGATTTCAATGCCAGAAAG

>**Smp_134500.1.e13** = SM_V7_7:12674956-12674836
TATTGATAAAACAAAAGCACTATCTCTCATTGTTCATTGTGCTGATATTTCACATCCAGCGAAAGAATGGGCT
CTACACGAACAATGGTCTGATATATTGTGTGAAGAATTCTTTAGACAA

>**Smp_134500.1.e14** = SM_V7_7:12672018-12671937
GGTGATCGTGAACGTGAATTAAACCTTCCAATATCACCATTATGTGATCGTAATACGGTTGTTGTACCACAAT
CACAAATTG

>**Smp_134500.1.e15** = SM_V7_7:12671010-12670870
GTTTCATTGATTTCATTGTTGAACCAAGTTTCCAAGTTCTTGGTGATATGATTGAACGTATTGTTAATCCAAC
ACAAACTGAAGGAGTTCTACCAACAGATACAACAAGTCCTAAGCCTAAATCATCTGATCAAGAAACTG

>**Smp_134500.1.e16** = SM_V7_7:12666030-? (stop codon maps to 12665944-12665942)
TCGGTGAACAAGTCGTTCCACGTCCATGGGTTGAGCATTTCAAAGAAAATAAGGAATCATGGTCAAAGAAACT
TCCACCGAAAACTTGA

SmPDE1-sv2

>**Smp_134500.1.e3** = SM_V7_7:?-12693768 (ATG maps to 12693993-12693991)
ATGGGCTCTTGTGCATCAACTGGTGTTTCACAAGCTGGTAGTCCGGCAAAAAGTCAATTAACGAATGGTATAC
AACAAACTTCATTTAATATACAAGAAATGAATCAAACAGAAATAAGAAATATAAATGAAGAAGGTGATGCAAT
GGCTGGTCAAGAAAAGAAAACTGATAATCCTAGAAGTAATTATGTATTGTCTGGTATAGATCTATTAGATTAT
GGAGTGG

>**Smp_134500.1.e4** = SM_V7_7:12691651-12691444
GGTTAAAAGATATACCACTTCGAACTTCTGATACAAATATGACAACAGATGAAGAACTTGATATGGAATGTGC
ATTATCTGGTGTATCACAATTTTTTTATAAAAATTTGACTACATGTACATTAGGTCAATATGCACAAATACCA
AATGATACAGATATGTTAAGATCAGTAAATACACCTGAATCATTAAGAGCTTGTTATGTAAG

>**Smp_134500.1.e5** = SM_V7_7:12689696-12689583
AATGCGTCATATCTATCGAATGATTGAAACCGATCGTATGGGTAAAGCAACATTAATGAAAAATATACAGTAT
GCAATAAACGTTATGGAAAATGCTTATATAGCAGAGAAACG

>**Smp_134500.1.e6** = SM_V7_7:12687772-12687593
ACGTATTCGTGAAGAAGAAGAAGATTTATCAGAAGCTGCTACAGAATATGTACCAGATGAAGTACGTAATTGG
CTTGCATCAACATTTACACGTACTGTACAAAGTGTTGGAATTGGTGATCAAAAGCCTAGATTTCGTAGTGTTG
CTAATGCAATTAGAGCTGGAATATTTGTTGAGAG

>**Smp_134500.1.e7** = SM_V7_7:12686521-12686455
AATTTATCGTCGTATGTCAAGTTGTTCCAATCTTATTGTTCCACCAAATGTACTTCTATTTCTTAAA

>**Smp_134500.1.e8** = SM_V7_7:12685028-12684909
ACTGGTTTAGATACATGGAATTTCGATGTATTTGGATTGAATGAAGCAAGTGAAAATCATGCATTGAAATTTG
TTGCATTCGAATTATTACATAAATATAATCTTATTAATAAATTCCAG

>**Smp_134500.1.e9** = SM_V7_7:12682744-12682604
ATAAATAGTACTGCATTAGAAAGTTTATTAATTCAACTTGAAACAGGTTATAGTAAATATAGTAATCCATATC
ATAATTTGGTTCATGCAGCTGATGTTATGCAAACATGTCATATGATCATTTTTATGAATGATCTTAGG

>**Smp_134500.1.e10** = SM_V7_7:12678576-12678473
CAGAATTGGTTAAATGATTTGGATATATTTGCTGTGTTATTCGCTGCAGTAATACATGATTATGAACATACTG
GTACAACTAATAATTTCCATATAGCAACACG

>**Smp_134500.1.e11** = SM_V7_7:12678088-12677963
TTCTGAATTAGCTTTAATTTATAATGATCGTGGAGTTTTGGAAAATCATCATGTTAGTGCAGTTTTCCGTTTA
ATGCAAGAAGAAGAATTTTCAATATTAACTGGATTGGAAGCTGATCAATACAA

>**Smp_134500.1.e12** = SM_V7_7:12676710-12676609
AGAATTCCGTCAACTTGTAATTGATATGGTTTTATGCACGGATATGTCATTGCACTTCCAACAAATTAAAAAT
ATGAAAACTATGATTTCAATGCCAGAAAG

>**Smp_134500.1.e13** = SM_V7_7:12674956-12674836
TATTGATAAAACAAAAGCACTATCTCTCATTGTTCATTGTGCTGATATTTCACATCCAGCGAAAGAATGGGCT
CTACACGAACAATGGTCTGATATATTGTGTGAAGAATTCTTTAGACAA

>**Smp_134500.1.e14** = SM_V7_7:12672018-12671937
GGTGATCGTGAACGTGAATTAAACCTTCCAATATCACCATTATGTGATCGTAATACGGTTGTTGTACCACAAT
CACAAATTG

>**Smp_134500.1.e15** = SM_V7_7:12671010-12670870
GTTTCATTGATTTCATTGTTGAACCAAGTTTCCAAGTTCTTGGTGATATGATTGAACGTATTGTTAATCCAAC
ACAAACTGAAGGAGTTCTACCAACAGATACAACAAGTCCTAAGCCTAAATCATCTGATCAAGAAACTG

>**Smp_134500.1.e16** = SM_V7_7:12666030-? (stop codon maps to 12665944-12665942)
TCGGTGAACAAGTCGTTCCACGTCCATGGGTTGAGCATTTCAAAGAAAATAAGGAATCATGGTCAAAGAAACT
TCCACCGAAAACTTGA

SmPDE4A

>**SmPDE4A.e1** = SM_V7_ZW:?-17008106 (ATG maps to 17008122-17008120)
ATGGAGTTACGAACCGA

>**Smp_134140.5.e2** = SM_V7_ZW:16980709-16980554
TAAAGTGATTTCATCAAACGATACGATATCTTCGAGTCAAGAACCTCAGTTATCTCCACAAATTCCTGCAGTT
GTTAAACCACGGCGTGGATCTATATTTGTTTCAGCACTTGCAGCGGAACAACATGGAGCATCATCTTTATCAA
CAGATAATAC

>**Smp_134140.5.e3** = SM_V7_ZW:16974758-16974546
AGGATCGATGATTGTTAGATTGGGTCGATCTAGTACAGGCGGAGCAACTGCTGCAACTAGTACCAGTTCATCA
AGTACAAATGGAATAACTGCAGTCTTACCAGATTCATCTGTTGAAGGTGTGATTGTGACACCATTTGCTCAAG
TGCTTGTTAGTATGCAACGAATACGGAATGCATTTATCAGGTTAACCGCTGCTCAAGTATCAAATAA

>**Smp_134140.1.e2** = SM_V7_ZW:16947734-16947679
ATATAACATCACAACCGTATTTGATTCTGTTCCGACTGGCAATCTAACACCAGATA

>**Smp_134140.1.e3** = SM_V7_ZW:16936671-16936559
GTAGTGATTATAAAATAATTGCAAATGAAACTCTTGAAGAATTGGAATGGTGTCTTAAACAATTGGAAAATAT
CCAGACGAAGAGGCCTGTTTCTGATATGGCTTTTTCAAAA

>**Smp_134140.1.e4** = SM_V7_ZW:16935632-16935539
TTTAAACGTTTACTCAACAAAGAGTTGAATAGTTTTGGTGAAGCTGATAAATCTAGACATCAAATATCTGCAT
ATATTTGTGAAACATTTTTAG

>**Smp_134140.1.e5** = SM_V7_ZW:16932595-16932195
AAACTGAAAAAGACGTTGAAACCAATGAAGAGATTGACTCTATGCTGGAACGACGACGTAGTAGTGGTCAGTC
GCACAATTCAACCAGTGGACAGGATACAAACACTACAAGTAAAAGGCAGGCTTCAGGAACTGGTGATCAAAAT
GCTAATCCAAATACTACAAGGACACCGGATACTTCTTCTTCAGTTTCCTCATCAGTTATAAAAACAAGAATAG
GATCAACTGGAAGTATGTCAACTGAATCTCGTAAAAGCGCTCAACTAAATGATTCTAGCGGTTTGCTAACAAC
AAAATTGCCATCCAGTTCAAAGTTAACGAGTCAAAATGTAGACGACGGTAACGGGCCATTCCTACCAATTCAT
GGGGTTGAAACGCCTAATGATAATGAGTTGGAGGAA

>**Smp_134140.1.e6** = SM_V7_ZW:16926864-16926763
AGATTCTCACTGTGTTTGGACGAATGGGGTGTAGATATCTTTGAAATTGATCGACTTTCTAATGGACATGCTC
TTACAACAGTTGCCTATAGAATATTTCAG

>**Smp_134140.1.e7** = SM_V7_ZW:16923100-16922936
AAACGTGATCTACTCAAAACATTTTGTATTGATCCTCATGTTTTTGTACGTTATCTGTTAAGAGTTGAATCAA
CTTATCATGCTGATGTTCCTTATCATAACTCAATGCATGCAGCCGATGTTCTTCAAACAGCACATTTTTTGCT
ACAAGCAGAAGCATTGGAC

>**Smp_134140.1.e8** = SM_V7_ZW:16920765-16920666
GATGTTTTCAGCGATTTGGAAATTTTAGCTGTTTTGTTTGCCGCAGCTATACATGACGTAGATCATCCTGGAG
TAACTAATCAATTTCTAATCAATACTG

>**Smp_134140.1.e9** = SM_V7_ZW:16914549-16914395
GTCATGAATTAGCCTTACAATATAATGATGCATCCGTTCTGGAAAACCATCATCTTTATATGGCATTTAAAAT
TTTAACTGAAAAAGACTGTGATATATTTGCTAATTTAGGTGGAAAAAAACGTCAAACATTACGTAGAATGGTT
ATAGAATTG

>**Smp_134140.1.e10** = SM_V7_ZW:16912910-16912788
GTGTTAGCAACAGATATGTCTAAGCATATGAGTTTATTAGCTGATTTAAGAACAATGGTTGAAACTAAAAAAG
TTTCTGGATCCGGTATGTTGAATTTGGACAATTATGCTGATCGTATACAG

>**Smp_134140.1.e11** = SM_V7_ZW:16910830-16910648
ATATTACAAAATATGATTCATTGTGCTGATCTTAGTAATCCAGCTAAGCCATTACGATTATATCGTAAATGGA
CTGGACGTTTAATTGAAGAATTTTTCAGACAGGGGGATAAAGAAAGAGAGTTGTCATTGGAAATTAGCCCAAT
GTGTGATCGTGAATCGGTAGAAGTTGAAAAGTCTCAG

>**Smp_134140.1.e12** = SM_V7_ZW:16907346-? (stop codon maps to 16907127-16907125)
GTTAGCTTCATTGATTTTGTGTGTCACCCATTATGGGAAACATGGTGTGATCTAGTACACCCCTGTGCTCAAT
TAATTCTAGATACATTGGAAGATAATCGTGACTGGTATGAATGTCATATAAAAGAATCCAAAATGAAAGTTAC
TCAGTTAGCTCGACCAAAATTAGCTACTGCCGCAGAAGATGATGAAGAAATTTCTACAACTTCAGGAAACACA
TAA

SmPDE4B-a1

>**Smp_141980.1.e1** = SM_V7_6:?-11943182 (ATG maps to 11942032)
ATGATAATGTGGATCATTTGTTGTCGTAAACAAGAAACAAAAAAGAATAAAACAAAAGATAATTTACAGCTAA
GTTCAAAATACTCTATTAGTTATAATGATCATAGAATTAATGATAATCAAGATAATTGTAAGCTTAAAAATGA
TCTCAATATTCATACTAAATTAAGTCATGATTCATATGCTAATATTGAAAATGGACATTATCCTAAACAAGAA
AGAAGAAGTTTACCTGATGTTGTTATACCTTCAACAAATTTACCAATTCCAAGATCTAGTTTACAAATTGATT
ATGAAACTGGACGTAGATCAACAATATACTTAGAAACTACAGTAAAATTAGCTGAATCCGAAAGTATTAATTT
AGATGATAATTATTCTTCTAATATATTTAATGAAACATTTCTTAATAATATAAAATTAAATATGGCAAATAGA
GATTTTCTATTAGATAAACCAAGAGCTCATAGTCTTATTTCATCAACATATCAAAAACAATTAACTAATAATC
ATAAAGGACGTCAAGGTAGAAAGTCCGATGTTTCACGTAGTGATAATCATGAAAAAAAATTAATTTCCAAGAA
AAGACTTAGTCTTTTTAAAGTGAAACCATTAAAACGTTTTCGTAAATCAAAAGATAGTTTACATTCACTGAGC
CCACAAAAATGTACTATTAGAAATGATAAATCAAACATGATATCAACTATGACATGTGATAGGTCAACTAATT
CGAAAACGAAATCCTCATTGAAACGGATTAAACCTTCGAAATTATTTTCAACCAATAAATCATCTCATGAAAA
TATTGGTATCACTAAAGATTTAAATAATCATGAAAGTTGTTCTGAACGATCAGAGGATAGTTTAGGACAATTA
CCTATGACAAGGAATTCTATGTTCAAACATATACAAACACGTACTACTAATTTATATCAAACAACAAAAAATT
TTGATTTAAATAAACAAAATTATTTAAATAATATTGAACATTCTATGACAAATTCTAAAAATACTGTATTAAG
ACGTGGTTCTCTTTGTATTTTTAGTCAAATTGATGAACCTATTGTAACACCATTCGCACAGATATTGGCTAGT
TTAAGAAAAGTTCGATTTAATTTCATCTTATTAACAAATGTAACAAGTACAAGAGA

>**Smp_141980.1.e2** = SM_V7_6:11966673-11966737
TTCACGATTTGGAGCTGTTCAACCGATACAATCTACTGAAGATGGAAATAGTTCTTCACACCTAG

>**Smp_141980.1.e3** = SM_V7_6:11969031-11969152
GTTGTAGTGGAGGACAAAATAAGATAACAGCCAGTGAAACATTGGAAGAACTTGAATGGTGTTTAGAAAGATT
AGAAAATATACAAACCCATCGATCTGTTAGCGATATGGCATCAAGTAAA

>**SmPDE4B-a1.e4** maps to SM_V7_6:11977649-11978392 (1 synonymous nt substitution compared to Smp_141980.1.e4: nt 543 of the exon, C instead of T)
TTTAAGAAAATGTTAAATAAAGAATTAAGTCAATTTGCTGATGCTGGACAAAGTGGCAAACAAATATCCGAAT
ATATATGTTCAACATTTTTAGATTCAAAAGAAAATGATCCATTAACAACTACAAGTCATTCATCTGTAATATC
ACATAATAATACACATGGAATAATGTCTAATAGTATAAATAAAGATATTATGATAAATAATCATGATAATCCT
TCTGAAATGAATACATGTACACCATCAGATAGTATAAAGAATTCCGAATCAACTGGTATGTATTTATTCAAAA
GTGTTGAACCCGGTGAAATAATTCAAACGACAACAACAACAACAACACTGACTGACAATATTGATGGTGATAC
AACGATAGGAGTGACCGGTGATAGTAGCAGTAGTAATATTCCTCATACAATAAATAGTACAACTGTAACAATT
GGATCTACTACAATGAATAAATCATTGTCAAAATTATCCTCTTCACTATCGACTTTATCATTGAAAATGAATA
GTAATGTGAATGTAAATGAAAATACTAATAACAATAATAATAATAATGACGATAATAATATTAATATAATGAT
GAAATCTAGTGGAAATTCTCACACCACTATGTCAATTCCTCCTTCTGGTACATCACCTCTATCTCATCAACAT
CATAGTGAATGTGATAAAATTCATTGTTCACAAACACAAATTGTACCATATATAATAAATTCAACAAATCCAA
AGAAACTTGAAGAT

>**Smp_141980.1.e5** = SM_V7_6:11980752-11980850
CTTCTAAAAACTTCCCTTGATCTATGGGGTATTGATATATTCGAAGTAGATCAGCTTACTACTAATCCATTAA
CGTGTATATTTTATAATATTGTACAG

>**Smp_141980.1.e6** = SM_V7_6:11983603-11983764
AAAAGAAATCTTTTGCAAAAATTTGCTATACCTGAACGTAATCTACTTCTATACATGACTGCTGTTGAAGAGA
AATATAACAATAACCCATATCATAATCGTGTTCATGCAGCTGATGTTGTACAGTCAACACATGTATTATTAAA
TGCACAATCATTAGAG

>**Smp_141980.1.e7** = SM_V7_6:11986870-11986969
TCTGTATTTACAGATTTAGAAATTTTCACAGTTCTATTTGCTTGTGCCATTCATGATGTCGGTCATCCAGGAG
TTACAAATCAGTATTTAATTAATACAA

>**Smp_141980.1.e8** = SM_V7_6:11991355-11991509
ATGATCAATTAGCAATTCTATACAATGATTCATCTGTATTGGAAAATCATCATTTAGCTATAGCATTTTCCCT
ATTAGGTCAACCAGGTCATGATGTTTTTGAGAACTTTCCACGTAAACAACGACTAAGTTCTAGACGTATGATT
ATTGATATG

>**Smp_141980.1.e9** = SM_V7_6:11994257-11994379
GTTTTAGCTACAGATATGTCCAAACATATGAGTTTGTTAGCTGATTTGAAAACAATGGTTGAAACGAAAAAGG
TTGCTGGATCCGGTATACTTACTTTAGAAAATTATATTGACAGAATGCAG

>**Smp_141980.1.e10** = SM_V7_6:11998788-11998970
ATCTTGCAAAATATGGTACATTGTGCTGATTTAAGTAATCCAGCTAAACCATTAGATTTGTATAGACAATGGA
CTAATCGTGTTATGGAAGAACTCTTTCAACAAGGTGATAAAGAACGTGAATTAGGCATTGAAATAAGTCCAAT
ATGTGATCGAAATACAGCAACTATTGAAAAATCACAG

>**Smp_141980.1.e11** = SM_V7_6:12001910-? (stop codon maps to 12002074)
GTCAGCTTTATTGATTACATTGTTCATCCATTATGGGAAACATGGTCAGATTTAGTTTATCCAGATGCACAAA
CTATTTTAGAAACACTAGAAGATAATCGTGAATGGTATTATAATCAAATTAATGAGAATAATAATGATAATAA
TGCAGAAAATGATGAATGA

SmPDE4B-a2

>**Smp_141980.1.e1** = SM_V7_6:?-11943182 (ATG maps to 11942032)
ATGATAATGTGGATCATTTGTTGTCGTAAACAAGAAACAAAAAAGAATAAAACAAAAGATAATTTACAGCTAA
GTTCAAAATACTCTATTAGTTATAATGATCATAGAATTAATGATAATCAAGATAATTGTAAGCTTAAAAATGA
TCTCAATATTCATACTAAATTAAGTCATGATTCATATGCTAATATTGAAAATGGACATTATCCTAAACAAGAA
AGAAGAAGTTTACCTGATGTTGTTATACCTTCAACAAATTTACCAATTCCAAGATCTAGTTTACAAATTGATT
ATGAAACTGGACGTAGATCAACAATATACTTAGAAACTACAGTAAAATTAGCTGAATCCGAAAGTATTAATTT
AGATGATAATTATTCTTCTAATATATTTAATGAAACATTTCTTAATAATATAAAATTAAATATGGCAAATAGA
GATTTTCTATTAGATAAACCAAGAGCTCATAGTCTTATTTCATCAACATATCAAAAACAATTAACTAATAATC
ATAAAGGACGTCAAGGTAGAAAGTCCGATGTTTCACGTAGTGATAATCATGAAAAAAAATTAATTTCCAAGAA
AAGACTTAGTCTTTTTAAAGTGAAACCATTAAAACGTTTTCGTAAATCAAAAGATAGTTTACATTCACTGAGC
CCACAAAAATGTACTATTAGAAATGATAAATCAAACATGATATCAACTATGACATGTGATAGGTCAACTAATT
CGAAAACGAAATCCTCATTGAAACGGATTAAACCTTCGAAATTATTTTCAACCAATAAATCATCTCATGAAAA
TATTGGTATCACTAAAGATTTAAATAATCATGAAAGTTGTTCTGAACGATCAGAGGATAGTTTAGGACAATTA
CCTATGACAAGGAATTCTATGTTCAAACATATACAAACACGTACTACTAATTTATATCAAACAACAAAAAATT
TTGATTTAAATAAACAAAATTATTTAAATAATATTGAACATTCTATGACAAATTCTAAAAATACTGTATTAAG
ACGTGGTTCTCTTTGTATTTTTAGTCAAATTGATGAACCTATTGTAACACCATTCGCACAGATATTGGCTAGT
TTAAGAAAAGTTCGATTTAATTTCATCTTATTAACAAATGTAACAAGTACAAGAGA

>**Smp_141980.1.e2** = SM_V7_6:11966673-11966737
TTCACGATTTGGAGCTGTTCAACCGATACAATCTACTGAAGATGGAAATAGTTCTTCACACCTAG

>**Smp_141980.1.e3** = SM_V7_6:11969031-11969152
GTTGTAGTGGAGGACAAAATAAGATAACAGCCAGTGAAACATTGGAAGAACTTGAATGGTGTTTAGAAAGATT
AGAAAATATACAAACCCATCGATCTGTTAGCGATATGGCATCAAGTAAA

>**SmPDE4B-a2.e4** = SM_V7_6:11977649-11978061 + CT + 11978070-11978392 (CT replaces AATAAATA)
TTTAAGAAAATGTTAAATAAAGAATTAAGTCAATTTGCTGATGCTGGACAAAGTGGCAAACAAATATCCGAAT
ATATATGTTCAACATTTTTAGATTCAAAAGAAAATGATCCATTAACAACTACAAGTCATTCATCTGTAATATC
ACATAATAATACACATGGAATAATGTCTAATAGTATAAATAAAGATATTATGATAAATAATCATGATAATCCT
TCTGAAATGAATACATGTACACCATCAGATAGTATAAAGAATTCCGAATCAACTGGTATGTATTTATTCAAAA
GTGTTGAACCCGGTGAAATAATTCAAACGACAACAACAACAACAACACTGACTGACAATATTGATGGTGATAC
AACGATAGGAGTGACCGGTGATAGTAGCAGTAGTAATATTCCTCATACctGTACAACTGTAACAATTGGATCT
ACTACAATGAATAAATCATTGTCAAAATTATCCTCTTCACTATCGACTTTATCATTGAAAATGAATAGTAATG
TGAATGTAAATGAAAATACTAATAATAATAATAATAATAATGACGATAATAATATTAATATAATGATGAAATC
TAGTGGAAATTCTCACACCACTATGTCAATTCCTCCTTCTGGTACATCACCTCTATCTCATCAACATCATAGT
GAATGTGATAAAATTCATTGTTCACAAACACAAATTGTACCATATATAATAAATTCAACAAATCCAAAGAAAC
TTGAAGAT

>**Smp_141980.1.e5** = SM_V7_6:11980752-11980850
CTTCTAAAAACTTCCCTTGATCTATGGGGTATTGATATATTCGAAGTAGATCAGCTTACTACTAATCCATTAA
CGTGTATATTTTATAATATTGTACAG

>**Smp_141980.1.e6** = SM_V7_6:11983603-11983764
AAAAGAAATCTTTTGCAAAAATTTGCTATACCTGAACGTAATCTACTTCTATACATGACTGCTGTTGAAGAGA
AATATAACAATAACCCATATCATAATCGTGTTCATGCAGCTGATGTTGTACAGTCAACACATGTATTATTAAA
TGCACAATCATTAGAG

>**Smp_141980.1.e7** = SM_V7_6:11986870-11986969
TCTGTATTTACAGATTTAGAAATTTTCACAGTTCTATTTGCTTGTGCCATTCATGATGTCGGTCATCCAGGAG
TTACAAATCAGTATTTAATTAATACAA

>**Smp_141980.1.e8** = SM_V7_6:11991355-11991509
ATGATCAATTAGCAATTCTATACAATGATTCATCTGTATTGGAAAATCATCATTTAGCTATAGCATTTTCCCT
ATTAGGTCAACCAGGTCATGATGTTTTTGAGAACTTTCCACGTAAACAACGACTAAGTTCTAGACGTATGATT
ATTGATATG

>**Smp_141980.1.e9** = SM_V7_6:11994257-11994379
GTTTTAGCTACAGATATGTCCAAACATATGAGTTTGTTAGCTGATTTGAAAACAATGGTTGAAACGAAAAAGG
TTGCTGGATCCGGTATACTTACTTTAGAAAATTATATTGACAGAATGCAG

>**Smp_141980.1.e10** = SM_V7_6:11998788-11998970
ATCTTGCAAAATATGGTACATTGTGCTGATTTAAGTAATCCAGCTAAACCATTAGATTTGTATAGACAATGGA
CTAATCGTGTTATGGAAGAACTCTTTCAACAAGGTGATAAAGAACGTGAATTAGGCATTGAAATAAGTCCAAT
ATGTGATCGAAATACAGCAACTATTGAAAAATCACAG

>**Smp_141980.1.e11** = SM_V7_6:12001910-? (stop codon maps to 12002074)
GTCAGCTTTATTGATTACATTGTTCATCCATTATGGGAAACATGGTCAGATTTAGTTTATCCAGATGCACAAA
CTATTTTAGAAACACTAGAAGATAATCGTGAATGGTATTATAATCAAATTAATGAGAATAATAATGATAATAA
TGCAGAAAATGATGAATGA

SmPDE4C

>**SmPDE4C.e1** = SM_V7_5:?-19411586 (ATG maps to 19411354), 1 non-synonymous nt substitution compared to Smp_334600.1.e1: nt 122 of the exon, T versus C => Met41 versus Thr41
ATGACAACTATATCTACAAATATTCATTCTAAATCAATTGGAAATAGTTTATATGAACGACGTAGAAATTGGCGTATAC
ATCAAATGAAATCATGTATGGAACCACCAATAAATGTTTCAATGACATCAATTTATAATAAAGATGATGATGATGGAA
TAAGAATGAATATTATGTCTAAAAAATCAGATATATTATGTAATAAAGAAAATTGGTGGGGAAAAACTATTAGTCA

>**Smp_334600.1.e2** = SM_V7_5:19471809-19471895
TGAACCCAGTGTATTAATAACTCCTTTTGCCCAAATTTTAGCCATTCTAAATCGGGCACGAGATTTCCTATCC
AATTATACTTCAAA

>**Smp_334600.1.e3** = SM_V7_5:19473402-19473475
CTCTCCCTCTTATTCTCCGCATGATCCTAATATGTGTAGCACGAAGTCAATCGGCTGTGATGAAAAATATGTT
A

>**Smp_334600.1.e4** = SM_V7_5:19477717-19477838
ATTGTCTACATCGATTTGATGTACATTATGTGAATCAATTGTTGAATGAATTTGATTGGTGTTTAGAAGTATT
GGACAGTTTACAAAGTAAACGTTCTGTAAGCAGTTTAACCCGAATGAAA

>**SmPDE4C.e5** maps to SM_V7_5:19483831-19484412 (1 non-synonymous nt substitution compared to Smp_334600.1.e5: nt 53 of the exon: G versus A => G190 versus D190)
TTACGTAGTCTTCTTAGTCAAGAATTGGCCGCTTCATTCAATCAATCAAATGGTAAAATTATCAAGAAAGATT
CATTGAAAAAATCAACCACAACAACTCACAATGTTCGTTTTGATGATTTAACTTTAAAAAACTCCTCTTTACT
AGTACATGATCAAGATAATAATAATAAAAGGTCAAATATTAGTCAAATACAAAAACGTAAACAACAATTTAGA
AGTTGTAGATCACAAGTTTGTGAATATATTTGTAAAACATTTCTTGAAGAAGATGATGATGACGAAGATGTTG
ACAATAATCATGAAGATGATGATGGTGATGCTGAGGAAGACAACTCCAAAAGCATATCATCTCATAATCCATC
TACAGAAAAATCTATTCATTTAAATAGTCCAAAGTCAAAACTTCGATTCAGTGATAAATATGAAGAAAGAATG
AATGAATCCAGCGAGACAACCGAGTCTACAACTGAATTAAAATCTACCTCATCTACACAATTCAATGATACAG
ATGTAATATCTGATACGTTAAAGTTAATTATATTGAATATTGAACATGTTGATCATTCAATAGTGGAAAAT

>**Smp_334600.1.e6** = SM_V7_5:19487546-19487647
TTTATCATAAATAATCAATCGAATTTAGCTCCAGATCTATTTAAATTAGATCAAATTTCCAATCATCATCCTT
TAAGTACATTTGGCTTTTATTTATTTATG

>**Smp_334600.1.e7** = SM_V7_5:19490259-19490423
AAAACGAATGTATTACAGAAATTGTCTATACCTTCAGTGACAATGTTGAATTGTTTAAGACAAATTGAGTCAC
GATACAATTCAACTGCACCATTTCATAATTCAATTCATGCCTTAGATGTACTACATGCAACTTATCAATTATT
TCAATGTAATAGTTTAAAA

>**Smp_334600.1.e8** = SM_V7_5:19493718-19493817
AATATCTTCAGTGATCTTGAAACATTTGCAATTTTCTTTGCTAGTGCTATACATGATATCGACCATCCTGGTT
TAACAAATCAATACCTTATAAATACAA

>**Smp_334600.1.e9** = SM_V7_5:19496470-19496627
ATCATGAATTAGCTCTATTATATAATGATATATCAGTTTTGGAAAATCATCATTTACATGTAGCATTTAAATT
AATTAATACACAGATAGAATGTGATTTTACAAAATATTTTACAAATCAACAAAAGCTGTTATTTCGTAAAATG
GTTATCGCTTTA

>**Smp_334600.1.e10** = SM_V7_5:19499653-19499775
GTATTATCTACCGATATGAGTAAACATATGTCATTATTGGCTGATTTAAAAACAAGCGTTGAAAAACAAAAGG
CATTTCAAGGCAATGTAATCAATCTGGATAGTTATTCAGCTCGTATGCAG

>**Smp_334600.1.e11** = SM_V7_5:19501903-19502085
ATATTAGAATGTATAATACATGCAGCTGATTTAAGTAATCCAACAAAGCCATTAAAAATCTATCAAGAATGGG
TATCAAGAATTATGGAAGAAATGTTTCGTCAAGGTGATCAAGAGAAACAATATGGAATTGAAATTAGTCCAAT
GTGTGATAGAGAAACTGCATGCATTTACAGTACACAA

>**SmPDE4C.e12** = SM_V7_5:19504240-19504380 + 19504384-? (Smp_334600.1.e.12 has an additional GAA codon after nt 141 of the exon => +E691)
ATTGGTTTCATTGACTATATAGTTTATCCATTATGGGAGACAATGGCTGAACTTCTACATCCAGGTTTACAAG
TTCTAATGGATAATATAACAAACAATAGAAACTGGTATGTGAAAGCAAAAGAGGAAGAAGAAGAAGAAGTAAA
AGAGGAGAATAAGAAGAACAATAGTATTGATCAGTAA

SmPDE7var-a1

>**Smp_153640.2.e2** = SM_V7_ZW:?-46450232 (ATG maps to 46450301-46450299)
ATGAGGCGATCGAGAACCGATCAAAAATCAGGATCATGGAGTCATATGGAGGTCGATTTAACAAATAAAA

>**Smp_153640.2.e3** = SM_V7_ZW:46431829-46431579
ATTTACAAAGAACCAAAAATTCATCGTGTAGACGAATTCGTTATCGATCAAATTCATTTAACGCATTTGTTTG
TCAACAAAGTGAACAAATGGCTGGGACAATACAATTATTTTATTTATATGAAGATAAAAATTCAAAGAATATG
TACAATCTTGATAAATCAAAAGAAAGAAAGAAATCACAAACAGTTGAAAGAATAACCGATGAAATCAAACTTA
GTTTTATAGATCGTCCAGTTGGACATGTAAAG

>**Smp_153640.2.e4** = SM_V7_ZW:46428504-46428447
ATTAAAAATTTAAGTAATTGGGATTTTAACATTTTTCATTTAAAACGAACAAGTTCTA

>**Smp_153640.2.e5** = SM_V7_ZW:46426116-46426021
ATTATACCATTCGTGATATTGGTTTACAAATAATGAATGAATATGATTTATTTCAAAAATTAAAATTAAATTA
TTTCATGATGGCTAGAATATTCA

>**Smp_153640.1.e5** = SM_V7_ZW:46419600-46419500
ACTCTATAGAAGCAGCTTACCACAATTTCAATCCGTATCATACAGCGTTGCATGCAGCCGATGTACTACAAGC
AGTGCATTGTTTTATATCACGAAGTCAA

>**Smp_153640.1.e6** = SM_V7_ZW:46405740-46405609
CTTCTGACCATTTTAAGTCCGACAGAAATTTTCGCTAGTCTATTGGCAGCTGCTTTACATGATGCTGATCATC
CCGGTGTGAATCAATCCTATCTAGAGAAGACAGGTGATTTTTTAGTTGATTTATATAAA

>**Smp_153640.1.e7** = SM_V7_ZW:46396634-46396543
TCTGTTTCTGTATTGGAAAAACATCATGCAAAATTTGGATTATGCATTCTTCAAGAAAATGGATTGTCTAATG
CATTAGAATTACATGAATG

>**Smp_153640.1.e8** = SM_V7_ZW:46394405-46394324
GGAATTCGTGCGTGACTGTTTTCTAAAATTAATTCCAGCTACTGACATTACATATCAAGGAGTATATCAAAAA
CAATTCAAA

>**Smp_153640.1.e9** = SM_V7_ZW:46390563-46390492
GATTTAACAAACTATCATATGGCTAATCCATCATTACCATTTACAATATCCGATCGGCTGTTGATCATGCAA

>**Smp_153640.1.e10** = SM_V7_ZW:46387570-46387390
ATGGCTTTAAAATGCTCAGATATAAGTAATCCCTGTCGAATTTGGCCAATATGTAAAGAATGGGCAATACGTG
TTTGTTGTGAATTATTTTGTCAGGGTGATAGAGAACGTTTTCAATGGTCATTACAACCTATACCAACAATGGA
TCGCACAAAATTTACTTTAGCGAGAATACAAAATG

>**Smp_153640.1.e11** = SM_V7_ZW:46380620-46380331
GATTTATTAGAGATATGGTGAAACCATTATTAACTGGATGGCATGAATTTCTTCAAAATAATCTTACATTGAA
AATTTTACAAAATTTAGATGAAAATTTAAAAAATTGGTTAACTGATTTATCTTTATCGTCGTCGTCGTCAACA
TCATCTTCATCAGGCATGACAAATTATGTTAATCGTCATAATTCATTAGATAGTCAACTGTTATCAACATTAC
CACTTAGTAATAATAATAATAATCAATCTATTTCATCAAATCAAATTAAACCAAAAACTATTAAATCAGAG

>**SmPDE7var-a1.e12** maps to SM_V7_ZW:46377004-? (stop codon:46376371-369); 2 non-syn substitutions compared to Smp_153640.1.e12: exon nt 545 (A>C => Q657> P657), nt 550 (G>T => V659> L659)
ATATCGAATCAAAAAAGTAAAATTGAATTACAAAAACCATTTATAGGATCCATTCATTTAACTCATTCACCAA
TTCAAGAAATTGAAACAATGAATGCATCTGAATCATTGGATTCTACTACTACTAATAATAATAATTTACATTC
AGGTAAATTACATCAAACAATATTTATTACAACACGTGTTATACGTCGACATTCATTACCGGAAACTCAATTA
GCTATTAGAAAAACATTTAATTTTTCATTATCAAATAAATCAAGTACTGTTGTTCTATTAAAAAATGATACAC
AAAATATATTATCATTACATTCAAGAAATAATAATAATTTACCAGTCGGTAAGATTAAATGTCAATCATCTAC
CTTCAAATCACCATCATCATCACTATCACCATCGTCTACATGTACAGTATCTGCAAATCTATTACAAATGTTA
TATGAAGAATTAAAACATAATAATAAATCAAATTATGTATTATCTAAAATTGATATAAAATCAATTCCAAATA
CAAATTGTTATAAAGATAATAAATTATTAGAACAAAAAGTATTAGATTTAAATTATGATCGTACATTATTACG
ATTTTCTGCACTTGCTCATCGTCGTAGTAGTGCACCAATTACAGAACATTAA

SmPDE7var-a2

>**Smp_153640.2.e2** = SM_V7_ZW:?-46450232 (ATG maps to 46450301-46450299)
ATGAGGCGATCGAGAACCGATCAAAAATCAGGATCATGGAGTCATATGGAGGTCGATTTAACAAATAAAA

>**Smp_153640.2.e3** = SM_V7_ZW:46431829-46431579
ATTTACAAAGAACCAAAAATTCATCGTGTAGACGAATTCGTTATCGATCAAATTCATTTAACGCATTTGTTTG
TCAACAAAGTGAACAAATGGCTGGGACAATACAATTATTTTATTTATATGAAGATAAAAATTCAAAGAATATG
TACAATCTTGATAAATCAAAAGAAAGAAAGAAATCACAAACAGTTGAAAGAATAACCGATGAAATCAAACTTA
GTTTTATAGATCGTCCAGTTGGACATGTAAAG

>**Smp_153640.2.e4** = SM_V7_ZW:46428504-46428447
ATTAAAAATTTAAGTAATTGGGATTTTAACATTTTTCATTTAAAACGAACAAGTTCTA

>**Smp_153640.2.e5** = SM_V7_ZW:46426116-46426021
ATTATACCATTCGTGATATTGGTTTACAAATAATGAATGAATATGATTTATTTCAAAAATTAAAATTAAATTA
TTTCATGATGGCTAGAATATTCA

>**Smp_153640.1.e5** = SM_V7_ZW:46419600-46419500
ACTCTATAGAAGCAGCTTACCACAATTTCAATCCGTATCATACAGCGTTGCATGCAGCCGATGTACTACAAGC
AGTGCATTGTTTTATATCACGAAGTCAA

>Smp_153640.1.e6 = SM_V7_ZW:46405740-46405609
CTTCTGACCATTTTAAGTCCGACAGAAATTTTCGCTAGTCTATTGGCAGCTGCTTTACATGATGCTGATCATC
CCGGTGTGAATCAATCCTATCTAGAGAAGACAGGTGATTTTTTAGTTGATTTATATAAA

>Smp_153640.1.e7 = SM_V7_ZW:46396634-46396543
TCTGTTTCTGTATTGGAAAAACATCATGCAAAATTTGGATTATGCATTCTTCAAGAAAATGGATTGTCTAATG
CATTAGAATTACATGAATG

>Smp_153640.1.e8 = SM_V7_ZW:46394405-46394324
GGAATTCGTGCGTGACTGTTTTCTAAAATTAATTCCAGCTACTGACATTACATATCAAGGAGTATATCAAAAA
CAATTCAAA

>Smp_153640.1.e9 = SM_V7_ZW:46390563-46390492
GATTTAACAAACTATCATATGGCTAATCCATCATTACCATTTACAATATCCGATCGGCTGTTGATCATGCAA

>Smp_153640.1.e10 = SM_V7_ZW:46387570-46387390
ATGGCTTTAAAATGCTCAGATATAAGTAATCCCTGTCGAATTTGGCCAATATGTAAAGAATGGGCAATACGTG
TTTGTTGTGAATTATTTTGTCAGGGTGATAGAGAACGTTTTCAATGGTCATTACAACCTATACCAACAATGGA
TCGCACAAAATTTACTTTAGCGAGAATACAAAATG

>Smp_153640.1.e11 = SM_V7_ZW:46380620-46380331
GATTTATTAGAGATATGGTGAAACCATTATTAACTGGATGGCATGAATTTCTTCAAAATAATCTTACATTGAA
AATTTTACAAAATTTAGATGAAAATTTAAAAAATTGGTTAACTGATTTATCTTTATCGTCGTCGTCGTCAACA
TCATCTTCATCAGGCATGACAAATTATGTTAATCGTCATAATTCATTAGATAGTCAACTGTTATCAACATTAC
CACTTAGTAATAATAATAATAATCAATCTATTTCATCAAATCAAATTAAACCAAAAACTATTAAATCAGAG

>SmPDE7var-a2.e12 maps to SM_V7_ZW:46377004-? (stop codon:46376371-369; 1 nt diff to SmPDE7var-1.e12: exon nt 7, A to G => N478 to D478)
ATATCGGATCAAAAAAGTAAAATTGAATTACAAAAACCATTTATAGGATCCATTCATTTAACTCATTCACCAA
TTCAAGAAATTGAAACAATGAATGCATCTGAATCATTGGATTCTACTACTACTAATAATAATAATTTACATTC
AGGTAAATTACATCAAACAATATTTATTACAACACGTGTTATACGTCGACATTCATTACCGGAAACTCAATTA
GCTATTAGAAAAACATTTAATTTTTCATTATCAAATAAATCAAGTACTGTTGTTCTATTAAAAAATGATACAC
AAAATATATTATCATTACATTCAAGAAATAATAATAATTTACCAGTCGGTAAGATTAAATGTCAATCATCTAC
CTTCAAATCACCATCATCATCACTATCACCATCGTCTACATGTACAGTATCTGCAAATCTATTACAAATGTTA
TATGAAGAATTAAAACATAATAATAAATCAAATTATGTATTATCTAAAATTGATATAAAATCAATTCCAAATA
CAAATTGTTATAAAGATAATAAATTATTAGAACAAAAAGTATTAGATTTAAATTATGATCGTACATTATTACG
ATTTTCTGCACTTGCTCATCGTCGTAGTAGTGCACCAATTACAGAACATTAA

SmPDE8

>Smp_044060.1.e6 = SM_V7_1:?-85254977 (ATG maps to 85254859)
ATGAAATCAGAGACTAGACAGCCGACAATTAAAGAGTCGGTAGACAATGAAATAACATCAACGGATGAACTGA
AAGCCAATGTTATTGACTCAGTCACTGTGTTACATGAGCAAGGAAA

>SmPDE8.e7 = SM_V7_1:85258639-85258682 (longer than Smp_044060.1.e7 by 22 nt)
GAAATTCAGAGAATTTATATCAGTACGAAAAAATCGGAACTCAG

>**Smp_044060.1.e9** = SM_V7_1:85263197-85263270
ATACAAACAATATTGTTAGTTTAAGTGCGTTATTACATTCAAGATGTCCTGATTTTTCATCACCAATGTGTAA
A

>**Smp_044060.1.e10** = SM_V7_1:85266641-85266814
GTCATCGGAATTTTGAACTCAGCACGTGTACGTAGTCCATTACCAGTGGCAAAAGACTTACAGAAAGCTATTA
ATTTAATATGTAGTTCAAATGTATTTGTTGATCAAATAATGAAACCATTAAGTCGGACAAATGATCCTATCAC
AGCAGATCTAATTGAAGGTTTGATTACT

>**Smp_044060.1.e11** = SM_V7_1:85268503-85268569
GGATCAAATCTAGCTAGAGAACCAGAGAACTTATTAAAACTTCGATCTTTGGCTAAATCTCTAAAAG

>**Smp_044060.1.e12** = SM_V7_1:85269762-85269885
GATCCGAAAATGCTTCAACAACACTTTCAACTCTAAAAAATAGTCCAGAAATTGAAGCTTGCCTATCAAATTT
TGATAAATGGGACTTCAATATATTTGATCTTGAACGGATAACTAATAAAAA

>**Smp_044060.1.e13** = SM_V7_1:85271238-85271436
ACCGCTAACTTGTTTAGGTATGAAAATATTGGACAGTTTTAATGCTCTTAGTGTACTACGTATACCTAGTCAA
ATATTAGTCGGTTGGTTAACAGTCATTGAAGAACATTATCATGTTGATAATCCATACCATAATGCTACACATG
CAGGTGATGTTCTACAAGCATCAGCTTATTTTCTACAACATAGTTTAATAAGA

>**Smp_044060.1.e14** = SM_V7_1:85273366-85273496
TCAATATGCACAAATATCGATGAAGTTGCTACACTTTTGGCTGCAATAGTACACGATGTAGACCATCCGGGGA
AAACAAATCCATTTCTTGTGAACAGTAATGATCCGTTAGCCATCCTATACAATGATAT

>**Smp_044060.1.e15** = SM_V7_1:85275597-85275683
TGCTGTATTAGAAAGTCATCATGCGGCAGTTTCATTTGAATTAACTCTTCGATCACCTGACATCAATATTTTT
CAGAATTTGACACG

>**Smp_044060.1.e16** = SM_V7_1:85277588-85277714
AGAAGAATATCGTACAATGCGTAGCTATATTGTTGATATGGTATTAGCTACTGAAATGGTACGACATTTTGAT
ATTGTTACAAAATTTGTAAACACATTGAGTAAACCAATGCTTGCAAAGAATCGA

>**Smp_044060.1.e17** = SM_V7_1:85279800-85280054
CACCATGACCGTTCATCTGTTGGTAGTATGAGTTCTATGGAATCATGTTCCATGGGAATGACAATATCACATT
CTACTTCACCAAGTCCTGGACAAGAACGTATATCTAGTACATTAGAAAATTTATCAACTGCAGAAAATAGAAC
ATTAATTAAACGTTTAATTATTAAATGCTCCGATGTTAATAATCCTACAAGACCATTATCTATATGTAAAGAA
TGGGCAACAAGAATAGCTGAAGAATACTTTTGTCAA

>**Smp_044060.1.e18** = SM_V7_1:85282524-85282653
ACTGAAGAAGAGAAACGACGTAATTTGCCTATTGTTATGCCAAATTTTGATCGACAAACGTGTAATATTTCTC
AGTCACAATTGTCATTTATTGATTTCTTTCTAAAAGGAATGTTTTCAGGATTTGACT

>**Smp_044060.1.e19** = SM_V7_1:85284839-? (stop codon maps to 85284975)
GTGTTTTTCCTATACCTGAACTAATGAATAATCTAGAAAATAATACAACTTACTGGGCTAGTAATATTGACAG
AGAGAAGAAACAACATGGAACTTGTCCAGTGGAATTAAAACCAACAACCATTCATCAAGAATAA

SmPDE9A

>**Smp_197150.1.e1** = SM_V7_1:?-87399415 (ATG maps to 87399347)
ATGGGTAGTGTTATATCAAAGTTAACACCTAAAGTTATCTATTTATTAATTAATGGAAATATTGAAAGA

>**Smp_197150.1.e2** = SM_V7_1:87399464-87399534
ATTTTAATTACATTATCATGTACAAGTTTTGAAATACATGATTTAATTTGTATCCTATCAAATGTTCCAAA

>**Smp_197150.1.e3** = SM_V7_1:87399602-87399679
ATCATCTAATATCATTATAACTGATGTGAATGGATTACATATACCATGTTCTGGTTCAATGTTAGCTAATACA
TATAA

>**SmPDE9A.e4** = SM_V7_1:87402206-87402301 (1 synonymous nt substitution compared to Smp_197150.1.e4: nt 52 of exon: C versus T)
TACACCATATACTGTGACTATTACACAACCAAGTGAACCAAGTGAAATAAGCCTCATTGTTCGAATGTTTGAA
TCAATTATAAAACAAATAAATGA

>**Smp_197150.1.e5** = SM_V7_1:87403777-87403847
TACAATGAAGATTTCTGACTTGAAGAATGAGTTCACCGAACGAATACAACTATTGGAACAACGAGTTATGG

>**Smp_197150.1.e6** = SM_V7_1:87406170-87406269
TTGAATCCGATCGTTATAATGATATAGATGTAATTAAAAAAGAACTTAAACAGTTAAAAATTCAAATTCATGA
AAGAAAAACTGGTTTAACGAATATCGG

>**Smp_197150.1.e7** = SM_V7_1:87408893-87408980
TTCAGAACGTACATATTTAGGGAATATACGATTAAGTAATGATGGTGTCAAAATTTTGGAATTACAGAATTTA
CCCATTTTTGAAAAG

>**Smp_197150.1.e8** = SM_V7_1:87413281-87413355
TATACATTAACACAATCAACTATTGATTTTTTGAAAAAACCAACATTTGATATTTGGCATTGGGAACCGAATG
AA

>**Smp_197150.1.e9** = SM_V7_1:87417327-87417413
ATGTTAGCTCTACTGGAACACATGTATAACGAATTAGGTGTAGTATCAGAATTCAATATTAATCCATTAACAT
TGAAACGTTGGTTG

>**Smp_197150.1.e10** = SM_V7_1:87419773-87419960
TTATCTATACAAGCAAATTATCGTAATAATCCATTTCATAATTTTCGACATTGTTTTTGTGTTGCACAAATGA
TGTATGGAATATTATATCTATGTGGATTGAATAATGATTTTTCACGTGAAGAATTAGGCATCCTATTGACAGC
CGCCGTATGTCATGATTTAGATCATCCAGGATATAGTAACTC

>**Smp_197150.1.e11** = SM_V7_1:87423933-87424089
TTATCAAATAAATGCAAGAACTGAACTGGCCATAAGATATAATGACATATCACCATTGGAAAATCATCATTGT
GCTGTTGCATTCAGTATACTAAATCATCCTGAATTAAACATTTTTGCAAATGTTAATCAAGAAGTATTCCGTC
GAATAAGACAG

>**Smp_197150.1.e12** = SM_V7_1:87428698-87428814
GGTATGACAAGTTTAATTCTATCTACTGATATGGCAAGACATGGTGAAATATTAGAAACAATGAGAAGACATT
TAGAAGAAGGTTTTTCAATGAATAAAAAAGAACATCGTGAAACT

>**Smp_197150.1.e13** = SM_V7_1:87429574-87429678
TTTAAAATGGTTCTTATTAAATGTTGTGATATATCAAATGAAGTAAGACCATTATCAGTTAGTGAACCATGGG
TTGATTGTTTATTGGAAGAATATTTTAATCAG

>**SmPDE9A.e14** = SM_V7_1:87432927-87433055 (1 synonymous nt substitution compared to Smp_197150.1.e14: nt 36 of exon: A versus G)
TCTGATCGAGAGAAATTAGAAGGTTTACCAGTAGCACCATTTATGGATCGTGAAAAAGTCACTAAACCAACAG
CACAAATTGGTTTCATTAAATTTGTTCTCATTCCAATGTTTCAAACAGTGGCAAGT

>**Smp_197150.1.e15** = SM_V7_1:87434620-? (stop codon maps to 87434742)
GTTTATCCAATTATTGATGAACTTATGGTGACACAATTGAAATCAGCATTAGAACGTTATGAAAAGATGTTAG
CTGAAGAAGAAGAAACAAAACGTAATTTACAATTGAATGAAGCAGATTAA

SmPDE9B-a1

>**Smp_146120.1.e1** = SM_V7_2:?-13183358 (ATG maps to 13183325)
ATGAATGCGACGAAGTGTGAAGAAGATATTACCT

>**Smp_146120.1.e2** = SM_V7_2:13254488-13255610
CTGATCATAAAATATCAAACATCACCATTTCACAAAACAATACAAATGATAATACTAGAACCATATGCAAACC
GGCAATTCAAACTACAAAGACTACAACAACATTACCAAGGAAACTGAATGCAGATAATGATGATAATAATACT
AATAGTTTCTTATCCTTATATACCAGATGCATTAAAAATCCAAATAGACGGAAGAAAAAACAGTTAGATAATA
ATAATAATAATAATAATAACAGCAATCATAATCATCAGTCACATTTTTGTTTAAATTTTTGCCGAAATTGTTC
ATCAAATACACCTTCTTCTTCATCGTCATCATCATCATCATCAACCGCTACAACTGAAGCGGGGGCTTCGGTA
GCGGTTGCAGCAACAACAGGACGTAAAGAATCGAATAGTTTTTCGCTTCCATTTCCACTGGATAATAAAAGTC
ATGACAGTTTAATTAGTTACTCGTCAAAAATTACTAATAACACTAATTTTACTAATGATATTTCCAGTCCTGG
AACTAAAGTTACACTTACTCCTAATCATACAAATAACATTATAATGTCTGTATCCAATTGTTCAAATCAATTT
AATAGCATTTCAGTTGATCCTGATAAAAAGGATAATCATAAAATTGATGCTCAAAACACATCTGTACATATAA
ATAAAAGGCAAACAGCTGATAAATTTCATTCCAATACTACAGAGTACCAAAGTAAATCTAACATGACCACTAC
TGTTATGACATCTATACCTACAACAACTACTATTGAACCTATAAATGTTCATAAGAAGATTAATCCATCTTTA
ATGATCGCTGATAATTCAGCATACTTTAATGATAATCATGAAAATTTATTAGAAATATCTAAACTTCCAATCT
ATGACTCAACAAAACTTTGTATTAAATGTCAAAAGAATATTCAATTAGTTAGTAAAGATTATGAACATATACT
ACTTTTAACAAATAATCAGGGAAAACATGAAAAGATCACTGTTAATAATAATAGCATTATTAACAATAATAAT
AATGGGAATGGGTACATGGAGCATACTACTGAAATGATTGATGACATCAAGTCCAAAAAAATTAAAGAAATGG
AGTCAGATATTGAAGAATACGTCAAACA

>**Smp_146120.1.e3** = SM_V7_2:13268561-13268912
TGTTCAACATATTTTTGATCACATAAATCTGACAAAGGATCAATTCTGTCAAACTGATTCAAAAAGTAATTAT
ATTATTGATAATAAACAATTATGGGATGCATTTAATACAAGTTATGCAAATCAAGAAAAAATATTTCGACCTT
CACATAATGATATAAATATATATCATTTAGATGATGATATGAATAATGGTGATGTTAATGATGATAATGATGA
AGAGATCATAGAAGCTCATTATGATAATGTTCATAGTTACCGTAAATGTAACCATTTCTCCGTTGATAAAATG
GATAAAAAACGAATTTCAAAAATCTATAGACAACTTCATAATTTACGTTGTCAAGTTGAA

>**Smp_146120.1.e4** = SM_V7_2:13270181-13270344
TCGTTCTCCTATCTCAGTTGGCTTGGATTAACCGCAGAACAACCCCCAACACAAAAAGTTCTAGTTCCTGGTT
TTAATGCACCAGCTCCAAATCCTCAAATGCACTTAATTCGTCGATCTGATGCAGATAGTCGAAGAAATATACA
AGAATTTAAACTTTTATG

>Smp_146120.1.e5 = SM_V7_2:13276604-13276841
TAAAGAACCTGTATCTAAAGAAGATTTAATTGAATTACGCTCATCTACTTTTAATAATTGGTCTAGAACAGAT
GCTCAATTAATACGTTTAGTTCGGGAAATGTTTCAAGAACTTGGATTTATTGAGCATTACAATATACAATTAC
ATCAATTAGATTTATGGCTTACTGATATTTATAGACGTTATAATCGTGTTCCATTTCATAATTATAAACATGC
ATTTATGGTGACACAAATG

>Smp_146120.1.e6 = SM_V7_2:13277894-13278016
TGCTATGTTTTAATCTGGGGTGGAAATTTGACCAATCTACTTGACATAGATGATCAACTTATTTTGATTGTAT
CGGCCATCTGTCATGATTTAGATCATCCAGGATTCAATAATGCTTATCAG

>Smp_146120.1.e7 = SM_V7_2:13284531-13284691
ATCAATGCTGGAACCGTGTTAGCGATGAGATACAATGATCAAAGTCCATTAGAAAATCATCATACGGCTGTCG
CATTTGATCTATTGAGTCATAAAGAAGTCGATCCGTTCTCACATCTTTCGACTACTACAAGACAACGAATTCG
CAAAGGAGTGATTAG

>Smp_146120.1.e8 = SM_V7_2:13286917-13287169
ATGTATATTGGCTACTGATATGTCACGTCATAATGAAATATTAGATGAATTTAATCGTCAAGTTTTGACTGAT
TTAAATGCTGCATGGGAAATTGATCCAAATACAAAAAAACCTACATGGGTTATGAATAAAACACAAAAAGATC
TTGTTATGGTAATTATTTTGAAAATATCTGATATATCCAATGAAGCACGTCCTTTAAATGTTGCTGGTCCATG
GATCAATCGATTATTAGCAGAATTTTTTCATCAA

>Smp_146120.1.e9 = SM_V7_2:13291441-13291587
AGTGATTATGAAAAACTTGTTGGTCTTCCAGTTGCTCCATTTATGGATCGACATAAAGTGACTAAATCAGCTA
GTCAATGCGGTTTTATTCGTTTTGTTATTTTGCCTCTATTTGAATCATTAGCTAAATTATTACCAGAAGTTAA
A

>Smp_146120.1.e10 = SM_V7_2:13294060-? (stop codon maps to 13294216-218)
CCAATTATTGTTCAACCAGCCTTAGAACAATTGGCCTACTATACAGATTTACACAATAATGAAGAAAAGAAAA
CCAATACTGAAAATCAGAAATCAAATACTAATGAACATCAAAATGGTAACAATAATAATCATAACGAAAAAGA
ACATAGCAAATAA

SmPDE9B-a2

>Smp_146120.1.e1 = SM_V7_2:?-13183358 (ATG maps to 13183325)
ATGAATGCGACGAAGTGTGAAGAAGATATTACCT

>SmPDE9B-a2.e1 = SM_V7_2:13254488-13254715 + C + 13254723-13255610
CTGATCATAAAATATCAAACATCACCATTTCACAAAACAATACAAATGATAATACTAGAACCATATGCAAACC
GGCAATTCAAACTACAAAGACTACAACAACATTACCAAGGAAACTGAATGCAGATAATGATGATAATAATACT
AATAGTTTCTTATCCTTATATACCAGATGCATTAAAAATCCAAATAGACGGAAGAAAAAACAGTTAGATAATA
ATAATAATACCTAACAGCAATCATAATCATCAGTCACATTTTTGTTTAAATTTTTGCCGAAATTGTTCATCAAA
TACACCTTCTTCTTCATCGTCATCATCATCATCATCAACCGCTACAACTGAAGCGGGGGCTTCGGTAGCGGTT
GCAGCAACAACAGGACGTAAAGAATCGAATAGTTTTTCGCTTCCATTTCCACTGGATAATAAAAGTCATGACA
GTTTAATTAGTTACTCGTCAAAAATTACTAATAACACTAATTTTACTAATGATATTTCCAGTCCTGGAACTAA
AGTTACACTTACTCCTAATCATACAAATAACATTATAATGTCTGTATCCAATTGTTCAAATCAATTTAATAGC
ATTTCAGTTGATCCTGATAAAAAGGATAATCATAAAATTGATGCTCAAAACACATCTGTACATATAAATAAAA
GGCAAACAGCTGATAAATTTCATTCCAATACTACAGAGTACCAAAGTAAATCTAACATGACCACTACTGTTAT

GACATCTATACCTACAACAACTACTATTGAACCTATAAATGTTCATAAGAAGATTAATCCATCTTTAATGATC
GCTGATAATTCAGCATACTTTAATGATAATCATGAAAATTTATTAGAAATATCTAAACTTCCAATCTATGACT
CAACAAAACTTTGTATTAAATGTCAAAAGAATATTCAATTAGTTAGTAAAGATTATGAACATATACTACTTTT
AACAAATAATCAGGGAAAACATGAAAAGATCACTGTTAATAATAATAGCATTATTAACAATAATAATAATGGG
AATGGGTACATGGAGCATACTACTGAAATGATTGATGACATCAAGTCCAAAAAAATTAAAGAAATGGAGTCAG
ATATTGAAGAATACGTCAAACA

>**Smp_146120.1.e3** = SM_V7_2:13268561-13268912
TGTTCAACATATTTTTGATCACATAAATCTGACAAAGGATCAATTCTGTCAAACTGATTCAAAAAGTAATTAT
ATTATTGATAATAAACAATTATGGGATGCATTTAATACAAGTTATGCAAATCAAGAAAAAATATTTCGACCTT
CACATAATGATATAAATATATATCATTTAGATGATGATATGAATAATGGTGATGTTAATGATGATAATGATGA
AGAGATCATAGAAGCTCATTATGATAATGTTCATAGTTACCGTAAATGTAACCATTTCTCCGTTGATAAAATG
GATAAAAAACGAATTTCAAAAATCTATAGACAACTTCATAATTTACGTTGTCAAGTTGAA

>**Smp_146120.1.e4** = SM_V7_2:13270181-13270344
TCGTTCTCCTATCTCAGTTGGCTTGGATTAACCGCAGAACAACCCCCAACACAAAAAGTTCTAGTTCCTGGTT
TTAATGCACCAGCTCCAAATCCTCAAATGCACTTAATTCGTCGATCTGATGCAGATAGTCGAAGAAATATACA
AGAATTTAAACTTTTATG

>**Smp_146120.1.e5** = SM_V7_2:13276604-13276841
TAAAGAACCTGTATCTAAAGAAGATTTAATTGAATTACGCTCATCTACTTTTAATAATTGGTCTAGAACAGAT
GCTCAATTAATACGTTTAGTTCGGGAAATGTTTCAAGAACTTGGATTTATTGAGCATTACAATATACAATTAC
ATCAATTAGATTTATGGCTTACTGATATTTATAGACGTTATAATCGTGTTCCATTTCATAATTATAAACATGC
ATTTATGGTGACACAAATG

>**Smp_146120.1.e6** = SM_V7_2:13277894-13278016
TGCTATGTTTTAATCTGGGGTGGAAATTTGACCAATCTACTTGACATAGATGATCAACTTATTTTGATTGTAT
CGGCCATCTGTCATGATTTAGATCATCCAGGATTCAATAATGCTTATCAG

>**Smp_146120.1.e7** = SM_V7_2:13284531-13284691
ATCAATGCTGGAACCGTGTTAGCGATGAGATACAATGATCAAAGTCCATTAGAAAATCATCATACGGCTGTCG
CATTTGATCTATTGAGTCATAAAGAAGTCGATCCGTTCTCACATCTTTCGACTACTACAAGACAACGAATTCG
CAAAGGAGTGATTAG

>**Smp_146120.1.e8** = SM_V7_2:13286917-13287169
ATGTATATTGGCTACTGATATGTCACGTCATAATGAAATATTAGATGAATTTAATCGTCAAGTTTTGACTGAT
TTAAATGCTGCATGGGAAATTGATCCAAATACAAAAAAACCTACATGGGTTATGAATAAAACACAAAAAGATC
TTGTTATGGTAATTATTTTGAAAATATCTGATATATCCAATGAAGCACGTCCTTTAAATGTTGCTGGTCCATG
GATCAATCGATTATTAGCAGAATTTTTTCATCAA

>**Smp_146120.1.e9** = SM_V7_2:13291441-13291587
AGTGATTATGAAAAACTTGTTGGTCTTCCAGTTGCTCCATTTATGGATCGACATAAAGTGACTAAATCAGCTA
GTCAATGCGGTTTTATTCGTTTTGTTATTTTGCCTCTATTTGAATCATTAGCTAAATTATTACCAGAAGTTAA
A

>**Smp_146120.1.e10** = SM_V7_2:13294060-? (stop codon maps to 13294216-218)
CCAATTATTGTTCAACCAGCCTTAGAACAATTGGCCTACTATACAGATTTACACAATAATGAAGAAAAGAAAA
CCAATACTGAAAATCAGAAATCAAATACTAATGAACATCAAAATGGTAACAATAATAATCATAACGAAAAAGA
ACATAGCAAATAA

SmPDE9C

>**SmPDE9C.e2** = SM_V7_1:?-11128212 (additional ATG at the 5'end of the CDS compared to Smp_342020.1.e2)
ATGATGTTTAAACGATTAATTCGTTGTCATGTGAAATCATCTCGTACACCACCCAATAAAGATGGTACAAATA
ATAAAATTCACTTACCAACTAAATGTACTACGTGGTTATTTTCAACCATTTCCTCTTCATCTAAAATCTCAAC
TACCAGTGACATTGAAGCAAGTAAAAGCACAGAAACTTGTTTAGTAGATAACTCTTCTAAAACAAACAATTGT
AATGAAACATGTAATTTAATTAACACTCAACATCCTAATGATAGTCAACGAATCTCTTGCAATCCTTACTGCC
CTGATCCAGAAGGTATAAAACTACCTTTTATACATTTCACTAAAGTTAGGAATCAATTTCTAGCTATTCG

>**SmPDE9C.e3** = SM_V7_1:11126952-11126715 (69 nt shorter than Smp_342020.1.e3)
ATCTCAATCAATATCTTCATCTATAAAAGAACAGTTAAAATCTCATTCATTTAATAATTGGTTATATTCAGAT
GCTGAATTAATTAATTTTGTTAAATTTATGTTTGTCGATTTAAATCTACCGGAATTATGTCATTTCTCTATTG
ATACACTTGAAAATTGGATATTTTCAACTTATTCACGATATAACAATGTACCATTTCATAATTTTAAACATGC
ATTTATGGTAACTCAAATG

>**Smp_342020.1.e4** = SM_V7_1:11106993-11106871
ATGTATTGTATCATTAAAATGGTAAATTTACCATTGTATTTATCCTCAGTGGATTTACTGATTCTATTATTCT
CCGCTTTGTCACATGATCTTGATCATCCAGGTTTTACAAACTCTTACCAG

>**Smp_342020.1.e5** = SM_V7_1:11096351-11096188
ATTAATTCTGGCACTTGGCTTGCTCTACGTTACAACGATATATCACCATTGGAGAATCATCATTGTATGACAG
CGTTCGATCTTATCACCAATAATCCAACGGCAAATATTATCAGTGGATTAACGCCGAATGAATCACGTCATTT
TCGTAGATCTGTAATAAG

>**Smp_342020.1.e6** = SM_V7_1:11094359-11093684
ATGTATTTTAAGCACAGATATGGCAATTCATTCAGAATGTCTATCACAATTTCAAGTTTTACGAAAACAAGTC
TATTTGAATTGTCAGTCATTATCTATAGATATGAGTAGTAGTAGTATTAGTAGTATAAAACATAATCATCAAA
ATATTGATCATTCTTGTTGTAATAATAATAATCAACAAAAGAAATTGAAAAATCCTGATTCAATATTTCCTTC
TCTTCAATTATCTTCATCATATATTGGTTATTCATCGCCATCGCCTACACAACAACAGCCTACACTAACTACT
TCCCATGATCATTATCATCGTCATCAAGTTAACCATAATGACAATGATGAAACAGGGGTGGACAGACAAAATC
GATCAGAAAAATATGAACAATATAATTCATGTATCAATCAACATGTTAATAATAATAATAATAACGTTGTTAA
TTTTGGCGGTGATAATGACAGTGATCACAATGGTGATGATATTAATAACAATGATTATGGTCATATTAATTCA
TCACTGATCCAGTCTTCATTAATTTCATTAATTAATCAAGAACCAGAATATTTATTAAGACTTTTAATGATAC
TGTTGAAAGTATGCGATATATCGAATGAAATTCGTTCACCATTAGTTGCTGATGCCTGGGTGGATTGTTTATT
CAACGAATTTTTTCTACAG

>**Smp_342020.1.e7** = SM_V7_1:11090036-11089890
GCTGCAGCAGAAAAACAAGCTGGTCTTCCTGTTGCTCCTCATATGGATCCAGATCTTGTAGTGAAATCCAATA
GTCAGTTAAATTTTTTACATAGTATTCTAATACCATTAGTGAAGGAATTGACGTATATCTTTCGTGAATTACA
C

>**SmPDE9C.e8** = SM_V7_1:11088359-? (stop codon maps to 11088146-144, Smp_342020.2.e8 extended by at least 30 nt)
GTACTTTTGGAATCTGCGCATAGACGATCAGAGCATTTCTTTCAGATTAAACAATATGAATTAGCTCAACAAG
TGGTGGATAGTAACTGTTGTTCAACAACAGTAACCACATCAACATCGTCAACATTACCAATAACAACAATTAC
TAGTACTTCTGTATGTCATGTCAATAAGTCTAATGTACGGGTCAGTTTATTATTATTATTATTTAATTGA

SmPDE11-a1

>**Smp_179590.1.e1** = SM_V7_ZW:?-10481909 (ATG maps to 10482694)
ATGTCCAGTTTAGTGAGAATGTGTGAACTTTGTGGTGGTCATATTGGTGAACAGTCAGAATTGTCCTTCGAAG
ACATGGTCACTAACTGGTTAGATGAGAATCCTGAATTTACTTTCAAGTACTTTGTCAAATCCGCATCCCCAAG
CATGGTTGAAGCCTGGGCTAATGGACGTAATCATGGTGAATATGATTGCCTCTTTGATAACTCAATAACTGTT
ATGGACGAGCACAATGATAAAAATGATGATAAGACTCCTGTTACCACTTCTCTATCACTACCTATACGTAAGA
TAAGCAGCCAGGATTTGGAATTAACCTATGACAAGCGTATTTTATCTTCCAACGAAGATGGAAAGCCCACATT
TATAAACTCTGTGTTCTTTCCTTTCCCTTCCAATGAACACATCGATTCAAATACTAGTTCTGTTCGTCAAACT
GTGGCTTCGCCAAGTCGTCCGACACATCTAACTGAGAGAGATTTGATAAGTGAACTTGCTTTAGATATTTGTC
GTGAACTTGACGTGACATCTTTATCCTTTAAAATTGTGCAGAATGTTTGTAGACTAATCAACGCTGATCGTGG
ATCGTTTTTCCTTGTGGAAAAGTCGCGTTCAACCGGTGAGGATGTTCTTGTCAGCAAACTTTTTGACATTACT
CCTGAGTGTATATTTGATGATGTTTTACAGCGATGTTCCTCTAATCACATCATTGTTCCTTTTAATGTTGGTG
TAACCGGCTACGTCGCACGAACTGGAGATTACGCAAATATTCCGGATGCATATGCC

>**Smp_179590.1.e2** = SM_V7_ZW:10480954-10480634
GATCCTCGATTTGATGATTCCGTAGATCGTGTAACGGGATATAAAACGCGCTGCCTTTTATGCATGCCTATAA
AAAATGTTGATGGAAAAGTGTTAGGCGTTGCACTGGTTATCAATAAGAAAGTTCCTTCTGATCAGCATCATGA
TCAAAGCATTAACTCAGTTCAACCTAGTAGTTGTGAGAGTGAATCAATCAGTAAACATGCTTCATTCACTGAA
GAAGATGTAAAGATATTTCAATCATATGTTACATTCTGTGGTATTGGATTACACAATGCACAAATTTATGAGC
AGAGTCGTTTAGAAACTTACCGTAACCAA

>**Smp_179590.1.e3** = SM_V7_ZW:10476534-10476370
GTACTTTTGGAACTTGCACGAATTATATTTTCAGAACAACTTGATATAACTCGTCTTATTTATTCAGTATTAT
CACATACAATTTGTTTACTACAATGTCAACGTTGTCAATTACTTTTAGTGAAAACAACTTCATCTATGTCATC
CTATTCTTCAATTGATGAG

>**Smp_179590.1.e4** = SM_V7_ZW:10463193-10463102
ATGGGACCATTTCATGATCATTTCTCACAAATTTTTGAATTAGCGTGGAACGAAAAATCGGATTCGCCTGATG
TTAAGAAAAAAAAAGCATAG

>**Smp_179590.1.e5** = SM_V7_ZW:10451807-10451744
TATCGACGAAGCTCGATTCCCTGTTCAGTTAGATTTGGCTATTCACGTATTACAAACTGGTGAA

>**Smp_179590.1.e6** = SM_V7_ZW:10448517-10448422
TCTCTTCATGTCAATATCAATGGTACCACTACTATTTCGAATAATAATAATAAAAATAAAAATAATAATAATG
AATACAAAAAGATTGACGAAACA

>**Smp_179590.1.e7** = SM_V7_ZW:10447787-10447282
CTCGAAGAAGATTTGGATCCAGTTTGGAGAAGTCGTTCCGTTCTCTGTATGCCAATTAAACATTCCGACGGTA
AAGTATTAGCTGTATGCATAATAACAAATAAGTCAACTGTTGATTTAAGAATAAATAATAATAATTTCAGTCA
ACAAGTTACGCGTCATTTCGATTTCAAACCAGTTACAGATAATGTTCAATGTTCATCAAATGATAATCTATCA
TCTAAAGAACCCGTTACAATGTCGACATCAATTAACGATTGGTCTGGAATATTTACTTATTCGGATGAATTTT
TATTTGAAGCATTCGCTTTATTTGTTGGCTTGGGTATTTCAAATAGTCAACTGTATGAAAAAGCTATACGAAG
TGCAGCAAAACAAAAAGTTATCATGGATGTTTTGTCGTATCATGCTACAGCGCCGACATCTGAAGCTAAACGC
TTAGCTACATCATTAATACCAACTATGAGATTTTATCATTTAGATAAATTTTCATTTACTGATGTACG

>Smp_179590.1.e8 = SM_V7_ZW:10445340-10445148
ATTATCAGATGAAGATACTCTGAAAGCATGTATACGAATGTTTCAAGAAATGAATTTTATGAAATCAATACAT
TTCGATCAGTTATCATTTGCTCGTTGGCTATTATCTGTACGTAAAAACTATCGTGAAGTTACTTATCACAATT
GGCGTCATGCATTCAATGTGACTCAGACAATGTTTTGCATATTACTA

>Smp_179590.1.e9 = SM_V7_ZW:10436666-10436469
AAAGGTGATTTTCAGTCAGTATTTACAGATCTTGAATGTTTAGCATTGTTAACTGCTTGTCTATCTCATGATA
TCGATCATCGTGGTACAGATAATCAATTTCAAATTAAAACAATGTCTCCATTAGCTAAATTATATTCTACATC
AGTGCTTGAACATCACCATTTCAATCAGTTCATGATGATACTATCTATAAAG

>Smp_179590.1.e10 = SM_V7_ZW:10435848-10435582
GGTAATAACTTTTTATGCAATCTAAACAGATCAGAATATGATACTGTTGTGAAATTAATTAGAGAAGCTATAT
TAGCTACGGATCTATCAAGATATTTTGCTAGACTACCGAAATTTCAACAAACTTTGCATCATTTGAAAGAAAT
TAATGATCAATCTACAAATTTATGGCGAAATGATCGCGAACAACGTTTATTACTTGGCTGTATGTTTATGACA
GCTTGTGATGTTTCAGCCATAACAAAACCATGGCCAGTTCAAAAATTG

>Smp_179590.1.e11 = SM_V7_ZW:10434737-10434663
ACAGCTGAAATGGTTGCCAATGAATTTTTTGAACAAGGTGATTTGGAAAAAGAACGACTGAACGTAACACCAG
CT

>Smp_179590.1.e12 = SM_V7_ZW:10425951-10425868
GCACTAATGGATCGTGAACGCAGTAATGAACTTCCAAAATTACAAGTCAGCTTCATTGACTCAATTTGTGTGC
CGATTTACGAG

>Smp_179590.1.e13 = SM_V7_ZW:10424530-? (stop codon maps to 10424132)
GCTATTGTACAAGTTTCTCCAAATTTTGAACCATTACTAAAAGGCTGTAAACGTAATCGTACATGTTGGCTTA
TTCTATCTGAAAACGGTGAAGTCGATCATTCGCTATATGGATTAAACGATGAATCAGAGACTATACCAGGAAC
ATCAACTGAAACTACAGCAAAATCAATCACAACGACAACAGCGGAAGGATCAACACAACTAGAAAATGTAATC
ACATCCACATCTAATATTATTACATCGTTAGGTACATTGAATCCAGATTCAGCTGAACGTATTGCAAAAAGTA
AACCATTTACATCATCAAATATTCGTAGAGCTTCTGTATCATCAATGACTACAGTTGGGGGCCAGGAACCACC
GATACAGTGTCAATCATCTGTTGAATCAAAATAA

SmPDE11-a2

>SmPDE11-a2.e1 maps to SM_V7_ZW:10482694-10481909; nt 15+16 differ: GA->AG => R6->G6; nt 79 differs: G->A => V27->I27
ATGTCCAGTTTAGTAGGAATGTGTGAACTTTGTGGTGGTCATATTGGTGAACAGTCAGAATTGTCCTTCGAAG
ACATGATCACTAACTGGTTAGATGAGAATCCTGAATTTACTTTCAAGTACTTTGTCAAATCCGCATCCCCAAG
CATGGTTGAAGCCTGGGCTAATGGACGTAATCATGGTGAATATGATTGCCTCTTTGATAACTCAATAACTGTT
ATGGACGAGCACAATGATAAAAATGATGATAAGACTCCTGTTACCACTTCTCTATCACTACCTATACGTAAGA
TAAGCAGCCAGGATTTGGAATTAACCTATGACAAGCGTATTTTATCTTCCAACGAAGATGGAAAGCCCACATT
TATAAACTCTGTGTTCTTTCCTTTCCCTTCCAATGAACACATCGATTCAAATACTAGTTCTGTTCGTCAAACT
GTGGCTTCGCCAAGTCGTCCGACACATCTAACTGAGAGAGATTTGATAAGTGAACTTGCTTTAGATATTTGTC
GTGAACTTGACGTGACATCTTTATCCTTTAAAATTGTGCAGAATGTTTGTAGACTAATCAACGCTGATCGTGG
ATCGTTTTTCCTTGTGGAAAAGTCGCGTTCAACCGGTGAGGATGTTCTTGTCAGCAAACTTTTTGACATTACT
CCTGAGTGTATATTTGATGATGTTTTACAGCGATGTTCCTCTAATCACATCATTGTTCCTTTTAATGTTGGTG
TAACCGGCTACGTCGCACGAACTGGAGATTACGCAAATATTCCGGATGCATATGCC

>**Smp_179590.1.e2** = SM_V7_ZW:10480954-10480634
GATCCTCGATTTGATGATTCCGTAGATCGTGTAACGGGATATAAAACGCGCTGCCTTTTATGCATGCCTATAA
AAAATGTTGATGGAAAAGTGTTAGGCGTTGCACTGGTTATCAATAAGAAAGTTCCTTCTGATCAGCATCATGA
TCAAAGCATTAACTCAGTTCAACCTAGTAGTTGTGAGAGTGAATCAATCAGTAAACATGCTTCATTCACTGAA
GAAGATGTAAAGATATTTCAATCATATGTTACATTCTGTGGTATTGGATTACACAATGCACAAATTTATGAGC
AGAGTCGTTTAGAAACTTACCGTAACCAA

>**Smp_179590.1.e3** = SM_V7_ZW:10476534-10476370
GTACTTTTGGAACTTGCACGAATTATATTTTCAGAACAACTTGATATAACTCGTCTTATTTATTCAGTATTAT
CACATACAATTTGTTTACTACAATGTCAACGTTGTCAATTACTTTTAGTGAAAACAACTTCATCTATGTCATC
CTATTCTTCAATTGATGAG

>**Smp_179590.1.e4** = SM_V7_ZW:10463193-10463102
ATGGGACCATTTCATGATCATTTCTCACAAATTTTTGAATTAGCGTGGAACGAAAAATCGGATTCGCCTGATG
TTAAGAAAAAAAAAGCATAG

>**Smp_179590.1.e5** = SM_V7_ZW:10451807-10451744
TATCGACGAAGCTCGATTCCCTGTTCAGTTAGATTTGGCTATTCACGTATTACAAACTGGTGAA

>**SmPDE11-a2.e6** maps to SM_V7_ZW:10448517-10448422; but nt 46 differs: A->T => N492->Y492
TCTCTTCATGTCAATATCAATGGTACCACTACTATTTCGAATAATtATAATAAAAATAAAAATAATAATAATG
AATACAAAAAGATTGACGAAACA

>**Smp_179590.1.e7** = SM_V7_ZW:10447787-10447282
CTCGAAGAAGATTTGGATCCAGTTTGGAGAAGTCGTTCCGTTCTCTGTATGCCAATTAAACATTCCGACGGTA
AAGTATTAGCTGTATGCATAATAACAAATAAGTCAACTGTTGATTTAAGAATAAATAATAATAATTTCAGTCA
ACAAGTTACGCGTCATTTCGATTTCAAACCAGTTACAGATAATGTTCAATGTTCATCAAATGATAATCTATCA
TCTAAAGAACCCGTTACAATGTCGACATCAATTAACGATTGGTCTGGAATATTTACTTATTCGGATGAATTTT
TATTTGAAGCATTCGCTTTATTTGTTGGCTTGGGTATTTCAAATAGTCAACTGTATGAAAAAGCTATACGAAG
TGCAGCAAAACAAAAAGTTATCATGGATGTTTTGTCGTATCATGCTACAGCGCCGACATCTGAAGCTAAACGC
TTAGCTACATCATTAATACCAACTATGAGATTTTATCATTTAGATAAATTTTCATTTACTGATGTACG

>**Smp_179590.1.e8** = SM_V7_ZW:10445340-10445148
ATTATCAGATGAAGATACTCTGAAAGCATGTATACGAATGTTTCAAGAAATGAATTTTATGAAATCAATACAT
TTCGATCAGTTATCATTTGCTCGTTGGCTATTATCTGTACGTAAAAACTATCGTGAAGTTACTTATCACAATT
GGCGTCATGCATTCAATGTGACTCAGACAATGTTTTGCATATTACTA

>**Smp_179590.1.e9** = SM_V7_ZW:10436666-10436469
AAAGGTGATTTTCAGTCAGTATTTACAGATCTTGAATGTTTAGCATTGTTAACTGCTTGTCTATCTCATGATA
TCGATCATCGTGGTACAGATAATCAATTTCAAATTAAAACAATGTCTCCATTAGCTAAATTATATTCTACATC
AGTGCTTGAACATCACCATTTCAATCAGTTCATGATGATACTATCTATAAAG

>**Smp_179590.1.e10** = SM_V7_ZW:10435848-10435582
GGTAATAACTTTTTATGCAATCTAAACAGATCAGAATATGATACTGTTGTGAAATTAATTAGAGAAGCTATAT
TAGCTACGGATCTATCAAGATATTTTGCTAGACTACCGAAATTTCAACAAACTTTGCATCATTTGAAAGAAAT
TAATGATCAATCTACAAATTTATGGCGAAATGATCGCGAACAACGTTTATTACTTGGCTGTATGTTTATGACA
GCTTGTGATGTTTCAGCCATAACAAAACCATGGCCAGTTCAAAAATTG

>**Smp_179590.1.e11** = SM_V7_ZW:10434737-10434663
ACAGCTGAAATGGTTGCCAATGAATTTTTTGAACAAGGTGATTTGGAAAAAGAACGACTGAACGTAACACCAG
CT

>**Smp_179590.1.e12** = SM_V7_ZW:10425951-10425868
GCACTAATGGATCGTGAACGCAGTAATGAACTTCCAAAATTACAAGTCAGCTTCATTGACTCAATTTGTGTGC
CGATTTACGAG

>**SmPDE11-a2.e13** maps to SM_V7_ZW:10424530-10424132, but nt 105 of exon differs, G>A =>
Ala1012 > Thr1012
GCTATTGTACAAGTTTCTCCAAATTTTGAACCATTACTAAAAGGCTGTAAACGTAATCGTACATGTTGGCTTA
TTCTATCTGAAAACGGTGAAGTCGATCATTCACTATATGGATTAAACGATGAATCAGAGACTATACCAGGAAC
ATCAACTGAAACTACAGCAAAATCAATCACAACGACAACAACGGAAGGATCAACACAACTAGAAAATGTAATC
ACATCCACATCTAATATTATTACATCGTTAGGTACATTGAATCCAGATTCAGCTGAACGTATTGCAAAAAGTA
AACCATTTACATCATCAAATATTCGTAGAGCTTCTGTATCATCAATGACTACAGTTGGGGGCCAGGAACCACC
GATACAGTGTCAATCATCTGTTGAATCAAAATAA
