# Supplementary material for: Cloning and functional complementation of ten Schistosoma mansoni phosphodiesterases expressed in the mammalian host stages
Source: PLoS Negl Trop Dis. 2020 Jul 30;14(7):e0008447. doi: 10.1371/journal.pntd.0008447 (PMC7430754; doi:10.1371/journal.pntd.0008447)
Supplement: S2 File — (PDF) [file pntd.0008447.s014.pdf]

S2 File. Amino acid sequences

>**SmPDE1-sv1** - S. mansoni SmPDE1, strain CD splice variant 1, translated CDS
MGSCASTGVSQAGSPAKSQLTNGIQQTSFNIQEMNQTEIRNINEEGDAMAGQEKKTDNPR
SNYVLSGIDLLDYGVGLKDIPLRTSDTNMTTDEELDMECALSGVSQFFYKNLTTCTLGQY
AQIPNDTDMLRSVNTPESLRACYVRMRHIYRMIETDRMGKATLMKNIQYAINVMENAYIA
EKRRIREEEEDLSEAATEYVPDEVRNWLASTFTRTVQSVGIGDQKPRFRSVANAIRAGIF
VERIYRRMSSCSNLIVPPNVLLFLKTGLDTWNFDVFGLNEASENHALKFVAFELLHKYNL
INKFQINSTALESLLIQLETGYSKYSNPYHNLVHAADVMQTCHMIIFMNDLRNWLNDLDI
FAVLFAAVIHDYEHTGTTNNFHIATRSELALIYNDRGVLENHHVSAVFRLMQEEEFSILT
GLEADQYKEFRQLVIDMVLCTDMSLHFQQIKNMKTMISMPESIDKTKALSLIVHCADISH
PAKEWALHEQWSDILCEEFFRQGDRERELNLPISPLCDRNTVVVPQSQIGFIDFIVEPSF
QVLGDMIERIVNPTQTEGVLPTDTTSPKPKSSDQETVGEQVVPRPWVEHFKENKESWSKK
LPPKT

>**SmPDE1-sv2** - S. mansoni SmPDE1, strain CD splice variant 2, translated CDS
MGSCASTGVSQAGSPAKSQLTNGIQQTSFNIQEMNQTEIRNINEEGDAMAGQEKKTDNPR
SNYVLSGIDLLDYGVGLKDIPLRTSDTNMTTDEELDMECALSGVSQFFYKNLTTCTLGQY
AQIPNDTDMLRSVNTPESLRACYVRMRHIYRMIETDRMGKATLMKNIQYAINVMENAYIA
EKRRIREEEEDLSEAATEYVPDEVRNWLASTFTRTVQSVGIGDQKPRFRSVANAIRAGIF
VERIYRRMSSCSNLIVPPNVLLFLKTGLDTWNFDVFGLNEASENHALKFVAFELLHKYNL
INKFQINSTALESLLIQLETGYSKYSNPYHNLVHAADVMQTCHMIIFMNDLRQNWLNDLD
IFAVLFAAVIHDYEHTGTTNNFHIATRSELALIYNDRGVLENHHVSAVFRLMQEEEFSIL
TGLEADQYKEFRQLVIDMVLCTDMSLHFQQIKNMKTMISMPESIDKTKALSLIVHCADIS
HPAKEWALHEQWSDILCEEFFRQGDRERELNLPISPLCDRNTVVVPQSQIGFIDFIVEPS
FQVLGDMIERIVNPTQTEGVLPTDTTSPKPKSSDQETVGEQVVPRPWVEHFKENKESWSK
KLPPKT

>**SmPDE4A** - S. mansoni SmPDE4A, strain CD, translated CDS
MELRTDKVISSNDTISSSQEPQLSPQIPAVVKPRRGSIFVSALAAEQHGASSLSTDNTGS
MIVRLGRSSTGGATAATSTSSSSTNGITAVLPDSSVEGVIVTPFAQVLVSMQRIRNAFIR
LTAAQVSNKYNITTVFDSVPTGNLTPDSSDYKIIANETLEELEWCLKQLENIQTKRPVSD
MAFSKFKRLLNKELNSFGEADKSRHQISAYICETFLETEKDVETNEEIDSMLERRRSSGQ
SHNSTSGQDTNTTSKRQASGTGDQNANPNTTRTPDTSSSVSSSVIKTRIGSTGSMSTESR
KSAQLNDSSGLLTTKLPSSSKLTSQNVDDGNGPFLPIHGVETPNDNELEERFSLCLDEWG
VDIFEIDRLSNGHALTTVAYRIFQKRDLLKTFCIDPHVFVRYLLRVESTYHADVPYHNSM
HAADVLQTAHFLLQAEALDDVFSDLEILAVLFAAAIHDVDHPGVTNQFLINTGHELALQY
NDASVLENHHLYMAFKILTEKDCDIFANLGGKKRQTLRRMVIELVLATDMSKHMSLLADL
RTMVETKKVSGSGMLNLDNYADRIQILQNMIHCADLSNPAKPLRLYRKWTGRLIEEFFRQ
GDKERELSLEISPMCDRESVEVEKSQVSFIDFVCHPLWETWCDLVHPCAQLILDTLEDNR
DWYECHIKESKMKVTQLARPKLATAAEDDEEISTTSGNT

>**SmPDE4B-a1** - S. mansoni SmPDE4B, strain CD allele 1, translated CDS
MIMWIICCRKQETKKNKTKDNLQLSSKYSISYNDHRINDNQDNCKLKNDLNIHTKLSHDS
YANIENGHYPKQERRSLPDVVIPSTNLPIPRSSLQIDYETGRRSTIYLETTVKLAESESI
NLDDNYSSNIFNETFLNNIKLNMANRDFLLDKPRAHSLISSTYQKQLTNNHKGRQGRKSD
VSRSDNHEKKLISKKRLSLFKVKPLKRFRKSKDSLHSLSPQKCTIRNDKSNMISTMTCDR
STNSKTKSSLKRIKPSKLFSTNKSSHENIGITKDLNNHESCSERSEDSLGQLPMTRNSMF
KHIQTRTTNLYQTTKNFDLNKQNYLNNIEHSMTNSKNTVLRRGSLCIFSQIDEPIVTPFA
QILASLRKVRFNFILLTNVTSTRDSRFGAVQPIQSTEDGNSSSHLGCSGGQNKITASETL

EELEWCLERLENIQTHRSVSDMASSKFKKMLNKELSQFADAGQSGKQISEYICSTFLDSK
ENDPLTTTSHSSVISHNNTHGIMSNSINKDIMINNHDNPSEMNTCTPSDSIKNSESTGMY
LFKSVEPGEIIQTTTTTTTLTDNIDGDTTIGVTGDSSSSNIPHTINSTTVTIGSTTMNKS
LSKLSSSLSTLSLKMNSNVNVNENTNNNNNNNDDNNINIMMKSSGNSHTTMSIPPSGTSP
LSHQHHSECDKIHCSQTQIVPYIINSTNPKKLEDLLKTSLDLWGIDIFEVDQLTTNPLTC
IFYNIVQKRNLLQKFAIPERNLLLYMTAVEEKYNNNPYHNRVHAADVVQSTHVLLNAQSL
ESVFTDLEIFTVLFACAIHDVGHPGVTNQYLINTNDQLAILYNDSSVLENHHLAIAFSLL
GQPGHDVFENFPRKQRLSSRRMIIDMVLATDMSKHMSLLADLKTMVETKKVAGSGILTLE
NYIDRMQILQNMVHCADLSNPAKPLDLYRQWTNRVMEELFQQGDKERELGIEISPICDRN
TATIEKSQVSFIDYIVHPLWETWSDLVYPDAQTILETLEDNREWYYNQINENNNDNNAEN
DE

>**SmPDE4B-a2** - S. mansoni SmPDE4B, strain CD allele 2, translated CDS
MIMWIICCRKQETKKNKTKDNLQLSSKYSISYNDHRINDNQDNCKLKNDLNIHTKLSHDS
YANIENGHYPKQERRSLPDVVIPSTNLPIPRSSLQIDYETGRRSTIYLETTVKLAESESI
NLDDNYSSNIFNETFLNNIKLNMANRDFLLDKPRAHSLISSTYQKQLTNNHKGRQGRKSD
VSRSDNHEKKLISKKRLSLFKVKPLKRFRKSKDSLHSLSPQKCTIRNDKSNMISTMTCDR
STNSKTKSSLKRIKPSKLFSTNKSSHENIGITKDLNNHESCSERSEDSLGQLPMTRNSMF
KHIQTRTTNLYQTTKNFDLNKQNYLNNIEHSMTNSKNTVLRRGSLCIFSQIDEPIVTPFA
QILASLRKVRFNFILLTNVTSTRDSRFGAVQPIQSTEDGNSSSHLGCSGGQNKITASETL
EELEWCLERLENIQTHRSVSDMASSKFKKMLNKELSQFADAGQSGKQISEYICSTFLDSK
ENDPLTTTSHSSVISHNNTHGIMSNSINKDIMINNHDNPSEMNTCTPSDSIKNSESTGMY
LFKSVEPGEIIQTTTTTTTLTDNIDGDTTIGVTGDSSSSNIPHTCTTVTIGSTTMNKSLS
KLSSSLSTLSLKMNSNVNVNENTNNNNNNNDDNNINIMMKSSGNSHTTMSIPPSGTSPLS
HQHHSECDKIHCSQTQIVPYIINSTNPKKLEDLLKTSLDLWGIDIFEVDQLTTNPLTCIF
YNIVQKRNLLQKFAIPERNLLLYMTAVEEKYNNNPYHNRVHAADVVQSTHVLLNAQSLES
VFTDLEIFTVLFACAIHDVGHPGVTNQYLINTNDQLAILYNDSSVLENHHLAIAFSLLGQ
PGHDVFENFPRKQRLSSRRMIIDMVLATDMSKHMSLLADLKTMVETKKVAGSGILTLENY
IDRMQILQNMVHCADLSNPAKPLDLYRQWTNRVMEELFQQGDKERELGIEISPICDRNTA
TIEKSQVSFIDYIVHPLWETWSDLVYPDAQTILETLEDNREWYYNQINENNNDNNAENDE

>**SmPDE4C** - S. mansoni SmPDE4C, strain CD, translated CDS
MTTISTNIHSKSIGNSLYERRRNWRIHQMKSCMEPPINVSMTSIYNKDDDDGIRMNIMSK
KSDILCNKENWWGKTISHEPSVLITPFAQILAILNRARDFLSNYTSNSPSYSPHDPNMCS
TKSIGCDEKYVNCLHRFDVHYVNQLLNEFDWCLEVLDSLQSKRSVSSLTRMKLRSLLSQE
LAASFNQSNGKIIKKDSLKKSTTTTHNVRFDDLTLKNSSLLVHDQDNNNKRSNISQIQKR
KQQFRSCRSQVCEYICKTFLEEDDDDEDVDNNHEDDDGDAEEDNSKSISSHNPSTEKSIH
LNSPKSKLRFSDKYEERMNESSETTESTTELKSTSSTQFNDTDVISDTLKLIIILNIEHVD
HSIVENFIINNQSNLAPDLFKLDQISNHHPLSTFGFYLFMKTNVLQKLSIPSVTMLNCLR
QIESRYNSTAPFHNSIHALDVLHATYQLFQCNSLKNIFSDLETFAIFFASAIHDIDHPGL
TNQYLINTNHELALLYNDISVLENHHLHVAFKLINTQIECDFTKYFTNQQKLLFRKMVIA
LVLSTDMSKHMSLLADLKTSVEKQKAFQGNVINLDSYSARMQILECIIHAADLSNPTKPL
KIYQEWVSRIMEEMFRQGDQEKQYGIEISPMCDRETACIYSTQIGFIDYIVYPLWETMAE
LLHPGLQVLMDNITNNRNWYVKAKEEEEEEVKEENKKNNSIDQ

>**SmPDE7var-a1** - S. mansoni SmPDE7var, strain CD allele 1, translated CDS
MRRSRTDQKSGSWSHMEVDLTNKNLQRTKNSSCRRIRYRSNSFNAFVCQQSEQMAGTIQL
FYLYEDKNSKNMYNLDKSKERKKSQTVERITDEIKLSFIDRPVGHVKIKNLSNWDFNIFH
LKRTSSNYTIRDIGLQIMNEYDLFQKLKLNYFMMARIFNSIEAAYHNFNPYHTALHAADV
LQAVHCFISRSQLLTILSPTEIFASLLAAALHDADHPGVNQSYLEKTGDFLVDLYKSVSV
LEKHHAKFGLCILQENGLSNALELHEWEFVRDCFLKLIPATDITYQGVYQKQFKDLTNYH
MANPSLPFTISDRLLIMQMALKCSDISNPCRIWPICKEWAIRVCCELFCQGDRERFQWSL
QPIPTMDRTKFTLARIQNGFIRDMVKPLLTGWHEFLQNNLTLKILQNLDENLKNWLTDLS
LSSSSSTSSSSGMTNYVNRHNSLDSQLLSTLPLSNNNNNQSISSNQIKPKTIKSEISNQK
SKIELQKPFIGSIHLTHSPIQEIETMNASESLDSTTTNNNNLHSGKLHQTIFITTRVIRR
HSLPETQLAIRKTFNFSLSNKSSTVVLLKNDTQNILSLHSRNNNNLPVGKIKCQSSTFKS
PSSSLSPSSTCTVSANLLQMLYEELKHNNKSNYVLSKIDIKSIPNTNCYKDNKLLEQKVL
DLNYDRTLLRFSALAHRRSSAPITEH

>**SmPDE7var-a2** - S. mansoni SmPDE7var, strain CD allele 2, translated CDS
MRRSRTDQKSGSWSHMEVDLTNKNLQRTKNSSCRRIRYRSNSFNAFVCQQSEQMAGTIQL
FYLYEDKNSKNMYNLDKSKERKKSQTVERITDEIKLSFIDRPVGHVKIKNLSNWDFNIFH
LKRTSSNYTIRDIGLQIMNEYDLFQKLKLNYFMMARIFNSIEAAYHNFNPYHTALHAADV
LQAVHCFISRSQLLTILSPTEIFASLLAAALHDADHPGVNQSYLEKTGDFLVDLYKSVSV
LEKHHAKFGLCILQENGLSNALELHEWEFVRDCFLKLIPATDITYQGVYQKQFKDLTNYH
MANPSLPFTISDRLLIMQMALKCSDISNPCRIWPICKEWAIRVCCELFCQGDRERFQWSL
QPIPTMDRTKFTLARIQNGFIRDMVKPLLTGWHEFLQNNLTLKILQNLDENLKNWLTDLS
LSSSSSTSSSSGMTNYVNRHNSLDSQLLSTLPLSNNNNNQSISSNQIKPKTIKSEISDQK
SKIELQKPFIGSIHLTHSPIQEIETMNASESLDSTTTNNNNLHSGKLHQTIFITTRVIRR
HSLPETQLAIRKTFNFSLSNKSSTVVLLKNDTQNILSLHSRNNNNLPVGKIKCQSSTFKS
PSSSLSPSSTCTVSANLLQMLYEELKHNNKSNYVLSKIDIKSIPNTNCYKDNKLLEQKVL
DLNYDRTLLRFSALAHRRSSAPITEH

>**SmPDE8** - S. mansoni SmPDE8, strain CD, translated CDS
MKSETRQPTIKESVDNEITSTDELKANVIDSVTVLHEQGKKFREFISVRKNRNSDTNNIV
SLSALLHSRCPDFSSPMCKVIGILNSARVRSPLPVAKDLQKAINLICSSNVFVDQIMKPL
SRTNDPITADLIEGLITGSNLAREPENLLKLRSLAKSLKGSENASTTLSTLKNSPEIEAC
LSNFDKWDFNIFDLERITNKKPLTCLGMKILDSFNALSVLRIPSQILVGWLTVIEEHYHV
DNPYHNATHAGDVLQASAYFLQHSLIRSICTNIDEVATLLAAIVHDVDHPGKTNPFLVNS
NDPLAILYNDIAVLESHHAAVSFELTLRSPDINIFQNLTREEYRTMRSYIVDMVLATEMV
RHFDIVTKFVNTLSKPMLAKNRHHDRSSVGSMSSMESCSMGMTISHSTSPSPGQERISST
LENLSTAENRTLIKRLIIKCSDVNNPTRPLSICKEWATRIAEEYFCQTEEEKRRNLPIVM
PNFDRQTCNISQSQLSFIDFFLKGMFSGFDCVFPIPELMNNLENNTTYWASNIDREKKQH
GTCPVELKPTTIHQE

>**SmPDE9A** - S. mansoni SmPDE9A, strain CD, translated CDS
MGSVISKLTPKVIYLLINGNIERILITLSCTSFEIHDLICILSNVPKSSNIIITDVNGLH
IPCSGSMLANTYNTPYTVTITQPSEPSEISLIVRMFESIIKQINDTMKISDLKNEFTERI
QLLEQRVMVESDRYNDIDVIKKELKQLKIQIHERKTGLTNIGSERTYLGNIRLSNDGVKI
LELQNLPIFEKYTLTQSTIDFLKKPTFDIWHWEPNEMLALLEHMYNELGVVSEFNINPLT
LKRWLLSIQANYRNNPFHNFRHCFCVAQMMYGILYLCGLNNDFSREELGILLTAAVCHDL
DHPGYSNSYQINARTELAIRYNDISPLENHHCAVAFSILNHPELNIFANVNQEVFRRIRQ
GMTSLILSTDMARHGEILETMRRHLEEGFSMNKKEHRETFKMVLIKCCDISNEVRPLSVS
EPWVDCLLEEYFNQSDREKLEGLPVAPFMDREKVTKPTAQIGFIKFVLIPMFQTVASVYP
IIDELMVTQLKSALERYEKMLAEEEETKRNLQLNEAD

>SmPDE9B-a1 - S. mansoni SmPDE9B, strain CD allele 1, translated CDS
MNATKCEEDITSDHKISNITISQNNTNDNTRTICKPAIQTTKTTTTLPRKLNADNDDNNT
NSFLSLYTRCIKNPNRRKKKQLDNNNNNNNNSNHNHQSHFCLNFCRNCSSNTPSSSSSSS
SSSTATTEAGASVAVAATTGRKESNSFSLPFPLDNKSHDSLISYSSKITNNTNFTNDISS
PGTKVTLTPNHTNNIIMSVSNCSNQFNSISVDPDKKDNHKIDAQNTSVHINKRQTADKFH
SNTTEYQSKSNMTTTVMTSIPTTTTIEPINVHKKINPSLMIADNSAYFNDNHENLLEISK
LPIYDSTKLCIKCQKNIQLVSKDYEHILLLTNNQGKHEKITVNNNSIINNNNNGNGYMEH
TTEMIDDIKSKKIKEMESDIEEYVKHVQHIFDHINLTKDQFCQTDSKSNYIIDNKQLWDA
FNTSYANQEKIFRPSHNDINIYHLDDDMNNGDVNDDNDEEIIEAHYDNVHSYRKCNHFSV
DKMDKKRISKIYRQLHNLRCQVESFSYLSWLGLTAEQPPTQKVLVPGFNAPAPNPQMHLI
RRSDADSRRNIQEFKLLCKEPVSKEDLIELRSSTFNNWSRTDAQLIRLVREMFQELGFIE
HYNIQLHQLDLWLTDIYRRYNRVPFHNYKHAFMVTQMCYVLIWGGNLTNLLDIDDQLILI
VSAICHDLDHPGFNNAYQINAGTVLAMRYNDQSPLENHHTAVAFDLLSHKEVDPFSHLST
TTRQRIRKGVIRCILATDMSRHNEILDEFNRQVLTDLNAAWEIDPNTKKPTWVMNKTQKD
LVMVIILKISDISNEARPLNVAGPWINRLLAEFFHQSDYEKLVGLPVAPFMDRHKVTKSA
SQCGFIRFVILPLFESLAKLLPEVKPIIVQPALEQLAYYTDLHNNEEKKTNTENQKSNTN
EHQNGNNNNHNEKEHSK

>SmPDE9B-a2 - S. mansoni SmPDE9B, strain CD allele 2, translated CDS
MNATKCEEDITSDHKISNITISQNNTNDNTRTICKPAIQTTKTTTTLPRKLNADNDDNNT
NSFLSLYTRCIKNPNRRKKKQLDNNNNTNSNHNHQSHFCLNFCRNCSSNTPSSSSSSSSS
STATTEAGASVAVAATTGRKESNSFSLPFPLDNKSHDSLISYSSKITNNTNFTNDISSPG
TKVTLTPNHTNNIIMSVSNCSNQFNSISVDPDKKDNHKIDAQNTSVHINKRQTADKFHSN
TTEYQSKSNMTTTVMTSIPTTTTIEPINVHKKINPSLMIADNSAYFNDNHENLLEISKLP
IYDSTKLCIKCQKNIQLVSKDYEHILLLTNNQGKHEKITVNNNSIINNNNNGNGYMEHTT
EMIDDIKSKKIKEMESDIEEYVKHVQHIFDHINLTKDQFCQTDSKSNYIIDNKQLWDAFN
TSYANQEKIFRPSHNDINIYHLDDDMNNGDVNDDNDEEIIEAHYDNVHSYRKCNHFSVDK
MDKKRISKIYRQLHNLRCQVESFSYLSWLGLTAEQPPTQKVLVPGFNAPAPNPQMHLIRR
SDADSRRNIQEFKLLCKEPVSKEDLIELRSSTFNNWSRTDAQLIRLVREMFQELGFIEHY
NIQLHQLDLWLTDIYRRYNRVPFHNYKHAFMVTQMCYVLIWGGNLTNLLDIDDQLILIVS
AICHDLDHPGFNNAYQINAGTVLAMRYNDQSPLENHHTAVAFDLLSHKEVDPFSHLSTTT
RQRIRKGVIRCILATDMSRHNEILDEFNRQVLTDLNAAWEIDPNTKKPTWVMNKTQKDLV
MVIILKISDISNEARPLNVAGPWINRLLAEFFHQSDYEKLVGLPVAPFMDRHKVTKSASQ
CGFIRFVILPLFESLAKLLPEVKPIIVQPALEQLAYYTDLHNNEEKKTNTENQKSNTNEH
QNGNNNNHNEKEHSK

>SmPDE9C - S. mansoni SmPDE9C, strain CD, translated CDS
MMFKRLIRCHVKSSRTPPNKDGTNNKIHLPTKCTTWLFSTISSSSKISTTSDIEASKSTE
TCLVDNSSKTNNCNETCNLINTQHPNDSQRISCNPYCPDPEGIKLPFIHFTKVRNQFLAI
RSQSISSSIKEQLKSHSFNNWLYSDAELINFVKFMFVDLNLPELCHFSIDTLENWIFSTY
SRYNNVPFHNFKHAFMVTQMMYCIIKMVNLPLYLSSVDLLILLFSALSHDLDHPGFTNSY
QINSGTWLALRYNDISPLENHHCMTAFDLITNNPTANIISGLTPNESRHFRRSVIRCILS
TDMAIHSECLSQFQVLRKQVYLNCQSLSIDMSSSSISSIKHNHQNIDHSCCNNNNQQKKL
KNPDSIFPSLQLSSSYIGYSSPSPTQQQPTLTTSHDHYHRHQVNHNDNDETGVDRQNRSE
KYEQYNSCINQHVNNNNNNVVNFGGDNDSDHNGDDINNNDYGHINSSLIQSSLISLINQE
PEYLLRLLMILLKVCDISNEIRSPLVADAWVDCLFNEFFLQAAAEKQAGLPVAPHMDPDL
VVKSNSQLNFLHSILIPLVKELTYIFRELHVLLESAHRRSEHFFQIKQYELAQQVVDSNC
CSTTVTTSTSSTLPITTITSTSVCHVNKSNVRVSLLLLLFN

>**SmPDE11-a1** - S. mansoni SmPDE11, strain CD allele 1, translated CDS
MSSLVRMCELCGGHIGEQSELSFEDMVTNWLDENPEFTFKYFVKSASPSMVEAWANGRNH
GEYDCLFDNSITVMDEHNDKNDDKTPVTTSLSLPIRKISSQDLELTYDKRILSSNEDGKP
TFINSVFFPFPSNEHIDSNTSSVRQTVASPSRPTHLTERDLISELALDICRELDVTSLSF
KIVQNVCRLINADRGSFFLVEKSRSTGEDVLVSKLFDITPECIFDDVLQRCSSNHIIVPF
NVGVTGYVARTGDYANIPDAYADPRFDDSVDRVTGYKTRCLLCMPIKNVDGKVLGVALVI
NKKVPSDQHHDQSINSVQPSSCESESISKHASFTEEDVKIFQSYVTFCGIGLHNAQIYEQ
SRLETYRNQVLLELARIIFSEQLDITRLIYSVLSHTICLLQCQRCQLLLVKTTSSMSSYS
SIDEMGPFHDHFSQIFELAWNEKSDSPDVKKKKHSIDEARFPVQLDLAIHVLQTGESLHV
NINGTTTISNNNNKNKNNNNEYKKIDETLEEDLDPVWRSRSVLCMPIKHSDGKVLAVCII
TNKSTVDLRINNNNFSQQVTRHFDFKPVTDNVQCSSNDNLSSKEPVTMSTSINDWSGIFT
YSDEFLFEAFALFVGLGISNSQLYEKAIRSAAKQKVIMDVLSYHATAPTSEAKRLATSLI
PTMRFYHLDKFSFTDVRLSDEDTLKACIRMFQEMNFMKSIHFDQLSFARWLLSVRKNYRE
VTYHNWRHAFNVTQTMFCILLKGDFQSVFTDLECLALLTACLSHDIDHRGTDNQFQIKTM
SPLAKLYSTSVLEHHHFNQFMMILSIKGNNFLCNLNRSEYDTVVKLIREAILATDLSRYF
ARLPKFQQTLHHLKEINDQSTNLWRNDREQRLLLGCMFMTACDVSAITKPWPVQKLTAEM
VANEFFEQGDLEKERLNVTPAALMDRERSNELPKLQVSFIDSICVPIYEAIVQVSPNFEP
LLKGCKRNRTCWLILSENGEVDHSLYGLNDESETIPGTSTETTAKSITTTTAEGSTQLEN
VITSTSNIITSLGTLNPDSAERIAKSKPFTSSNIRRASVSSMTTVGGQEPPIQCQSSVES
K

>**SmPDE11-a2** - S. mansoni SmPDE11, strain CD allele 2, translated CDS
MSSLVGMCELCGGHIGEQSELSFEDMITNWLDENPEFTFKYFVKSASPSMVEAWANGRNH
GEYDCLFDNSITVMDEHNDKNDDKTPVTTSLSLPIRKISSQDLELTYDKRILSSNEDGKP
TFINSVFFPFPSNEHIDSNTSSVRQTVASPSRPTHLTERDLISELALDICRELDVTSLSF
KIVQNVCRLINADRGSFFLVEKSRSTGEDVLVSKLFDITPECIFDDVLQRCSSNHIIVPF
NVGVTGYVARTGDYANIPDAYADPRFDDSVDRVTGYKTRCLLCMPIKNVDGKVLGVALVI
NKKVPSDQHHDQSINSVQPSSCESESISKHASFTEEDVKIFQSYVTFCGIGLHNAQIYEQ
SRLETYRNQVLLELARIIFSEQLDITRLIYSVLSHTICLLQCQRCQLLLVKTTSSMSSYS
SIDEMGPFHDHFSQIFELAWNEKSDSPDVKKKKHSIDEARFPVQLDLAIHVLQTGESLHV
NINGTTTISNNYNKNKNNNNEYKKIDETLEEDLDPVWRSRSVLCMPIKHSDGKVLAVCII
TNKSTVDLRINNNNFSQQVTRHFDFKPVTDNVQCSSNDNLSSKEPVTMSTSINDWSGIFT
YSDEFLFEAFALFVGLGISNSQLYEKAIRSAAKQKVIMDVLSYHATAPTSEAKRLATSLI
PTMRFYHLDKFSFTDVRLSDEDTLKACIRMFQEMNFMKSIHFDQLSFARWLLSVRKNYRE
VTYHNWRHAFNVTQTMFCILLKGDFQSVFTDLECLALLTACLSHDIDHRGTDNQFQIKTM
SPLAKLYSTSVLEHHHFNQFMMILSIKGNNFLCNLNRSEYDTVVKLIREAILATDLSRYF
ARLPKFQQTLHHLKEINDQSTNLWRNDREQRLLLGCMFMTACDVSAITKPWPVQKLTAEM
VANEFFEQGDLEKERLNVTPAALMDRERSNELPKLQVSFIDSICVPIYEAIVQVSPNFEP
LLKGCKRNRTCWLILSENGEVDHSLYGLNDESETIPGTSTETTAKSITTTTTEGSTQLEN
VITSTSNIITSLGTLNPDSAERIAKSKPFTSSNIRRASVSSMTTVGGQEPPIQCQSSVES
K
